# Practical implementation of DNA methylation and copy-number-based CNS tumor diagnostics: the Heidelberg experience

**DOI:** 10.1007/s00401-018-1879-y

**Published:** 2018-07-02

**Authors:** David Capper, Damian Stichel, Felix Sahm, David T. W. Jones, Daniel Schrimpf, Martin Sill, Simone Schmid, Volker Hovestadt, David E. Reuss, Christian Koelsche, Annekathrin Reinhardt, Annika K. Wefers, Kristin Huang, Philipp Sievers, Azadeh Ebrahimi, Anne Schöler, Daniel Teichmann, Arend Koch, Daniel Hänggi, Andreas Unterberg, Michael Platten, Wolfgang Wick, Olaf Witt, Till Milde, Andrey Korshunov, Stefan M. Pfister, Andreas von Deimling

**Affiliations:** 1 Department of Neuropathology, University Hospital Heidelberg, Heidelberg, Germany; 2 Clinical Cooperation Unit Neuropathology, German Cancer Consortium (DKTK), German Cancer Research Center (DKFZ), Heidelberg, Germany; 3 Department of Neuropathology, Charité Universitätsmedizin Berlin, Corporate Member of Freie Universität Berlin, Humboldt-Universität zu Berlin and Berlin Institute of Health, Berlin, Germany; 4 German Cancer Consortium (DKTK), Partner Site Berlin, German Cancer Research Center (DKFZ), Heidelberg, Germany; 5 Hopp Children’s Cancer Center, at the NCT Heidelberg (KiTZ), Heidelberg, Germany; 6 Pediatric Glioma Research Group, German Cancer Research Center (DKFZ), Heidelberg, Germany; 7 Division of Pediatric Neurooncology, German Cancer Research Center (DKFZ), Heidelberg, Germany; 8 Department of Pathology and Center for Cancer Research, Massachusetts General Hospital and Harvard Medical School, Boston, MA 02114 USA; 9 Broad Institute of Harvard and MIT, Cambridge, MA 02142 USA; 10 Department of Neurosurgery, University Medical Center Mannheim, University of Heidelberg, Mannheim, Germany; 11 Department of Neurosurgery, Heidelberg University Hospital, Heidelberg, Germany; 12 Clinical Cooperation Unit Neuroimmunology and Brain Tumor Immunology, German Cancer Consortium (DKTK), German Cancer Research Center (DKFZ), Heidelberg, Germany; 13 Department of Neurology, Universitätsmedizin Mannheim, Medical Faculty Mannheim, Heidelberg University, Mannheim, Germany; 14 Department of Neurology, Heidelberg University Hospital, Heidelberg, Germany; 15 Department of Pediatric Oncology, Hematology, Immunology and Pulmonology, Heidelberg University Hospital, Heidelberg, Germany; 16 CCU Pediatric Oncology (G340), German Cancer Research Center (DKFZ), German Cancer Consortium (DKTK), Heidelberg, Germany; 17 Department of General Pathology, Institute of Pathology, Heidelberg University Hospital, Heidelberg, Germany; 18 Clinical Cooperation Unit Neurooncology, German Cancer Research Center (DKFZ), Heidelberg, Germany

**Keywords:** DNA methylation, EPIC array, Tumor classification, Copy-number variation

## Abstract

Recently, we described a machine learning approach for classification of central nervous system tumors based on the analysis of genome-wide DNA methylation patterns [6]. Here, we report on DNA methylation-based central nervous system (CNS) tumor diagnostics conducted in our institution between the years 2015 and 2018. In this period, more than 1000 tumors from the neurosurgical departments in Heidelberg and Mannheim and more than 1000 tumors referred from external institutions were subjected to DNA methylation analysis for diagnostic purposes. We describe our current approach to the integrated diagnosis of CNS tumors with a focus on constellations with conflicts between morphological and molecular genetic findings. We further describe the benefit of integrating DNA copy-number alterations into diagnostic considerations and provide a catalog of copy-number changes for individual DNA methylation classes. We also point to several pitfalls accompanying the diagnostic implementation of DNA methylation profiling and give practical suggestions for recurring diagnostic scenarios.

**Electronic supplementary material:**

The online version of this article (10.1007/s00401-018-1879-y) contains supplementary material, which is available to authorized users.

## Introduction

DNA methylation-based tumor classification has emerged as a promising tool to dissect tumor classes and to improve diagnostic accuracy [6, 32, 45, 46]. The underlying concept is that cellular differentiation is driven by gene expression programs dependent on transcriptional control. DNA methylation at gene promoters and enhancer regions is crucial for coordinating such programs. In cancer, the genome-wide DNA methylation pattern (the “methylome”) likely represents a combination of both the cell of origin and somatically acquired DNA methylation changes [10, 14]. Interestingly, the dynamics of the methylome crucial for cellular differentiation in non-transformed cells seem to be somewhat arrested in cancer cells and DNA methylation signatures likely remain relatively stable during the course of disease [32]. Therefore, analysis of tumoral DNA methylation patterns may represent an approximation of the DNA methylation pattern of the early transformed cell and may even be close to the patterns of the cell of origin. Analysis of DNA methylation can be performed by readily available tools.

Application of this method has shown that many accepted WHO central nervous system (CNS) tumor entities can be more precisely defined by DNA methylation profiling than by morphological features: medulloblastomas are separated into four major clinically relevant sub-groups [48], ependymomas grouped by DNA methylation profiles are clinically more homogenous than what WHO classification and grading can accomplish [32], and supratentorial primitive neuroectodermal tumors (PNET) have been demonstrated to fall into very different sub-entities [45]. Consequently, the potential of methylation-based characterization has been expanded to other CNS tumor entities and the overarching concept and feasibility has just been published [6].

We introduced this technique in our routine diagnostic workup of CNS tumors in 2015. To assist in the availability of this approach for the community, we have constructed a webpage allowing the upload of methylation data and providing an analysis based on the underlying reference tumors and algorithms (www.molecularneuropathology.org).

The DNA methylation-based CNS tumor classifier (the “Classifier”) does not rely on the copy-number variation (CNV) pattern of a given tumor for classification. However, constellations of losses or gains of chromosomal regions are of high diagnostic impact in some instances. For example, combined loss of 1p/19q by now is a prerequisite for diagnosing canonical oligodendroglioma [28] and the combination of Chr.7 gain, Chr.10 loss, and/or *EGFR* amplification is characteristic for glioblastoma [46]. We, therefore, provide an overview of CNV profiles across the CNS tumor entities currently included in the Classifier and how these profiles might be utilized for diagnostic decisions.

Currently, DNA methylation-based CNS tumor classification is being introduced in several diagnostic institutions. Here we describe the experience with this approach in Heidelberg and how it is implemented within the recently introduced WHO concept of an “integrated diagnosis”. We deliberately give a focus on constellations with conflicting morphological and molecular findings or other confusing aspects. Since, for many of these constellations, a sound scientific basis of how to best proceed is not yet available, we formulate suggestions as to how we go about these situations on a working basis. We expect that many of these suggestions will be replaced by more sound recommendations in the future.

## Materials and methods

### DNA methylation-based tumor analysis—the Classifier and copy-number analysis

All methylation data were generated using the Illumina HumanMethylation450 (450k) or MethylationEPIC (850k) array platforms. Unsupervised clustering of methylation array data from a previously established reference set of unambiguously diagnosed CNS tumors (“the reference cohort”) revealed clear formation of separate tumor groups. The “Classifier” tool was developed based on a random forest algorithm. The current version v11b4 of the Classifier is based on the analysis of 10,000 CpG sites present on both the 450k and the EPIC arrays. An in-depth description on the theoretical background and development of this tool is given elsewhere [6].

The error rate of the Classifier calculated by a cross-validation analysis of the reference cohort was estimated to be approximately 1%. However, for statistical reasons in this cross validation, the class prediction was defined as “class with highest score” and did not include the threshold of ≥ 0.9 as an additional requirement for a correct prediction (for further information on the threshold see below). Therefore, we assume that many of the cases constituting the 1% “technical” error rate would actually resolve as cases with a calibrated score below ≥ 0.9, and we have not yet identified cases with such a technical error scoring in our routine workup.

The same set of data generated by employing the Illumina 450k or Illumina 850k/EPIC arrays can be used to calculate copy-number alterations using the ‘conumee’ package for R (http://bioconductor.org/packages/conumee).

### Patients

More than 1000 specimens from patients of the University Hospitals of Heidelberg and Mannheim operated between 2015 and 2018 have been subjected to DNA methylation analysis. There was a bias for rare tumor entities, diagnostically difficult cases and pediatric patients. In addition, more than 1000 referrals from outside hospitals have undergone the same procedure.

### Sample preparation

For methylation analysis, we aimed at a tumor cell content of 70% or more. Both fresh frozen and FFPE samples were included. Fresh frozen tissue was examined by frozen section to ensure sufficient tumor cell content. Shaves were then taken and processed according to protocols for the Invisorb Genomic DNA Kit II (Stratec Molecular, Berlin). DNA was dissolved in ddH_2_O to a concentration of approximately 25 ng/µl. When using FFPE tissue, suitable regions on the H&E section were marked, and for most cases, a punch of 2 mm diameter and up to 3 mm depth was taken from the corresponding position of the paraffin block. For the remaining cases, unstained sections were cut and the corresponding tumor areas were macrodissected. Following deparaffinization, DNA was extracted with the Maxwell^®^ 16FFPE Plus LEV DNA Kit (Promega, Fitchburg, USA) and a Maxwell DNA extractor. In our experience, old age alone of the FFPE material did not preclude extraction of suitable DNA. More important appears to be the quality and composition of the fixative. We prefer material fixed in 4% buffered paraformaldehyde. We did not test fixatives with a hydrolytic effect on DNA such as Bouin’s solution.

### Methylation analysis

Of every sample, approximately 250 ng of genomic DNA were processed for methylation analysis using the Illumina starter equipment (Illumina, San Diego) and the corresponding reagents. DNA quantification was performed with the Qubit^®^ dsDNA BR Assay Kit (Thermo Fisher Scientific, Waltham, USA). Bisulfite conversion was facilitated with the Zymo EZ Methylation Kit (Zymo Research Irvine, USA) followed by purification with Zymo DNA Clean Kit (Zymo Research Irvine, USA). From there on, DNA from native and FFPE tissue required different procedures. DNA from native DNA could directly be subjected to hybridization, while DNA from FFPE tissue needed treatment with the Infinium HD FFPE Restore Kit prior to hybridization to the Infinium BeadChip (Illumina, San Diego). Subsequently, the bead chips were scanned by the iScan (Illumina, San Diego).

### Data processing

The output data (.idat files) from the iScan reader were checked for general quality measures as indicated by the manufacturer. Thereafter, the.idat files were uploaded to the “Classifier” providing a methylation-based classification and a chromosomal copy-number plot. The underlying algorithms have been previously published [6]. The Classifier in its current version recognizes 82 distinct CNS tumor entities. More information is available at www.molecularneuropathology.org. The copy-number plots were generated from the same raw data using the ‘conumee’ R package in Bioconductor (http://www.bioconductor.orgpackages/release/bioc/html/conumee.html).

## Results and discussion

### Methodology background

Prior to presenting and discussing our experience with methylation analysis on more than 2000 diagnostic CNS tumor cases, we would like to briefly outline the concept of the methylation-based classification (the Classifier) and CNV analysis.

### The Classifier

The principal output of the Classifier is a list of the predicted class membership probabilities for every class currently included in the Classifier (v11b4: 82 tumor classes, 9 non-tumorous classes). These probabilities are referred to as the “calibrated” Classifier scores. The combined calibrated scores of all 91 methylation classes add up to 1. The automatically generated website report currently only lists calibrated scores above 0.3, but a listing of all scores can also be downloaded from the website. For a valid classification, we have proposed a default threshold value of ≥ 0.9 that has to be reached. As all calibrated scores add up to 1, this implies that the calibrated scores of the remaining 90 classes add up to less than 0.1 in such a classifiable tumor.

Over the last 4 years, the Classifier experienced continuous modification with more tumor entities being recognized by each update. Such an evolution will also continue in future, as our knowledge on novel classes grows. A table of the tumor methylation classes recognized by the current version of the Classifier can be obtained at www.molecularneuropathology.org.

During cross validation of the Classifier, eight methylation class families were designated consisting of two-to-six individual closely related methylation classes. For cases falling into a methylation class family, two output scores are generated: one “class score” (not different to cases without a family) and a second “family score” representing the sum of the combined scores belonging to a methylation class family. Since the calibrated scores represent probability estimates, it is straightforward to apply the sum rule in probability theory to sum up the individual class probabilities to get a probability estimate for the family. For example, an IDH wild-type (IDH wt) glioblastoma may have a class score of 0.6 for the methylation class GBM, RTK I. The case, additionally, scored with 0.2 for GBM, MES, and 0.07 for GBM, RTK II, 0.04 for GBM, MID, 0.01 for GBM, RTK III, and 0.01 for GBM, MYCN. The GBM family score would then be the sum of all the above class scores (0.93). As this score would be ≥ 0.9, the case is considered classifiable as belonging to the GBM IDH wt family (score has to be ≥ 0.9 as usual). Thus, the introduction of methylation class families allows a single threshold level for all methylation classes and families. If the subclass within a family is of interest (e.g., in IDH gliomas), we use a calibrated cut-off score of 0.5 for prediction of the most likely subclass. Therefore, the case in the above example would be considered a glioblastoma, IDH wt (family score 0.93) of the RTK I subclass (class score 0.6). The subclass cut-off value of 0.5 was chosen arbitrarily. The validity of this cutoff is not easy to assess as for most subclasses DNA methylation profiling is currently the only available method to identify the subclass. The exception to this is 1p/19q codeleted oligodendroglioma that is part of the methylation class family IDH glioma. These tumors can also be identified by the CNV profile. For these, the 0.5 cutoff seems to perform well.

### Interpretation of calibrated Classifier scores between 0.3 and 0.9

For the determination of the common calibrated score threshold, the trade-off between sensitivity and specificity has to be considered. For the cases of the Classifier reference cohort, the optimal trade-off between sensitivity and specificity (maximization of the Youden index) was reached at a calibrated score of 0.84, maximum specificity was reached at 0.96 [6]. After some non-reference cohort test cases, we decided to implement a threshold of ≥ 0.9 that is in the middle between these two values. However, it should be mentioned that, for many medical tests, the Youden index is preferred, so there is also a rational for using the less conservative cutoff of 0.84 as a threshold.

In our diagnostic experience, calibrated scores between 0.3 and 0.9 will be encountered on a regular basis. If this occurs within the setting of a low-tumor cell content, we may accept a lower score as an indication of a specific diagnosis (see also the below paragraph “DNA methylation analysis of infiltration zone of diffuse gliomas or highly inflamed tumors”). For cases with high tumor cell content and low scores, this seems more problematic. As indicated above, we would likely accept a calibrated score down to 0.84 (maximized Youden index) as valid classification if nothing else strongly speaks against such an interpretation. Scores below 0.5, we would generally discard. For cases with high tumor cell content and scoring between 0.5 and 0.84, it seems problematic to make general recommendations. We would likely see such a score as a suggestion that a case may be in some way related to a certain methylation class and would try to find further evidence of such a relation [e.g., sequencing of BRAF in a case with a methylation class (anaplastic) pleomorphic xanthoastrocytoma (PXA) calibrated score of 0.75].

### Copy-number variation (CNV) analysis

**Fig. 1:**
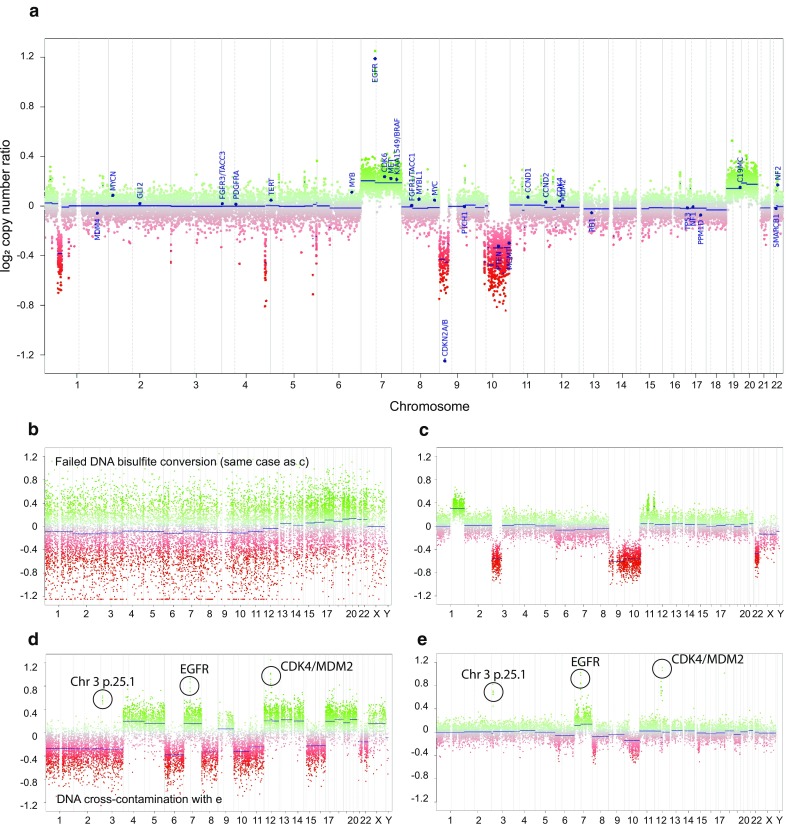
CNV plots calculated from DNA methylation array data. **a** Example of a typical CNV plot of a glioblastoma, IDH wt (subclass receptor tyrosine kinase (RTK) II). Depiction of Chromosome 1–22 with the p-arm (left) and the q-arm (right) separated by a dotted line. Gains/amplifications represent positive, losses negative deviations from the baseline. The probes of the array are combined in 8000 bins (green or red dots). For assessment of relevant deviations from the baseline, we, in general, only consider deviations of the dark blue line that represents an average over several dots and not individual colored dots. This case shows a gain of chromosomes Chr.7, 19, and 20 likely representing trisomies and several sub-chromosomal losses of one chromosomal copy (the largest of Chr.10q and several smaller ones, e.g., two on Chr.1p and one on Chr.4q). In addition, the case shows an amplification of *EGFR* (highly focal shifting of the blue line accompanied by a row of single green dots including the EGFR locus, usually above a log2 value of 0.4) and a homozygous deletion of *CDKN2A/B* (focal shifting of the blue line accompanied by a row or single red dots including the CDKN2A/B locus usually below a log2 value of 0.4). Besides *EGFR* and *CDKN2A/B,* 27 other gene regions are highlighted by default by the gene name and a blue dot for easier identification of possible copy-number alterations. The highlighting does not indicate relevant changes per se. Low-tumor cell content or subclonal alterations may reduce the amplitude of blue line deviation, and thus, a general cut-off value for what deviation is a definite chromosomal change cannot be provided. **b** Example of a CNV plot of an RELA-fused ependymoma with artificial noise caused by an unsuccessful bisulfite conversion. CNV information should not be extracted from plots of this quality. **c** Reanalysis of the same case as in b) without technical issues now demonstrating a crisp CNV plot that can be clearly interpreted. In line with the Classifier result, it shows changes compatible with an RELA- fused ependymoma (see Fig. 7g for comparison). **d** Unusual CNV plot of a non-classifiable case with multiple whole chromosomal gains and losses (likely representing a carcinoma metastasis) and signs of cross contamination with DNA from the sample in **e** (glioblastoma, IDH Wt). The identical three amplifications are found in both cases. This is the result of minimal amounts of DNA exchange from e to d during array preparation

Genome-wide DNA methylation array data can also be used to perform analysis of copy-number variations (CNV), for example using the ‘conumee' R package in bioconductor [15]. By standard, every interrogated CpG is represented by two probes on the array (one for methylated and one for unmethylated). For the analysis of DNA methylation, the ratio of the intensity signal of the methylated and the sum of the methylated and unmethylated probe intensities are calculated (methylated/(methylated + unmethylated); beta-values). In contrast, for the calculation of CNV, the methylated and unmethylated signal intensities are added together and a ratio is formed against healthy reference samples that have a flat genome. This copy-number ratio is then plotted in a graph according to chromosomal location (Fig. 1a). Areas with high copy-number ratios correspond to areas with a gain of chromosomal material (e.g., by a trisomy or an amplification), areas with low copy-number ratios represent lost DNA (e.g., by a deletion). The results from the CNV analysis can be considered as independent from results of the methylation classifier, and both readouts can independently contribute to the final diagnostic interpretation.

CNV analysis provides a good overview of gross structural alterations in the tumor genome. High-level amplifications (e.g., *EGFR*, Fig. 1a, e) and homozygous deletions (e.g., *CDKN2A/B*, Figs. 1a, 4d) are usually obvious when present. Numerical chromosome aberrations [e.g., trisomies (Fig. 1a Chr.7, 19, 20), monosomies, or larger sub-chromosomal gains and losses] are unambiguous to interpret in most cases, although this may become less clear when there is an abundance of changes and/or a low-tumor cell content in the analyzed sample. Even the presence of gene fusion events may be suggested if they are associated with focal duplications or deletions (Fig. 6a–c).

The current CNV generation includes the highlighting of 29 CNS tumor relevant genes marked by a blue circle and blue gene nametags (Fig. 1a). The identical set of 29 genes is highlighted by default in each case for easier identification of copy-number alterations. The highlighting does not indicate relevant changes per se. For the analysis of regions that are not marked by default (e.g., for the identification of genes within an unusual amplified region or deletion), an additional.igv file is automatically generated and can be downloaded from the website (“download complete analysis results”, then in the “cnvp” folder). This can, for example, be visualized with the Integrative Genomics Viewer [37].

In comparison to arrays that probe single-nucleotide polymorphisms (SNP arrays), CNV analysis from DNA methylation arrays cannot rely on allele frequencies to define a copy-number neutral state baseline. Instead, in the current version of the conumee package, the baseline is defined as the line where the median absolute deviation to all data points is minimal. Thus, the baseline is close to the predominant copy-number state of a sample. This represents one limitation of using methylation arrays for CNV analysis, but this is likely to interfere only with cases that show a substantial degree of numerical chromosomal aberrations (e.g., choroid plexus tumors or esthesioneuroblastoma) where identification of the copy-number neutral state may be challenging. Despite this limitation, for most cases, CNV can be easily interpreted, and are of considerable added value in tumor entities that exhibit characteristic chromosomal alterations.

To give an overview of typical chromosomal aberrations of the DNA methylation classes, we have created summary CNV plots of all classes included in the Classifier (Supplementary File 1). Selected summary CNV plots are further demonstrated in the main manuscript. It is important to note that these summary CNV plots have to be read differently in comparison to single-case CNV plots. The y-axis of the summary plots does not contain information on the intensity of signal that is related to the amount of DNA material in each sample, but gives the % frequency of changes at that location either as gain or loss (irrespective of whether, for example, a gain is a single-copy gain or an amplification) of many combined cases. Thus, both, the focal duplication of BRAF on Chr.7 as part of a *BRAF* fusion in pilocytic astrocytomas (Fig. 5a) and the high-level amplification of C19MC on Chr.19 in embryonal tumors with multilayered rosettes (ETMR; Fig. 13f), look relatively the same in this depiction.

For simplification, we defined CNV alterations as either simple chromosomal changes (numeric chromosomal changes, e.g., a trisomy or monosomy, and whole chromosome-arm loss or gain) or complex chromosomal changes (breakage within chromosomal arm, focal loss, focal gain, amplifications, and chromothripsis) throughout the text.

### MGMT methylation status

The status for MGMT promoter methylation can be derived directly from the array data. We are employing an approach previously published [2] [1] which has been adapted for the EPIC (850k) array platform. Based on this algorithm which is also underlying the MGMT promoter methylation analysis provided by our webpage for uploaded cases, we receive the readouts “methylated”, unmethylated, or “not determinable”. DNA from cases with the classifier-based result “not determinable” is subjected to MGMT pyrosequencing frequently resolving these cases. The classifier results “methylated” and “unmethylated” show a very high concordance with MGMT pyrosequencing results [2].

### Standard quality control

Before we discuss our experience with individual tumor classes, we provide a description of how we approach the data as soon as the reports have been generated by the classifying algorithm (www.molecularneuropathology.org). These steps are general quality assessment steps that we have found valuable for the detection of handling errors that may occur during the process of array preparation and data upload.

1. We check that known patient gender is identical to the gender predicted by the algorithm for all samples of the run. If one or more samples of a batch show a mismatch, we very critically check for a possible case mix-up.
2. We check all the automatically generated CNV plots for noise. In our experience, the CNV plots are sensitive to various kinds of technical issues that may occur during the array preparation process, such that if the CNV is crisp (“low noise”) likely everything went well. We have not as yet established a standardized noise assessment, and currently assess this by eye. As recurring factors resulting in a noisy CNV, we have observed very low DNA input, poor bisulfite conversion, omitting the FFPE DNA restoration kit for FFPE samples, poor whole-genome amplification performance, and overall poor DNA quality. Extremely noisy CNV plots are easily spotted by the massive dispersion (Fig. 1b) and reanalysis is recommended (Fig. 1c). There may also be cases with only a gradual increase of noise and suitability for detection of CNV may have to be decided on a case-by-case basis for the time being. Of note, the Classifier may still be able to generate accurate classification data even for cases with relatively noisy CNV, so this seems to be less sensitive to some of the technical issues.
3. We routinely check all CNV of one run for amplifications. If a case has any amplification, we check the remaining cases for focal gains or amplifications of the identical region. If present, this may indicate a cross contamination of the two cases. Such contaminations occur more frequently than we would have expected (in our hands approximately 1 in 300 cases, Fig. 1d shows a case contaminated by DNA from case Fig. 1e). This contamination likely occurs after the DNA extraction and before the whole-genome amplification during array preparation. Because of the massive overrepresentation of the amplified regions, typically only amplifications become apparent in the contaminated sample in contrast to other chromosomal gains or losses. Amplifications likely become visible even in cases with minimal contamination. This approach obviously cannot detect cross contamination with DNA from cases without amplifications. The Classifier frequently will still perform well on such lightly cross-contaminated samples and the usability of the data has to be decided on a case-by-case basis. Still, it is highly important to be aware of such cross contamination, because the presence of an unexpected amplification may compromise the interpretation of a tumor and has the possibility to falsely override benign histology and benign methylation assessment (e.g., an *EGFR* amplification contamination in a ganglioglioma may result in a serious misinterpretation of the case).
4. We check the predicted methylation classes with respect to expected tumor diagnoses for all cases of the run for consistency. Unexpected results may indicate case mix-ups and should be investigated for patterns of systematic error (such as an array loaded in reverse order).
5. Surprising methylation classes or CNV changes (e.g., the above discussed “amplification” in a ganglioglioma) should be reanalyzed in general going back to the initial material (i.e., we generally re-extract DNA from the original paraffin block when a case has to be repeated).

### Implementation of DNA methylation profiling and CNV analysis for specific tumor classes

In the following paragraphs, we will briefly discuss all entities presented in the WHO classification of CNS tumors [28] in the order which they appear in the blue book in relation to the corresponding methylation classes as well as the most frequent copy-number variants.

### Diffuse astrocytic and oligodendroglial tumors

The Classifier recognizes tumors with an IDH mutation-associated CIMP phenotype, and thus both *IDH1-* and *IDH2*-mutated tumors are detected independent of the mutation type (including variants not detected by, for example, the IDH1 R132H antibody). Because of their close relation, a methylation class family for IDH mutant gliomas was introduced (“IDH glioma”). This family includes the methylation subclasses astrocytoma (A IDH), high-grade astrocytoma (A IDH, HG), and 1p/19q codeleted oligodendroglioma (O IDH). The association of the DNA methylation class IDH glioma and the presence of an IDH mutation in a glioma is so strong that, in our experience, this methylation class can be interpreted as proof of IDH mutation status.

**Fig. 2:**
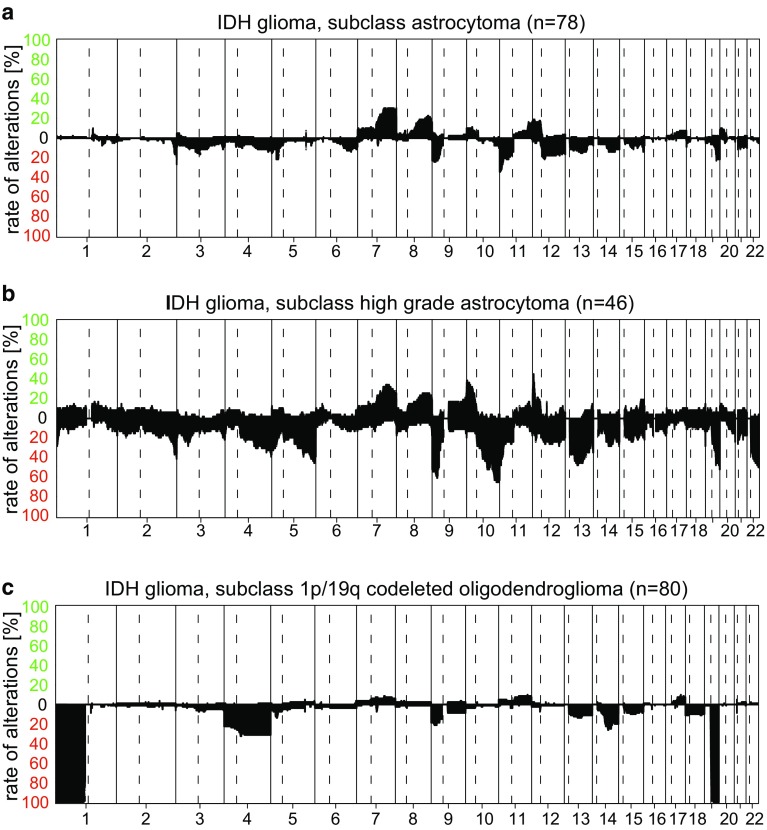
Summary CNV plots of IDH mutant diffuse astrocytic and oligodendroglial tumors. **a** IDH glioma, subclass astrocytoma; **b** IDH glioma, subclass high-grade astrocytoma; **c** IDH glioma, subclass 1p/19q codeleted oligodendroglioma

The WHO diagnosis diffuse astrocytoma IDH mutant more or less completely falls into the methylation class “A IDH”. The CNV pattern in “A IDH” is not highly characteristic (Fig. 2a). Anaplastic astrocytoma WHO grade III partially fall into the “A IDH” and partially into the “A IDH, HG” methylation subclasses. Of note, the “A IDH, HG” group also contains most of the IDH mutant glioblastomas. The CNV in “A IDH, HG” are comparable to those in “A IDH” but with frequently a higher number of total changes and also a higher degree of complex changes and frequent loss on Chr.9p including the *CDKN2A/B* locus (Fig. 2b). However, recent data provide evidence that grading of IDH mutant astrocytoma by assessing copy-number alterations is more powerful than grading by DNA methylation analysis only [44].

Oligodendroglioma and anaplastic oligodendroglioma, IDH mutant and 1p/19q codeleted are generally classified as methylation subclass “O IDH”. CNV of “O IDH” exhibits 1p/19q codeletion in all cases. The second most frequent alteration is loss of Chr.4p/q (Fig. 2c).

Oligoastrocytomas by WHO definition [28] are considered NOS and the Classifier typically either scores these tumors as “A IDH”, “A IDH, HG”, or “O IDH”. We have so far analyzed one case of a dual genotype astrocytoma/oligodendroglioma [16, 53] and, indeed, observed a different methylation class of A IDH and O IDH, respectively, in different macrodissected tumor areas and an exclusive 1p/19q codeletion in the oligodendroglial tumor regions. Currently, we believe this to be a very rare genetic constellation.

The diagnosis of diffuse astrocytoma, IDH wt and anaplastic astrocytoma, and IDH wt has been severely questioned by several recent publications [4, 35, 47, 52]. Consensus of the critique is the demonstration of mutational profiles in these tumors that closely match those of glioblastoma (or, rarely, a more pediatric-type diffuse glioma). In concordance with this, we were not able to establish a separate comprehensive IDH wt astrocytoma methylation class and the vast majority of such tumors fall into other DNA methylation classes. Most of these cases resolve as glioblastomas, and frequently, the copy-number pattern can be used to further strengthen this classification. Another group of cases may resolve as a tumor class recently described as “anaplastic astrocytoma with piloid features” that is further described in the paragraph “Other astrocytic tumors” [34].

**Fig. 3:**
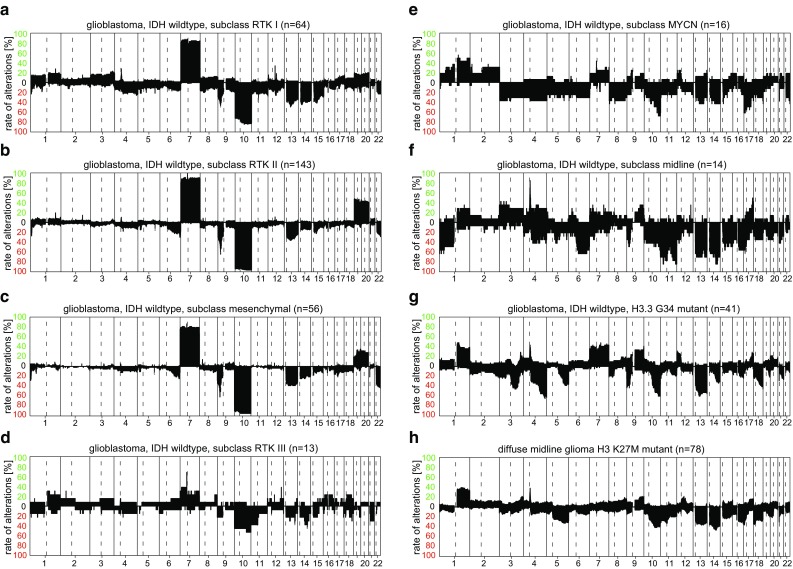
Summary CNV plots of seven IDH wt glioblastoma methylation classes (**a**–**g**) and diffuse midline glioma, H3 K27M mutant (**h**)

WHO Glioblastoma, IDH wt is currently represented by seven classes in the Classifier. One (GBM, G34) is characterized by the presence of a G34 H3.3 histone mutation and has previously been defined [23, 46]. The other 6 are more closely related and were grouped into a methylation class family (“Glioblastoma, IDH wt”; members: “GBM, RTK I”, “GBM, RTK II”, “GBM, RTK III”, “GBM, MES”, “GBM, MID”, “GBM, MYCN”). The eighth related class “DMG, K27” directly translates to the diffuse midline glioma, H3 K27 mutant of the WHO classification. However, in cases where this is of relevance, *H3F3A, HIST1H3B,* and *HIST1H3C* mutations should be confirmed by other methods, because both methylation classes “GBM, G34” and “DMG, K27” contain a small fraction of tumors without apparent mutations in these three genes. Giant cell glioblastoma and gliosarcoma associate with various members of the methylation class family “Glioblastoma, IDH wt” and do not currently seem to represent distinct molecular entities. Figure 3a–h shows the summary plot for these eight classes. Epithelioid glioblastomas are more problematic. A recent DNA methylation-based analysis of 64 epithelioid glioblastomas demonstrated that they stratify into three molecularly and clinically distinct groups: PXA-like tumors with favorable prognosis, IDH wt glioblastoma-like tumors with poor prognosis, and RTK I pediatric glioblastoma-like neoplasms with intermediate prognosis [24].

### Diffuse astrocytic and oligodendroglial tumors—suggestions for specific scenarios

#### 1p/19q codeleted tumor but highest score is for A IDH

In particular, when tumor cell content is low, we have observed a shift from oligodendroglioma scores towards astrocytoma scores. In some instances, this may result in a higher score for an astrocytoma despite a 1p/19q codeletion. In such tumors, we follow the copy-number pattern (as per the WHO classification) and interpret such cases as oligodendroglioma. We would pay additional attention to the ATRX immunohistochemistry [36] (that should be retained in such a setting) and/or *TERT* promoter mutation status (almost always mutated in oligodendroglioma).

#### Astrocytoma, IDH wt with low proliferation but GBM, IDH wt classifier score

**Fig. 4:**
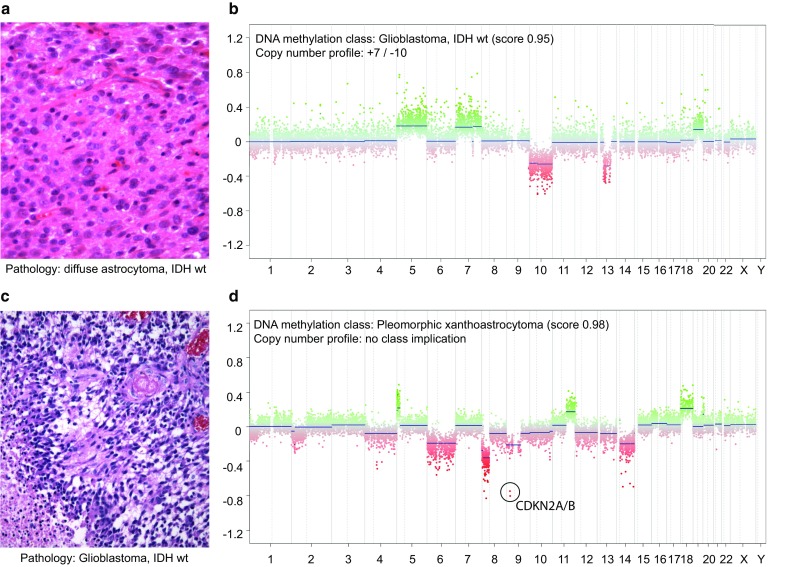
Example of cases with conflicting pathological and molecular results. **a** H&E of an IDH wt, moderately cell dense, moderately pleomorphic diffuse glioma diagnosed as diffuse astrocytoma, IDH wt (WHO grade II). **b** Methylation class and CNV plot correspond to a glioblastoma, IDH wt. The integrated diagnosis was also glioblastoma, IDH wt. Many of these cases are from small biopsies. **c** H&E of a pleomorphic necrotizing tumor with the pathological diagnosis of glioblastoma, IDH wt. **d** Methylation class corresponds to an (anaplastic) pleomorphic xanthoastrocytoma; the copy-number profile would be compatible with both diagnoses (and is not highly characteristic for either). A BRAF V600 mutation was not present. This conflict could not be resolved and the case was diagnosed as malignant glioma, histologically glioblastoma, methylation profile (anaplastic) PXA, NEC for the time being

IDH wt anaplastic astrocytomas in the vast majority of cases reclassify as glioblastomas, IDH wt, and we generally follow this in the integrated diagnosis [35]. It is more problematic when a diffuse astrocytoma with low proliferation is classified as glioblastoma (Fig. 4a, b); we have recurrently observed this particularly in small biopsies from stereotactic surgery and infiltration zones of glioblastomas, IDH wt. In case the CNV shows prototypic glioblastoma changes (in particular amplification of *EGFR* or other amplifications, combined gain of Chr.7, and loss of Chr.10), we would consider the case a glioblastoma, IDH wt. We are cautious, in case no complex chromosomal changes are observed and even more so if only an isolated gain of Chr.7 or loss of Chr.10 is present. In such cases, we would discuss a likely close relation to glioblastoma but would indicate that the available data are not sufficient to definitely classify this tumor and we would give a descriptive diagnosis of “diffuse glioma with molecular features of glioblastoma”. More detailed recommendations for such scenarios are currently being developed by the “Consortium to Inform Molecular and Practical Approaches to CNS Tumor Taxonomy” (cIMPACT) [29] with a publication expected in the near future.

#### Diffuse astrocytoma, IDH wt classified as other non-GBM tumor

As long as a calibrated score of ≥ 0.9 is reached, we would reconsider histopathology with the question whether the tumor could also be compatible with the suggested methylation class (in our experience, it frequently is). Typical classes that these cases resolve into are ganglioglioma (further covered in the paragraph “Neuronal and mixed neuronal-glial tumors”; an example is shown in Fig. 10a, b), pilocytic astrocytoma, dysembryoplastic neuroepithelial tumor (DNT) and MYB/MYBL1-associated low-grade gliomas and anaplastic astrocytoma with piloid features (further covered in the paragraph “Other astrocytic tumors”). The clinical course is also likely better for such tumors [35]. Unfortunately, in many such cases, the calibrated score does not reach the ≥ 0.9 cutoff (e.g., between 0.5 and 0.8 for DNT) and the copy-number pattern shows no or no complex aberrations. This may be related to not yet define tumor classes in the spectrum of low-grade gliomas/glioneuronal tumors or a low-tumor cell content of, e.g., a DNT. If the tumor cell content seems high on HE re-evaluation, we would favor the diagnosis of a low-grade glioma, NEC (not elsewhere classified) [30] for such low scoring cases. We would further reanalyze the data of the case once an extended version of the Classifier becomes available.

#### DNA methylation analysis of infiltration zone of diffuse gliomas or highly inflamed tumors

DNA methylation profiling of low-tumor cell content samples is problematic. In the infiltration zone of diffuse gliomas, this will result in a lowering of the scores typically below the threshold of ≥ 0.9. Frequently, the normal brain control tissue methylation classes will have slightly-to-moderately increased scores in such instances (often around 0.3 calibrated score). Highly inflamed tumors may have an elevated score for control tissue “inflammatory tumor microenvironment”. In both instances, we reassess the HE for the approximate tumor content. If the material is, indeed, of low-tumor cell content, we would accept a lower classifier score as sufficient evidence of the respective tumor class. The copy-number pattern will also become less defined with lower amplitude for both gains and losses, and this should be considered, e.g., for assessment of 1p/19q status. In such a case, our diagnosis would read, e.g., “infiltration zone of glioblastoma, IDH wt” (highest score for glioblastoma, IDH wt, and CNV indicative for glioblastoma) or “infiltration zone of diffuse astrocytoma, IDH mut” (highest score A IDH, not the slightest indication for 1p/19q codeletion). If the tumor cell content is even lower and only a notion of a tumor score can be detected (e.g., highest score GBM, IDH wt 0.3, and no copy-number changes), we would consider the material as not diagnostic. In some instances, deep sequencing may then aid in the interpretation if prototypic mutations are detected. Otherwise, we would discuss re-biopsy.

#### Classification of histologically typical glioblastoma or epithelioid glioblastoma as methylation class (anaplastic) pleomorphic xanthoastrocytoma

Morphologically, it seems frequently impossible to draw a clear line between anaplastic PXA, epithelioid GBM, and sometimes even standard GBM. Recently, a case series of epithelioid glioblastomas has been analyzed by DNA methylation profiling, demonstrating that this group can readily be stratified into cases with similarities with pleomorphic xanthoastrocytoma or IDH wt glioblastoma [24]. Cases with BRAF V600E mutation are clearly enriched in the pleomorphic xanthoastrocytoma-like cases but may also be present in a small proportion of IDH wt glioblastomas [24]. In the histologically more anaplastic cases, chromosomal changes are frequently more complex than is typical for standard PXA, indicating that a spectrum of PXA, anaplastic PXA and glioblastoma-like PXA may exist. In case of a ≥ 0.9 classifier score for PXA in a tumor with histological features of glioblastoma, we would perform *BRAF* V600 mutation testing. In case a *BRAF* V600 mutation is detected and the CNV shows a focal deletion of *CDKN2A/B* but no additional complex chromosomal changes we consider the case as PXA (and would grade according to WHO by counting mitotic figures). If no *BRAF* V600 mutation is detected, we consider the case as malignant glioma, NEC. It seems possible that such cases harbor other rare alterations of the MAPK pathway and that they may be more precisely classifiable in the future. An example of one of the latter tumors is shown in Fig. 4c, d.

#### Tumor with glioblastoma or anaplastic astrocytoma histology and methylation profile of a pilocytic astrocytoma or other low-grade tumor (e.g., LGG MYB/MYBL1, or IHG)

This issue cannot be completely resolved to date. In several instances, we have observed this constellation, by far most frequently between GBM and PA (especially in younger children) and several cases of this constellation have been reported previously [26]. In our experience, such tumors seem to be enriched among samples with limited material or artificial tissue alterations such as extensive hemorrhage that may complicate histological assessment. In case of a methylation class of pilocytic astrocytoma and additional clear evidence of a BRAF fusion and no additional complex chromosomal changes (e.g., no CDKN2A/B deletion and no amplifications), we would consider the case a pilocytic astrocytoma if the histology would also fit into the broad spectrum of this entity (allowing, e.g., a moderately higher rate of mitotic figures). If no evidence of a BRAF fusion is present, we would perform additional molecular analyses starting with panel sequencing [41] to gather more information. Some cases may still remain elusive. However, even in those, we would take the Classifier result seriously and would formulate a non-canonical “descriptive” WHO diagnosis such as “glioma with molecular features of pilocytic astrocytoma, NEC”. A retrospective study focusing on pediatric tumors demonstrated that such cases likely follow a clinical course most compatible with that of a low-grade glioma [26]. This was recently further substantiated by the post hoc sub-group analysis of the HERBY Phase II Randomized Trial [31].

### Other astrocytic tumors

**Fig. 5:**
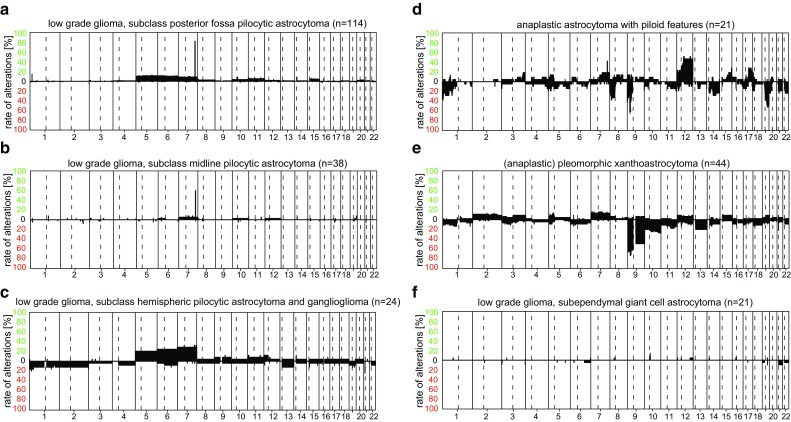
Summary CNV plots of “other astrocytic tumors” including subclasses of pilocytic astrocytoma (**a**–**c**), anaplastic astrocytoma with piloid features (**d**), (anaplastic) pleomorphic xanthoastrocytoma (**e**), and subependymal giant cell astrocytoma (**f**)

**Fig. 6:**
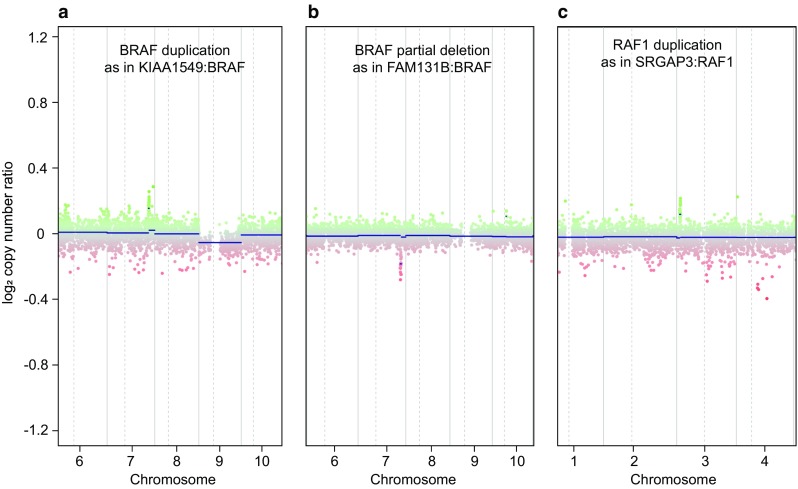
Examples of three pilocytic astrocytomas with different types of MAPK alterations visible on the CNV plots. **a** Shows the by far most frequent focal low-level gain on Chr.7q representing a 7q34 tandem duplication of *BRAF* as part of the *KIAA1549:BRAF* gene fusion; **b** represents a rare fusion event with a focal loss of representing *FAM131B:BRAF* gene fusion; **c** represents a focal gain involving *RAF1* as part of the *SRGAP3:RAF1* gene fusion

Pilocytic astrocytoma is represented by a methylation class family with three subclasses related to tumor location. Of these “low-grade glioma, subclass posterior fossa pilocytic astrocytoma” is a relatively pure pilocytic astrocytoma class, “subclass midline pilocytic astrocytoma” is mostly composed of pilocytic astrocytoma but may also incorporate some cases with appearance of the pilomyxoid variant, whereas “subclass hemispheric pilocytic astrocytoma and ganglioglioma” includes cases of supratentorial pilocytic astrocytoma and cases with additional focal or more widespread ganglioglioma differentiation. Duplication of the BRAF locus is frequent among pilocytic astrocytomas and can be observed as a focal low-level gain indicative for a duplication on Chr.7q (Fig. 6a). The frequency of this duplication varies between the three subclasses (Fig. 5a–c).

A methylation class closely resembling anaplastic pilocytic astrocytoma has recently been described in detail under the name “anaplastic astrocytoma with piloid features” [34] and this term is used throughout this manuscript. The copy-number pattern of these tumors occasionally also demonstrates BRAF duplications but much more frequently harbor CDKN2A/B deletions and further chromosomal changes (Fig. 5d).

Both pleomorphic xanthoastrocytoma and anaplastic pleomorphic xanthoastrocytoma are combined in the “(anaplastic) pleomorphic xanthoastrocytoma” methylation class. The presence of a *BRAF* V600E mutation seen in approximately 70% of pleomorphic xanthoastrocytomas is not a requirement for classification of these tumors into this class. An established but usually underestimated feature of pleomorphic xanthoastrocytoma is homozygous deletion of *CDKN2A* previously reported in 50% [51]. Among cases falling into this methylation, class CDKN2A/B deletions are seen in around 70% of cases (Fig. 5e).

Subependymal giant cell astrocytoma completely matches the “low-grade glioma, subependymal giant cell astrocytoma” methylation class. The Classifier does not differentiate between TSC1- and TSC2-mutated (or TSC wild-type) tumors, and CNV are rare in this group (Fig. 5f).

### Other astrocytic tumors—suggestions for specific scenarios

#### Tumor without classic histological features of PXA but falling into the PXA class

This is observed on a regular basis. A wide spectrum of histologies (from rather monomorphic diffuse gliomas to ganglionic tumors or cases with astroblastoma-like, glioblastoma-like or even ATRT-like features) seem to occasionally share the methylation profile of PXA. Histological reassessment occasionally reveals at least some features of PXA but not in all cases. We generally perform sequencing for BRAF V600 in this setting. If the mutation is present and the tumor, additionally, harbors a CDKN2A/B deletion, we would consider the case as a molecular PXA with unusual histological features. If one or both alterations are missing, we would come to a non-canonical diagnosis of a “glioma with molecular features of PXA, NEC”. For grading, we would still consider the criteria established for pleomorphic xanthoastrocytoma. After having come across a considerable number of such cases, we believe that the morphological spectrum of PXA is likely substantially wider than previously anticipated. Further research including assessment of follow-up data is required to come to a conclusion of the definite classification of such cases, and also to determine the prognostic relevance of the current histological grading.

#### Evaluation of BRAF CNV changes and rare fusions (FAM131B:BRAF and SRGAP3:RAF1)

In our experience, detection of a BRAF duplication in the form of the typical focal low-level gain on Chr.7q representing a 7q34 tandem duplication (Fig. 6a) shows a substantial concordance with other assays for detection of BRAF fusion such as RNA sequencing or other NGS methods. However, there may be cases where the observed indicative CNV cannot be recapitulated by other methods possibly due to unknown fusion partners or other rare genetic constellations. Depending on the tumor cell content, the presence of a BRAF duplication may also be hard to assess. We have introduced the term “…with indication of a BRAF fusion” for the setting when the results are not definite but likely, but this is currently still subjective and we have not been able to define exact criteria. If the typical BRAF duplication is not observed we routinely check for rare alternative fusions, in particular for FAM131B:BRAF and SRGAP3:RAF1 (Fig. 6b, c).

#### Histologically typical pilocytic astrocytoma that falls into the methylation class anaplastic astrocytoma with piloid features

In very rare instances, cases with typical PA histology may be classified as methylation class “anaplastic astrocytoma with piloid features”. If these cases, additionally, harbor a CDKN2A/B deletion and/or immunohistochemical ATRX loss (frequent in “anaplastic astrocytoma with piloid features”, likely never present in PA), we would consider the case as “anaplastic astrocytoma with piloid features”. Reinhardt et al. give further suggestions for the diagnosis of these rare cases [34]. Workup may well involve additional genetic testing (e.g., panel sequencing, [41]). If the case does not show loss of ATRX and no loss of CDKN2A/B, we would be more cautious and would report a “pilocytic astrocytoma with unusual molecular features, NEC”, and would comment the unusual finding of an “anaplastic astrocytoma with piloid features” methylation class profile, which may warrant closer clinical follow-up.

### Ependymal tumors

We find considerable differences between WHO and classifier-diagnosed ependymomas. A comprehensive analysis on methylation-based classification of ependymomas has previously been published [32]. The Classifier reproduces and refines the findings of that study. A feature pronounced in ependymomas is the fact that tumors at different sites seem to represent distinct entities despite a similar histology.

**Fig. 7:**
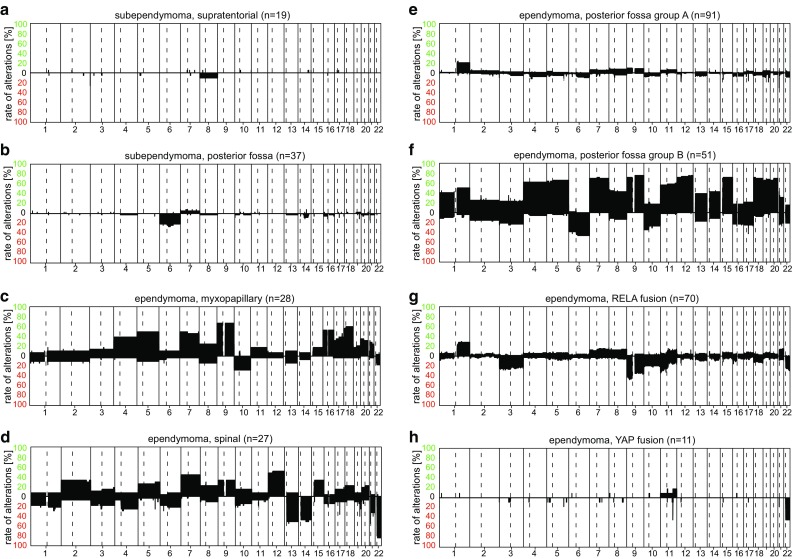
Summary CNV plots of ependymal and subependymal tumors (**a**–**h**)

Subependymoma WHO grade I is generally classified according to location into “subependymoma, supratentorial”, “subependymoma, posterior fossa”, and “subependymoma, spine”. The CNV plots of the three subependymoma methylation classes show infrequent chromosomal gains or losses (Fig. 7a, b; not shown for spinal tumors).

Myxopapillary ependymoma WHO grade I corresponds to the “ependymoma, myxopapillary” methylation class, and “ependymoma, myxopapillary”-assigned tumors exhibit very lively CNV plots in terms of whole chromosome changes. Most tumors exhibit multiple CNV with gain of Chr.5, 7, 9, 16, and 18 being most frequent (Fig. 7c).

The majority of the spinal ependymomas WHO grade II fall within the “ependymoma, spine” methylation class. CNV in “ependymoma, spine” are also frequent, with the dominating alteration being chromosome 22 loss (Fig. 7d). Typically, these tumors are of cervical, less frequently of thoracic and very rarely of lumbar localization.

The WHO variants of papillary, clear cell, and tanycytic ependymoma currently do not form a distinct methylation class but rather fall into the existing methylation classes. Ependymomas of the posterior fossa are clearly distinct from supratentorial ependymomas. Both, infratentorial and supratentorial ependymomas are further sub-grouped with clear clinical impact: Posterior fossa ependymomas form two major classes, “ependymoma, posterior fossa group A” and “ependymoma, posterior fossa group B” [32], which previously have also been identified by transcriptional profiling [54]. The two classes have distinct clinical characteristics and a diagnostic separation of these two classes seems highly relevant. As previously indicated, the CNV profile of “posterior fossa group A” tumors appears rather quiet. The most evident alteration is gain of 1q (Fig. 7e). The clinically more benign tumors in the “ependymoma, posterior fossa group B” methylation class exhibit substantially more CNV (Fig. 7f).

The majority of supratentorial WHO grade II and grade III ependymomas fall into the frequent “ependymoma, RELA fusion” or the exceedingly rare “ependymoma, YAP1 fusion” methylation classes. As the RELA fusion can also be identified by FISH or immunohistochemistry, ependymoma, RELA fusion positive has already been introduced to the 2016 update of the WHO classification. As described in the original publication [33], the cases frequently harbor chromothripsis involving Chr.11q13. This can occasionally be identified in the CNV of these tumors with short sections of gains and losses close to the centromere of Chr.11, sometimes, extending further across Chr.11 [and thus resulting in the slightly jagged line of the summary CNV plot (Fig. 7g)]. The “ependymoma, YAP1 fusion” tumors on average have fewer alterations, with loss close to the *YAP1* locus on Chr.11q and loss of Chr.22 being most frequent (Fig. 7h).

Of note, methylation analysis of the ependymal tumor group provides a completely reshuffled classification system which, in these tumors, obviates the need for further grading as the individual methylation classes already identify patient groups much more homogenous in respect to prognosis than the previous WHO classification and grading was able to provide. Recent data suggest further subdivision of the methylation classes with likely more distinct ependymoma classes to be described in the future.

### Ependymal tumors—suggestions for specific scenarios

#### Histological grade II/III ependymoma classified as subependymoma by methylation profiling

A small proportion of WHO grade II ependymomas (occasionally with tanycytic differentiation) is molecularly classified as subependymoma. Published data suggest that the clinical course of these tumors appears benign, resembling that of subependymoma [32]. If histological reassessment would be compatible also with a low-grade ependymoma, we would diagnose such a case as “subependymoma with unusual histological features”. However, based on the same set of information, a diagnosis such as “histological low-grade ependymoma with methylation profile of subependymoma” would be also acceptable. More caution may be needed when the case just shows an elevated score for subependymoma (but below or even well below the 0.9 cut-off).

#### Tumors with mixed subependymoma and ependymoma features

Along the same lines, all so far analyzed subependymoma/ependymoma composition tumors (*n* = 4) scored as methylation class subependymoma in both tumor areas and we consider the ependymoma parts as a more compact growth pattern of subependymoma. Interestingly, in two of these cases, the CNV plot demonstrated loss of Chr.6 exclusively in the areas with ependymal differentiation with otherwise rather flat CNV plots.

#### Classical spinal ependymoma with a classification of methylation class ependymoma, myxopapillary

In our experience, a considerable fraction of spinal ependymomas with classic ependymoma histology (considered WHO grade II) are classified as “ependymoma, myxopapillary”. These tumors are almost exclusively located in the lumbar region (as would be typical for cases with histology of myxopapillary ependymoma). CNV profiles of these cases are also characteristic for “ependymoma, myxopapillary” and are usually distinct from the CNV patterns of spinal or other ependymoma methylation classes. Histological re-evaluation of these occasionally reveals focal myxoid changes. In two cases, clinical follow-up demonstrated meningeal dissemination which is also better compatible with myxopapillary ependymoma than with spinal grade II ependymoma. We, therefore, consider these cases as “myxopapillary ependymomas with unusual histologic features”. A more conservative approach might designate these tumors as “ependymoma with methylation profile of myxopapillary ependymoma”, although this would neglect the additional and independent data from the copy-number profiles pointing to myxopapillary ependymoma.

#### Histological ependymal tumors with no match to one of the above ependymoma classes

We have repeatedly observed cases with some degree of ependymal morphology that do not match one of the ependymal methylation classes. In particular, among supratentorial tumors, some of these cases get high scores for other existing methylation classes, in particular IDH wt glioblastomas and rarely other classes such as CNS high-grade neuroepithelial tumors with MN1 alteration. In such a setting, we would reconsider if the suggested methylation class would also be compatible with the histology and would reconsider the initial diagnosis.

A second fraction of such cases will have no match to an established class. This likely indicates further classes with histological features of ependymomas that are yet to be defined. In such a constellation, we would comment that the case does not represent one of the defined ependymal classes, but may represent a rare subtype not yet characterized. Diagnostically, we refer to these cases either low- or high-grade “gliomas with ependymal features, NEC”.

### Other gliomas

**Fig. 8:**
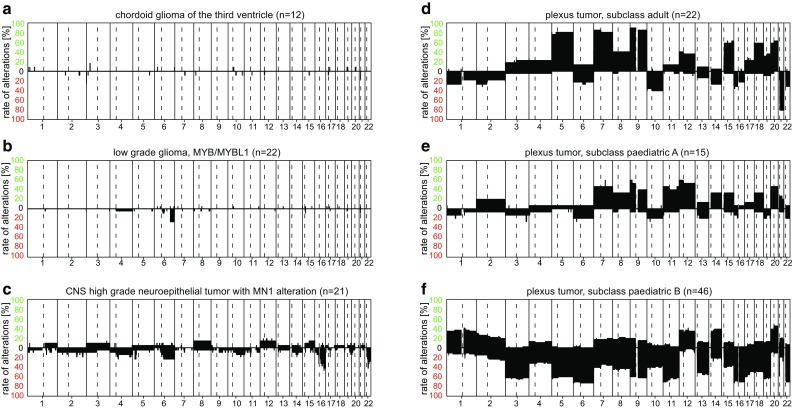
Summary CNV plots of a selection of “other gliomas” (**a**–**c**) and choroid plexus tumors (**d**–**f**)

The chordoid glioma of the third ventricle shows complete overlap with the methylation class “chordoid glioma of the third ventricle” and all cases so far characterized had a flat CNV profile (Fig. 8a). All cases investigated also harbored the recently published PRKCA hotspot mutation [11].

Angiocentric gliomas are typically classified as “low-grade glioma, MYB/MYBL1” methylation class, however, also other low-grade tumors without prominent angiocentric growth pattern occasionally fall into this class. The CNV plot is typically either flat or shows alterations on Chr.6q with a break point classically within the MYB gene in around 30% of cases (Fig. 8b).

Astroblastoma is a WHO diagnosis with a high degree of inter-observer variation. By methylation analysis, a substantial part of astroblastomas are classified as “CNS high-grade neuroepithelial tumor with MN1 alteration” [45]. Likely, the remaining cases are not a single tumor entity but rather harbor genetic alterations that suggest classification as other tumor entities [55]. The summary CNV of “CNS high-grade neuroepithelial tumor with MN1 alteration” is shown in Fig. 8c.

### Other gliomas—suggestions for specific scenarios

#### High-grade glioma (mostly pediatric anaplastic astrocytoma, IDH wt) classified as “low-grade glioma, MYB/MYBL1” methylation class

How to proceed in this rare constellation cannot be fully resolved at date. We have retrospective clinical follow-up of single such patients that indicate a rather benign course of these tumors. If no complex chromosomal changes are observed, we would thus for the time being consider the cases as “proliferating glioma with low-grade molecular features”. We have also observed single cases with additional complex chromosomal changes (e.g., chromothripsis of Chr.6). Here, we would be more cautious to downgrade the tumor and would remain with the diagnosis of a “not further classifiable pediatric glioma with alterations of MYB/MYBL1 and additional complex chromosomal changes”. The clinical course of such cases is currently unknown.

#### Histological astroblastoma classified as other CNS tumor methylation class

Astroblastoma does not have a separate methylation class in the Classifier. Around half of such cases get classified as “CNS high-grade neuroepithelial tumor with MN1 alterations”. The remaining cases either are not classifiable or fall into other methylation classes (e.g., ependymomas, PXA, glioblastoma, and IDH wt). In many cases, additional molecular features of this class can be identified form the CNV plot or by sequencing, e.g., of BRAF V600. If we are able to demonstrate such additional evidence, we would strongly reconsider the diagnosis of astroblastoma and would favor the new class.

### Choroid plexus tumors

The three WHO entities of choroid plexus tumors are combined to a methylation class family. Three subclasses exist, “plexus tumor, subclass adult”, “plexus tumor, subclass pediatric A”, and “plexus tumor, subclass pediatric B” that broadly correspond to the three previously defined methylation clusters [50]. “Plexus tumor, subclass adult” predominantly contains adolescent and adult patients with choroid plexus papilloma and a fraction of approximately 15% of atypical plexus papilloma. “Plexus tumor, subclass pediatric A” is composed half each of atypical choroid plexus papilloma and choroid plexus papilloma, and they mostly occur in pediatric patients. “Plexus tumor, subclass pediatric B” incorporates plexus carcinomas and approximately 30% of tumors diagnosed as atypical plexus papilloma. The tumors show numerous non-complex chromosomal changes with different frequency patterns (Fig. 8d, e, f). Especially, the frequent loss of Chr.21 in “plexus tumor, subclass adult” in comparison to “plexus tumor, subclass pediatric A” is noteworthy. In contrast to the other two classes, “Plexus tumor, subclass pediatric B” is characterized by a higher number of chromosomal losses. The exact relation of these three methylation classes with TP53 somatic/germline status has not yet been resolved.

### Choroid plexus tumors—suggestions for specific scenarios

#### Plexus tumor with benign histology but classification into methylation class “Plexus tumor, subclass pediatric B”

This occurs infrequently. It is more likely that atypical plexus papillomas falling into “Plexus tumor, subclass pediatric B” progress than atypical plexus papillomas falling into the other two subclasses [50]. We have only observed a single diagnostic case of WHO grade I plexus papilloma falling into the “Plexus tumor, subclass pediatric B”, and we were cautious with interpreting this as data on the clinical course of such tumors are not available. In the respective case, we did not change our histological interpretation.

#### Intracranial papillary tumor that may represent a plexus tumor or a metastatic tumor

The DNA methylation class family “plexus tumors” may further be valuable to ascertain that a given tumor is at all related to the choroid plexus (e.g., for metastatic carcinoma vs. plexus carcinoma) and we have observed several cases where plexus tumors could be excluded by methylation profiling in favor of metastatic tumors.

### Neuronal and mixed neuronal-glial tumors

The majority of cases with DNT histology are classified as “low-grade glioma, dysembryoplastic neuroepithelial tumor” methylation class. In our hands, this can be useful for small tumor samples not exhibiting all the histological hallmarks of DNT. With respect to CNV patterns, “low-grade glioma, dysembryoplastic neuroepithelial tumor” is inconspicuous (Supplementary file 1). Interestingly, the proportional distribution of histologically diagnosed DNTs and gangliogliomas shows marked geographical variability across surgical series [49]. The exact reason for this remains unexplained but likely the regionally different interpretation of histological criteria plays a major factor.

**Fig. 9:**
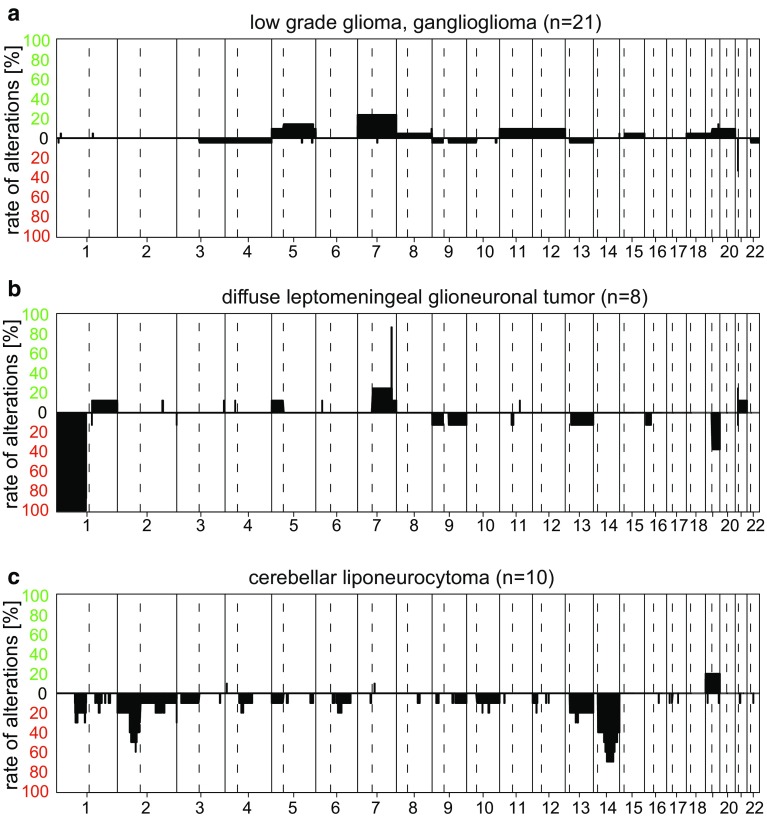
Summary CNV plots of a selection of Neuronal and mixed neuronal–glial tumors (**a**–**c**)

Within the Classifier, “ganglioglioma” is mostly classified either as “low-grade glioma, ganglioglioma” or as the above-described “subclass hemispheric pilocytic astrocytoma and ganglioglioma”. The exact relation of these two groups is currently unclear. Cases with lower tumor cell content frequently are non-classifiable and often get elevated scores for “control tissue, reactive tumor microenvironment”. Whole chromosomal gains are occasionally observed, in particular of Chr.7 (Fig. 9a).

Anaplastic ganglioglioma is not included as a separate methylation class in the Classifier, suggesting that the histological diagnostic criteria currently in use are not sufficient to identify a tumor group with a common methylation pattern. The majority of histologically defined anaplastic gangliogliomas either fall into the PXA or into the GBM, IDH wt classes.

Desmoplastic infantile astrocytoma (DIA) and desmoplastic infantile ganglioglioma (DIG) form the methylation class “low-grade glioma, desmoplastic infantile astrocytoma/ganglioglioma”. CNV profiles show no recurrent chromosomal imbalances (Supplementary file 1), while initial data suggest that BRAF mutations may be relatively common in this group [20].

The WHO entity of rosette forming glioneuronal tumor constitutes the methylation class “low-grade glioma, rosette forming glioneuronal tumor”. Chromosomal changes are rare (Supplementary file 1). Likewise, the recently recognized diffuse leptomeningeal glioneuronal tumor also forms the clearly demarcated “diffuse leptomeningeal glioneuronal tumor” methylation class. The copy-number pattern of these tumors is rather specific and frequently demonstrates focal 7q34 gain indicating BRAF fusion in combination with 1p deletion and occasionally additional 19q deletion (Fig. 9b). Very recently, a series of molecularly defined diffuse leptomeningeal glioneuronal tumors demonstrated two sub-groups with distinct clinical and genetic features among these tumors [8]. These sub-groups are currently not separately detected by the Classifier but are expected to be included in a future update.

Central neurocytoma and atypical central neurocytoma are classified as “central neurocytoma” methylation class. Separation between these two is not possible by methylation analysis. In contrast, the few extraventricular neurocytomas which we had the chance to analyze were either non-classifiable or fell into various methylation classes but never to “central neurocytoma”. We take this a caveat for assuming that these tumors are simply extraventricular manifestations of central neurocytoma. Further research is needed to define the identity of these tumors. CNV profiles show no recurrent chromosomal imbalances (Supplementary file 1).

Cerebellar liponeurocytomas again form a unique methylation class termed “cerebellar liponeurocytoma”. The CNV profile indicates recurrent chromosomal losses especially of regions on Chr.2p and Chr.14 (Fig. 9c).

In particular, familial paraganglioma may carry germline SDH mutations associated with a CpG island methylator phenotype (CIMP) [27]. This group is currently not recognized by the Classifier due to a lack of numbers, but preliminary data reveal clear differences in their methylation pattern. Paraganglioma without SDH mutations constitute the vast majority of sporadic tumors form the “paraganglioma, spinal non-CIMP” methylation class. CNV profiles show no recurrent chromosomal imbalances (Supplementary file 1).

Gangliocytoma, dysplastic cerebellar gangliocytoma (Lhermitte–Duclos disease) and papillary glioneuronal tumors currently are not recognized by methylation-based classification, as there were too few cases to build up such classes.

### Neuronal and mixed neuronal-glial tumors—suggestions for specific scenarios

#### Histological low-grade glioma/diffuse glioma without ganglion cell differentiation classified as ganglioglioma

**Fig. 10:**
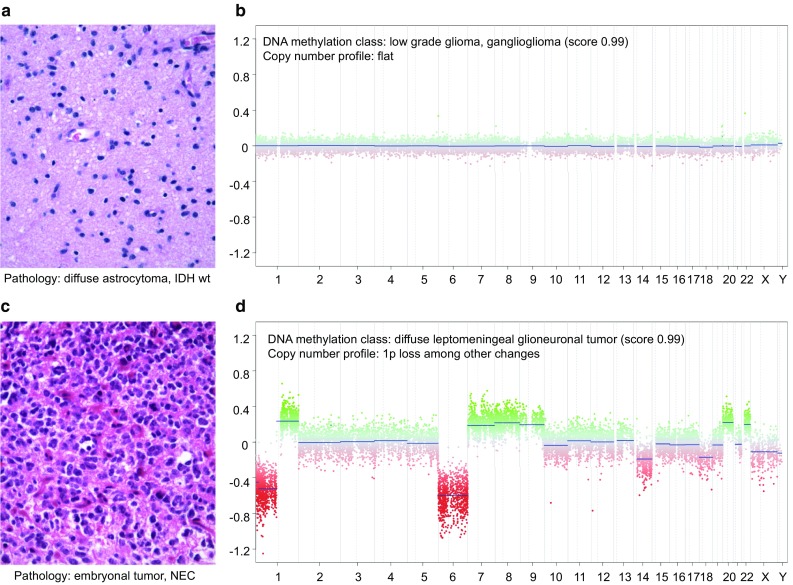
Example of cases with conflicting pathological and molecular results. **a** H&E of a tumor composed of relatively monomorphous, indistinct cells pathologically interpreted as diffuse astrocytoma, IDH wt. **b** By methylation profiling, the tumor corresponds to a ganglioglioma. The copy-number profile is flat and would also fit to the latter (but is also not specific for this). BRAF sequencing revealed a V600E mutation. The case was interpreted as ganglioglioma in the integrated diagnosis. **c** H&E of a supratentorial embryonal tumor, NEC with a Ki67 proliferation fraction of approximately 20% (not shown) that by methylation profiling, **d** corresponds to a diffuse leptomeningeal glioneuronal tumor. A BRAF duplication was not present. Anaplastic transition of diffuse leptomeningeal glioneuronal tumor has been documented [38]. Likely, the histological spectrum of tumors with the molecular profile of diffuse leptomeningeal glioneuronal tumors is even wider [8] and the way to best categorize such cases remains to be established

Repeatedly, we have observed cases with a high score for “low-grade glioma, ganglioglioma” methylation class but without clear histologic evidence for ganglionic differentiation. Many of these tumors are composed of relatively monomorphous, indistinct cells (not clearly glial, not clearly ganglionic). Genetic testing has revealed BRAF V600E mutation in some of these; CDKN2A is not deleted. Other immuno-markers like CD34 were mostly negative in these cases. Because of the strong molecular data and due to the fact that cells of indistinct differentiation are frequent in ganglioglioma [21], we consider these cases as ganglioglioma, even though fully developed ganglionic cells are missing (Fig. 10a, b). Alternatively, these tumors could be designated as “low-grade glioma with methylation profile of ganglioglioma”. We expect that such issues will be resolved in the future; however, our current feeling is that the DNA methylation-based suggestion may be more reproducible. Importantly, complex chromosomal changes should not be present; otherwise, we would be more cautious in our interpretation.

#### Unexpected high score for DIG/DIA

The Classifier score of DIG/DIA has to be taken with caution as we have realized that other tissues with desmoplastic reaction (e.g., reactive fibroblastic proliferation close to a tumor) may get a high score for this class and, in some instances, even above the 0.9 threshold. Currently we would consider an elevated score for a tumor not occurring in the typical clinical setting of DIG/DIA (infant, hemispheric tumor) as not valid.

#### DNA methylation class “diffuse leptomeningeal glioneuronal tumor” but tumor has different histological appearance

We observe cases scored as methylation class “diffuse leptomeningeal glioneuronal tumor” more frequently than anticipated. We have also observed that occasionally cases not presenting with the typical clinical and histological appearance may get a high classification score for this class. This is also covered in a recent case series of molecularly defined diffuse leptomeningeal glioneuronal tumors [8]. These tumors may histologically present as extraventricular neurocytomas, embryonal tumors, NEC, or as pilocytic astrocytomas, and we have observed them in spinal, posterior fossa, and supratentorial locations. Figure 10c, d shows an example of a supratentorial case with embryonal tumor histology currently recurrence-free, since the initial diagnosis of an embryonal tumor, NEC (back then called PNET) 5 years ago. Further data on this new WHO entity are needed to draw conclusions of how such cases should best be classified in the future.

#### Elevated “dysembryoplastic neuroepithelial tumor” methylation class score (but below threshold of 0.9) in low-grade glioma/glioneuronal tumor and non-tumorous tissues

In various non-classifiable cases, elevated scores for “dysembryoplastic neuroepithelial tumor” are observed, in most cases clearly below the threshold of 0.9. These scores should be taken with caution and should not automatically be interpreted as “dysembryoplastic neuroepithelial tumor” with low-tumor content, as the “dysembryoplastic neuroepithelial tumor” methylation class seems to be prone to “hijack” scores form other classes and non-classifiable classes. For example, we have observed that focal cortical dysplasias also get Classifier scores between 0.3 and 0.8 for “dysembryoplastic neuroepithelial tumor”. The reason for this is unclear.

### Tumors of the pineal region

Pineocytoma appears to exhibit a methylation profile very close to that of normal pineal gland, therefore, not allowing the construction of a distinct methylation class. Pineocytomas are thus mostly non-classifiable or may even be classified as “control tissue, pineal gland” methylation class.

**Fig. 11:**
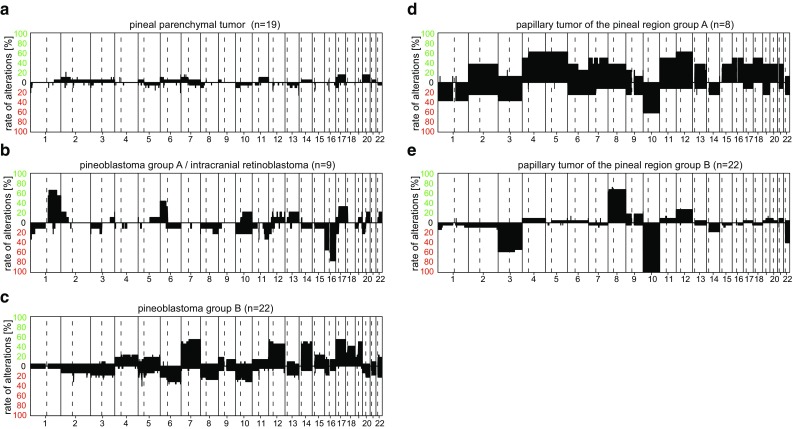
Summary CNV plots of pineal region tumors (**a**–**e**)

Pineal parenchymal tumor of intermediate differentiation is represented by the methylation class “Pineal parenchymal tumor”. CNV patterns in this group are not characteristic (Fig. 11a). This methylation class also contains some cases histologically diagnosed as pineoblastoma likely reflecting the continuous gradient of morphology between these lesions.

Two separate methylation classes are defined for pineoblastoma: the more frequent “pineoblastoma group B” and the rare “pineoblastoma group A/intracranial retinoblastoma”. “pineoblastoma group B” also contains some pineal parenchymal tumors of intermediate differentiation, while “pineoblastoma group A/intracranial retinoblastoma” contains some tumors diagnosed as retinoblastoma. We assume these retinoblastomas to be genetically related to the so-called trilateral retinoblastomas detected in approximately 5% of patients with hereditary retinoblastoma who, in addition to bilateral ocular tumors, develop a pineal tumor with morphological characteristics of pineoblastoma [7]. Typical CNV in “pineoblastoma group A/intracranial retinoblastoma” are gain of Chr.1q, gain of 6p and loss of 16q (Fig. 11b), while “pineoblastoma group B” shows frequent gain of Chr.7, 12, 14, 17, and 19q and a characteristic focal loss on Chr.5p (Fig. 11c).

Two methylation classes (A and B) are defined for papillary tumor of the pineal region. “Papillary tumor of the pineal region group A” exhibits a high number of CNV with gains of 4, 5, 11, and 12 and loss of Chr.10 being most frequent (Fig. 11d). The CNV profile of “papillary tumor of the pineal region group B” also shows characteristic changes with all cases showing loss of Chr.10 among other changes (Fig. 11e). A more aggressive clinical course has been suggested for “papillary tumor of the pineal region group B”, but data on this is still preliminary [13].

### Tumors of the pineal region—suggestions for specific scenarios

#### Papillary tumor of the pineal region not located in the pineal region

**Fig. 12:**
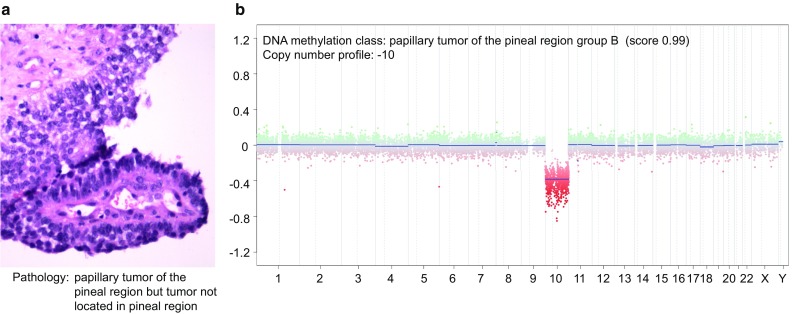
Example of a case with conflicting pathological and molecular results. H&E of tumor of a pediatric patient showing high resemblance to a papillary tumor of the pineal region, but that is located in the 4th ventricle and had no contact to the pineal region. The DNA methylation profile also corresponded to papillary tumor of the pineal region and the CNV profile would also be compatible with this. It is not clear whether this represents an ectopic papillary tumor of the pineal region or a distinct so far not defined tumor class

This is a rare finding. We have observed single cases located in the 4th ventricle that was well compatible with papillary tumor of the pineal region both by histology and DNA methylation profiling (Fig. 12a, b) and also harboring the characteristic Chr.10 loss. It is not clear whether this represents an ectopic papillary tumor of the pineal region or a distinct so far not defined tumor class.

### Embryonal tumors

**Fig. 13:**
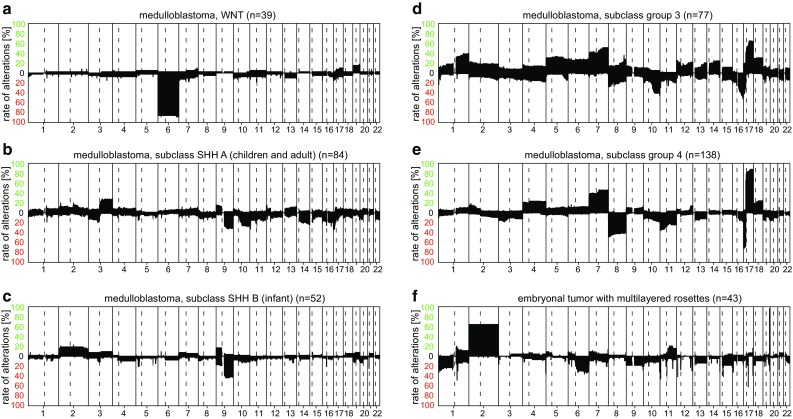
Summary CNV plots of medulloblastoma sub-groups (**a**–**e**) and embryonal tumor with multilayered rosettes (**f**)

WNT-activated medulloblastomas constitute the “medulloblastoma, WNT” methylation class. These tumors typically exhibit loss of Chr.6 with, otherwise, only few copy-number alterations (Fig. 13a). Due to differences in the DNA methylation profile, SHH-activated medulloblastoma have been divided into the two subclasses “SHH A (children and adult)” and “SHH B (infant)” together forming a methylation class family. The two methylation subclasses are likely related to the age dependent spectrum of genetic alterations found in SHH medulloblastoma [22]. Both SHH medulloblastoma subclasses do not have highly characteristic CNV (Fig. 13b, c), but are both moderately enriched for cases with loss of 9q. Group 3 and group 4 medulloblastomas also form a methylation class family consisting of “medulloblastoma, subclass group 3” and “medulloblastoma, subclass group 4”. Tumors in both methylation classes frequently show an isochromosome 17q, approximately 60% in group 3 and 80% in group 4. Group 3 medulloblastoma tend to have a higher number of alterations, most frequently gain of 1q and, 7 and loss of 10q (Fig. 13d). Besides isochromosome 17q, most abundant in group 4 medulloblastomas is gain of 7p and loss of 8 (Fig. 13e). The frequency of amplifications of MYCN on Chr.2p or MYC on Chr.8q is not readily extractable from the summary CNV.

Embryonal tumor with multilayered rosettes, C19MC-altered is another genetically defined WHO entity. It completely matches the methylation class “embryonal tumor with multilayered rosettes”. By definition, the CNV plot shows a focal amplification of 19q13. Furthermore, Chr.2 gain is seen in approximately 60% of these tumors (Fig. 13f). The very few cases that we have so far observed of the exceedingly rare non-19q13 amplified embryonal tumors with multilayered rosettes also fall into this group. The distinct methylation class of intraocular medulloepithelioma has not yet been included in the Classifier [17, 25, 40].

**Fig. 14:**
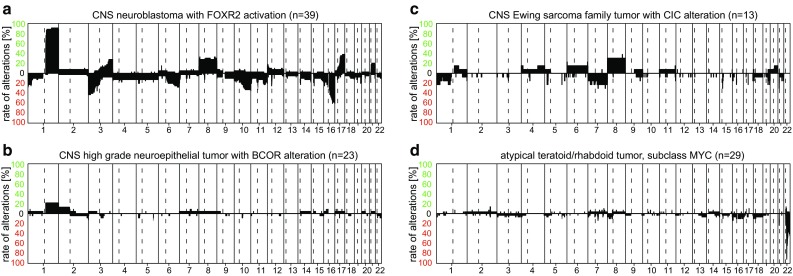
Summary CNV plots of a selection of embryonal tumors (**a**–**d**)

CNS neuroblastoma is represented by the methylation class “CNS neuroblastoma with FOXR2 activation”. These tumors show a characteristic gain of Chr.1p and frequently an additional loss of Chr.16q (Fig. 14a). These tumors have recently been defined in more detail [45].

The exceedingly rare class of CNS ganglioneuroblastomas is currently not represented as a distinct class in the Classifier.

CNS embryonal tumor, NOS, has been extensively studied and some of the cases could be allotted to newly defined molecular classes [45]. Besides “CNS neuroblastoma with FOXR2 activation” the three classes of “CNS high-grade neuroepithelial tumor with BCOR alteration”, “CNS Ewing sarcoma family tumor with CIC alteration”, and the previously mentioned “CNS high-grade neuroepithelial tumor with MN1 alteration” may frequently present with primitive embryonal histology. The classes show different CNV profiles (Figs. 8c, 14b, c) and additional defining molecular changes have been described [45]. CNV profiles of “CNS Ewing sarcoma family tumor with CIC alteration” and “CNS high-grade neuroepithelial tumor with BCOR alteration” exhibit no characteristic features, whereas “CNS high-grade neuroepithelial tumor with MN1 alteration” very frequently shows extensive alterations on Chr.X reminiscent of chromothripsis (Chr.X not shown in the summary CNV plots).

Atypical teratoid/rhabdoid tumor (ATRT) is represented by three related methylation classes: “ATRT, MYC”, “ATRT, SHH”, and “ATRT, TYR” forming the methylation class family ATRT [18]. The tumors in all three methylation classes are characterized by loss of INI1 protein (*SMARCB1*). However, the gross alteration patterns of Chr.22 vary, with whole chromosome loss in almost all “ATRT, TYR”, either whole chromosome or focal loss in almost all “ATRT, MYC” and only 50% of cases with whole chromosome loss (and rather more inactivating point mutations) in “ATRT, SHH” (Fig. 14d for ATRT MYC, for SHH and TYR refer to Supplementary file 1). So far, we have not had the chance to investigate tumors with the rare alternative mutation in *SMARCA4*.

### Embryonal tumors—suggestions for specific scenarios

#### Histology compatible with medulloblastoma but tumor non-classifiable by DNA methylation profiling

Medulloblastomas are represented by a high number of reference cases and are among the best-defined groups in the Classifier. Therefore, a failed classification should prompt consideration of other tumor classes not included in the Classifier such as non-defined embryonal tumors or metastases. A constellation that may lower the score of an SHH medulloblastoma below the threshold of 0.9 is the rare case of an IDH1 R132 mutant SHH medulloblastomas (score expected in the range of ~ 0.4 to 0.7), so testing for IDH point mutation may be of help in some instances [9]. Furthermore, we have currently observed too few cases of some rare histological subtypes like melanotic medulloblastomas; therefore, we are currently not certain how these behave in the Classifier. If none of these issues (IDH, or rare histological subtype) is present to explain a non-classifiable medulloblastoma, we would consider the case as “embryonal tumor, NEC”.

#### Medulloblastoma recurrence in field of irradiation

We have observed several glioblastoma-like tumors operated as suspected local recurrences in the setting of previously irradiated medulloblastomas [56]. These may have a relatively primitive morphology and could also be mistaken for a medulloblastoma recurrence. Therefore, we generally would perform methylation profiling of such cases. Recurrent medulloblastomas maintain their characteristic methylation profile.

#### Tumor is classifiable as group 3/4 family medulloblastoma, but separation between group 3 and 4 is not possible (cutoff of 0.5 for family member sub-classification not reached)

Such cases may rarely occur and we would remain with the family group of group 3/4 medulloblastoma.

#### SHH medulloblastoma in a child or adolescent with additional chromothripsis or unusual complex chromosomal changes and possibly immunohistochemical TP53 accumulation

This constellation should prompt consideration of a Li–Fraumeni syndrome-associated medulloblastoma even if there is no family history of such a syndrome. Because of the relevance to treatment, we would report our suspicion and would advise genetic counselling. We have observed more Li–Fraumeni syndrome cases than previously anticipated and highly recommend the testing of TP53 for all cases with SHH medulloblastoma.

#### Relevance of SHH medulloblastoma sub-classification

Among SHH medulloblastomas, the various activating alterations of the SHH pathway are not evenly distributed [22]. Therefore, the methylation subclass may be helpful to guide a stepwise approach for further molecular analyses if the definite SHH pathway alteration is sought.

#### Medulloblastoma has focal gain of either MYC/MYCN but below what is expected for amplification

The bulk CNV analysis of tumors may mask subclonal events such as subclonal amplifications. We have observed that, in a subclonal amplification, the amplitude of the “gain” is reduced but frequently still visible as a small focal elevation of the signal, e.g., of MYCN. Thus, even a small amplitude focal gain of, e.g., MYCN should be considered of potential relevance, and should be followed up with other methods that allow a subclonal analysis (such as FISH). “Low-level amplifications” are also the most frequently visible indication for DNA cross contamination between samples (see above and Fig. 1d, e), so this should also be considered.

#### Cases with non-cerebellar location but increased medulloblastoma classifier score

We have observed single of such cases. Typically, the location is close to the pineal gland and classification scores are below 0.9 for group 3 medulloblastoma. Possibly, these cases represent a rare not currently defined class. Further cases need to be collected to draw conclusions.

#### Embryonal tumor not classifying to any methylation class

These cases still occur quite frequently among embryonal tumors and we would report these as “embryonal tumors, NEC” [30]. Such tumors may likely represent not yet defined tumor entities, as proposed for one rare tumor class with recurrent amplification of Chr.6q24.2 [6]. Some of these tumors may also be associated with hereditary predisposition syndromes as the Classifier may not yet reliably classify the full spectrum of such tumors.

### Tumors of the cranial and paraspinal nerves

**Fig. 15:**
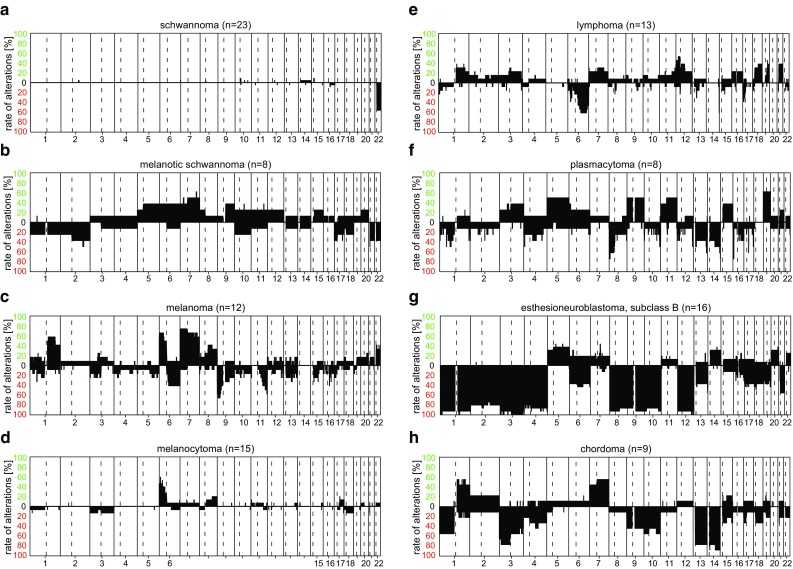
Summary CNV plots of a selection of tumors of the cranial and paraspinal nerves (**a**–**b**), melanocytic tumors (**c**–**d**), large B-cell lymphoma (**e**), and plasmacytoma (**f**), and selected other intracranial tumors (**g**–**h**)

Tumors listed in this WHO chapter are not thoroughly covered by the CNS tumor Classifier. Schwannoma and cellular schwannoma are combined in the methylation class “schwannoma”. This group exhibits localization-dependent sub-groups that are not included in the Classifier [39]. The most frequent CNV in “schwannoma” is loss of 22q seen in approximately 60% of tumors (Fig. 15a). “Melanotic schwannoma” represents a separate methylation class and may be particularly helpful in the differential diagnosis of other melanocytic lesions. Members of the methylation class “melanotic schwannoma” exhibit loss of Chr.22 in approximately 50% and equally frequently loss of Chr.21 in addition, with a wide spectrum of other CNV observed at lower frequency (Fig. 15b).

Malignant peripheral nerve sheath tumors have been shown to form distinct methylation classes among peripheral nerve sheath tumors [39]. However, these classes have not been included in the CNS tumor classifier, because a sarcoma classifier containing these tumors is currently under development.

### Meningiomas

Meningiomas, independent of grade and subtype are represented by the broad methylation class “meningioma” in the Classifier. However, recent work shows that meningioma can be sub-grouped into three benign (MC ben-1, 2, 3): two intermediate (MC int-A, B) and one malignant (MC mal) methylation classes. The progression-free survival of benign, intermediate, and malignant MCs is highly distinct. In fact, the power for predicting recurrence of these six methylation sub-groups is higher than that of WHO grading [42]. These epigenetic sub-groups, their mutational characteristics, CNV, and the association with histology and outcome have been previously reported [42]: Cases of MC ben-1 have typically no aberrations besides 22q deletion and *NF2* mutation. They mostly display fibroblastic or transitional histology. MC ben-2 is characterized by flat CNPs and an enrichment for *KLF4*/*TRAF7*, *AKT1*/*TRAF7*, and SMO mutations. Histology is often of meningothelial (*AKT1*, *SMO*) or secretory (*KLF4*/*TRAF7*) subtype. If needed, detection of the exact *AKT1* or *SMO* mutation holds potential for targeted therapy in case of a biologically low grade but not resectable meningioma. MC ben-3 is the only sub-group harboring many gains of chromosomes, with or without 22q loss and NF2 mutation. Most angiomatous, metaplastic, and microcystic cases fall into this sub-group. The intermediate and malignant MCs mostly have 22q deletion and *NF2* mutations, with the number of losses of whole chromosomes (10, 14) or chromosomal arms (1p) increasing with malignancy. These groups, particularly MC mal, can also carry *TERT* promoter mutations and show *CDKN2A* deletion. However, a flat copy-number profile does not necessarily indicate a low-grade biology. For example, cases with *BAP1* mutations can have no or only focal chromosomal alterations but still be identified as MC mal, in line with their aggressive behavior.

After confirmation of the diagnosis “meningioma” by the CNS tumor classifier, the data can subsequently be analyzed by the meningioma classifier (also available at https://www.molecularneuropathology.org/mnp) for the identification of the respective sub-group.

While evidently malignant meningioma can be readily identified by histology, the power of the epigenetic meningioma sub-groups is the detection of aggressive cases despite inconspicuous histology and a finer separation between benign cases and cases at elevated risk of recurrence than the WHO classification provides. Typical settings in which the epigenetic meningioma profiling is applied, therefore, include cases in which a low-grade histology does not match the clinical impression or if histology is ambiguous between WHO grade I and II.

### Mesenchymal, non-meningothelial tumors

This tumor group contains tumors which will be incorporated in a separate sarcoma classifier. The CNS tumor classifier contains the methylation classes “hemangioblastoma”, “hemangiopericytoma” “Ewing sarcoma”, and “Ewing sarcoma family tumors with CIC alterations” (the latter already briefly discussed in “embryonal tumors”). The summary CNV plots for these classes can be found in the Supplementary file 1. A more extensive methylation-based classifier for soft tissue and bone tumors is currently under development (unpublished data).

### Melanocytic tumors

The methylation class “melanoma” consists of melanoma brain metastases of dermal tumors. Primary malignant melanomas of the CNS were not available in sufficient numbers to generate a methylation class. The methylation class “melanocytoma” is clearly separate from both dermal melanoma metastases and melanotic schwannoma. Tumors of the methylation class “melanoma” exhibit many CNV with 1q gain, 6p gain, 6q loss, 7 gain, 9p loss, and 11q loss being most frequent (Fig. 15c). CNV in melanocytoma are less frequent, with a gain of 6p being the most common event (Fig. 15d). In our experience, methylation-based analysis is helpful in sharpening the distinction between dermal melanoma metastasis and melanocytoma, and between melanocytoma and melanotic schwannoma [19]. It should be kept in mind that uveal melanoma and melanocytoma share both methylation signature and specific mutations, most frequently GNAQ and GNA11 [12].

### Large B-cell lymphoma and plasmacytoma

Hematopoietic tumors are represented by the methylation classes “lymphoma” and “plasmacytoma”. The “lymphoma” methylation class was formed exclusively by “diffuse large B-cell lymphoma”, but it was not tested if other lymphomas are also categorized into this class (hence, the broad name of “lymphoma” was preferred for this class). These tumors show multiple CNV, with loss of 6q and gain of 12p seen in more than half of the tumors (Fig. 15e). The methylation class “plasmacytoma” consists of skull and spinal lesions of plasmacytoma. They are characterized by multiple CNV changes (Fig. 15f).

### Histiocytic tumors and germ cell tumors

Both of these tumor classes have not yet been included in the Classifier, but will be added in a forthcoming update.

### Tumors of the sellar region

Adamantinomatous craniopharyngiomas are represented in methylation class “craniopharyngioma, adamantinomatous”, while papillary craniopharyngiomas are represented by “craniopharyngioma, papillary”. The corresponding CNV profiles are inconspicuous (Supplementary file 1), but these tumors are characterized by either *CTNNB1* or *BRAF* V600E mutations, respectively [3, 43]. Granular cell tumor, pituicytoma, and spindle cell oncocytoma all exhibit very similar methylation patterns and group together forming the “pituicytoma/granular cell tumor/spindle cell oncocytoma” methylation class. No frequently recurring chromosomal alterations are observed (Supplementary file 1).

### Other intracranial tumors

Several intracranial primary tumors with relevance to the neuropathologist are included in other books of the WHO series. Of these not included among the CNS tumors, olfactory neuroblastoma/esthesioneuroblastoma, retinoblastoma, chordoma, and several subtypes of pituitary adenoma are included in the current version of the classifier.

Olfactory neuroblastoma/esthesioneuroblastoma forms two closely related methylation classes: “esthesioneuroblastoma, subclass A” and “esthesioneuroblastoma, subclass B”. The corresponding CNV profiles are also very similar with both showing a very high incidence of losses of whole Chr.1–4, 8–10, and 12 (shown for subclass B in Fig. 15g, subclass A in Supplementary file 1). Interestingly, in a recent series of 66 institutionally diagnosed olfactory neuroblastomas/esthesioneuroblastomas, only 42 (64%) of cases were classified as DNA methylation esthesioneuroblastoma, subclass A or B [5]. Analysis of the remaining cases demonstrated a mix of cases either with close relation to IDH2-mutant sinonasal carcinomas (11%), other sinonasal carcinomas (20%), or to a possible new class of sinonasal tumors with high global methylation (6%). Thus, caution may be required with the interpretation of the diagnosis of an institutionally diagnosed olfactory neuroblastoma/esthesioneuroblastoma. This study further indicated that the distinction of the two separate subclasses esthesioneuroblastoma A and B may be rather unstable, and thus, it is possible that the two subclasses will be reunited in future versions of the Classifier.

Retinoblastoma is also included as a small group in the CNS tumor Classifier. CNV shows several complex copy-number changes that have not yet been analyzed in detail and that may require further validation (Supplementary file 1).

Chordomas all cluster together forming the “chordoma” methylation class. These tumors frequently exhibit chromosomal gains of 1q, 7 and losses of 1p, 3, 9, 10, 13q, and 14q (Fig. 15h).

The pituitary adenomas form separate methylation classes reflecting the expression of anterior lobe hormones. The CNV profiles of the various subtypes are given in Supplementary file 1. Interestingly, adenomas with growth hormone (somatotropin) expression separate into three different methylation classes: two of which relate to the densely and one to sparsely granulated adenoma. More data on the relevance of these sub-groups are required.

With the exception of melanoma metastasis (see above), other types of solid metastases are currently not included in the Classifier. When run through the Classifier, we have observed that various classes may be scored with low scores, most frequently “Plexus tumor, subclass pediatric B”. Typically, these scores are below 0.5, but further testing is required with a broader spectrum of metastases to exclude that some tumors may also obtain higher scores. A low score for Plexus tumor, subclass pediatric B in an unexpected setting should, therefore, be treated with caution.

### Indications for methylation-based classification from a clinical perspective

Machine learning-based classification is a powerful tool for pathologists to assist in diagnosing CNS tumors. Increasing diagnostic precision is accompanied by more reliable prognostic evaluation which may be appreciated by patients and clinicians. Precise diagnosis is mandatory if subsequent treatment is affected. Some diagnoses per se should immediately be questioned, such as the diagnosis of an IDH wt astrocytoma. We find this a frequent diagnostic problem in stereotactic biopsies, where likely the limited size of the specimen precludes the detection of pathognomonic morphological hallmarks. However, this diagnosis should not be accepted by anyone involved in postoperative therapy of glioma patients, because it comprises glioblastomas and occasionally lower grade astrocytomas in need of very different treatments.

Physicians treating pediatric patients have a high demand for diagnostic refinement. Subtyping of medulloblastoma into four groups with different prognosis has already prompted adaptation of treatment regimens (e.g., in the PNET5 study; NCT02066220). This extends to a demand for sequence data on specific genes for targeted therapy. The *TP53* status is of relevance, because an underestimated proportion of medulloblastoma patients carry *TP53* germline mutations, putting these patients at very high risk of developing secondary neoplasia upon irradiation or DNA-damaging chemotherapy. Ependymoma diagnosed according to WHO poses an inherent problem as to the relevance of grading. The division of posterior fossa ependymoma in group A and B as well as supratentorial ependymoma in RELA and YAP likely provides a more reproducible categorization than the current WHO grading. While ependymoma, PF A is associated with worse prognosis than ependymoma, PF B, the prognostic power of methylation-based classification in supratentorial ependymomas is not as clear. It has been noted that the outcome of ependymoma, RELA is less favorable than that of ependymoma, YAP [32]; however, this may be in need of further prospective study. In our experience, the classification and grading of glial tumors of children is likely more error prone than that of adult patients, further advocating additional testing for the entirety of this population. Very recent reports have provided convincing evidence for novel tumor entities not represented in the WHO classification. While the current experience with treatment is limited due to low numbers and short observation time, it is reasonable to expect different treatment regimens for these tumors in the future.

Studies designed for testing new drugs or treatment modalities highly depend on biological homogeneity of the tumors of patients included. In many instances, the natural course of tumors exhibiting morphological similarity, but genetically representing different entities can have more of an impact on progression-free or overall survival than the therapeutic effect of the tested treatment regimen. This attaches importance to maximal precision of diagnosis, at the latest upon entry into a clinical trial.

As therapy develops and becomes more expensive, an increase of diagnostic precision will result in a better return on this investment at relatively low costs for testing. Many therapies carry inherent danger of secondary damage. Precise diagnostics will also prevent overtreatment of patients whose tumors may naturally display a more favorable prognosis. Novel findings in meningioma, for example, may point this way. Mainly depending on mitotic count, meningiomas are currently divided into WHO grade I, atypical grade II, and anaplastic WHO grade III. Approximately 8% of meningiomas are considered atypical and many of these patients receive postoperative treatment. Classification into distinct methylation classes appears to better prognosticate recurrence-free survival than WHO grading can accomplish [42]. This allows identification of patients receiving the diagnosis of meningioma WHO grade I with increased risk for recurrence, as well as patients receiving the diagnosis of atypical meningioma WHO grade II who, in fact, have no increased risk for recurrence. This equally holds true for low-grade gliomas in children misdiagnosed as high-grade gliomas. In this setting, an increase in diagnostic resources could be highly economic and beneficial to such patient groups.

### Heidelberg experience and approach

In our multidisciplinary setting for diagnosing and treating CNS tumor patients, we have developed an approach for employing methylation-based classification in typical diagnostic and clinical settings.

#### Tumor of unclear histological diagnosis

This experience is a frequent event in diagnostic routine with tumors with obviously unusual features in around 1/30 to 1/50 cases in our hands. In the past, most attempts aimed at finding a best fit with a WHO entity for such tumors. However, recent considerations by the WHO advocate forming more homogenous diagnostic groups and accept tumors that may evade classification by such clearly defined parameters. We, therefore, perform methylation analysis for all tumors not obviously in line with the current WHO classification guidelines. A sizeable portion of these tumors could be allotted to defined methylation classes by the Classifier. However, the fraction of tumors not recognized by the Classifier is much higher among this set than that encountered in serial analysis of all CNS tumors submitted for diagnosis. Interestingly, we find an over proportional fraction of unclassifiable CNS tumors to be associated with different hereditary tumor syndromes, and/or to be diagnosed in childhood.

#### Tumors with unexpected clinical course

The typical example for this setting is a tumor recurrence after a long event-free interval of a tumor initially diagnosed as highly malignant. A considerable portion of recurrent glioblastomas with long-term survival upon methylation analysis turned out as pleomorphic xanthoastrocytomas or glioblastoma IDH-mutated, or even low-grade glioma. Vice versa, several tumors with an initially benign evaluation turned out malignant and upon methylation analysis could have been identified as such already at the first surgery.

#### All pediatric CNS tumors

There are several reasons for examining all pediatric CNS tumors by methylation analysis: For medulloblastomas and ependymomas, therapy decisions in Heidelberg are often more heavily influenced by methylation class rather than WHO diagnosis. In general, the error rate in the diagnosis of pediatric CNS tumors is likely higher due to the increased variety of entities observed. Pediatric CNS tumors have a higher fraction of specimens not matching established methylation classes. This indicates an increased demand in the identification of possibly novel entities. Pediatric CNS tumors are also rare overall, which means a manageable increase on budget strain given the added value obtained. However, we fully agree that an adamantinous craniopharyngioma with a CTNBB1 mutation is not in need of additional DNA methylation analysis.

#### Tumors from adult patients in need for additional subclassifications

In the adult age group, this encompasses patients with the morphological aspect of astrocytoma but lacking *IDH* mutations. In most instances, these patients turn out to have glioblastoma based on methylation class and CNV patterns. Another typical tumor to be included is morphologic astrocytoma with *IDH* mutation and maintaining nuclear ATRX expression. The latter group frequently turns out to be oligodendroglioma with evident 1p/19q codeletion. While this decision may also be made based on the FISH analysis, we find evaluation of the CNV pattern much more reliable. We regularly also submit low-grade gliomas and glioneuronal tumors to methylation analysis. We find this very helpful in distinguishing between ganglioglioma, pilocytic astrocytoma, and pleomorphic xanthoastrocytoma.

#### Infiltrating tumors with high content of residual brain tissue

Tissue from bordering areas of diffuse gliomas frequently evades correct WHO classification. Especially, in diffuse glial tumors of low cellularity but unusually high proliferation, methylation analysis was repeatedly helpful in identifying molecular features of glioblastoma. Frequently, these cases did not reach the 0.9 cutoff required for the regular classification; however, CNV analysis proved quite robust, because gain of 7 and loss of 10 can readily be detected on a high background of normal tissue. Amplifications such as *EGFR* or *MDM2* and homozygous deletions of *CDKN2A* are even more robust when present in the tumor. Importantly, we find such tumors to carry multiple chromosomal losses or gains. On the other hand, a balanced CNV is highly unlikely for glioblastoma in the adult setting. In addition, fractions of the diffuse astrocytoma IDH wt group with a balanced CNV pattern turn out to be low-grade gliomas [35]. However, this requires clear support from the methylation-based classification, because non-tumorous brain samples always exhibit a balanced CNV.

In conclusion, neuropathologists and clinicians in Heidelberg have come to appreciate methylation-based classification of CNS tumors as a useful tool to reduce diagnostic error, to direct patients to optimal postoperative treatment, and to stratify patients in clinical trials.

## Electronic supplementary material

Below is the link to the electronic supplementary material.

**Supplementary file 1** Summary CNV plots of all tumor-associated classes included in the CNS tumor *Classifier* in alphabetic order. (PDF 590 kb)

## References

[1] Bady P, Delorenzi M, Hegi ME (2016). Sensitivity analysis of the MGMT-STP27 model and impact of genetic and epigenetic context to predict the MGMT methylation status in gliomas and other tumors. J Mol Diagn.

[2] Bady P, Sciuscio D, Diserens AC (2012). MGMT methylation analysis of glioblastoma on the Infinium methylation BeadChip identifies two distinct CpG regions associated with gene silencing and outcome, yielding a prediction model for comparisons across datasets, tumor grades, and CIMP-status. Acta Neuropathol.

[3] Brastianos PK, Taylor-Weiner A, Manley PE (2014). Exome sequencing identifies BRAF mutations in papillary craniopharyngiomas. Nat Genet.

[4] Brat DJ, Verhaak RG, Cancer Genome Atlas Research N (2015). Comprehensive, integrative genomic analysis of diffuse lower-grade gliomas. N Engl J Med.

[5] Capper D, Engel NW, Stichel D et al (2018) DNA methylation-based reclassification of olfactory neuroblastoma. Acta Neuropathol. 10.1007/s00401-018-1854-7 10.1007/s00401-018-1854-7 29730775

[6] Capper D, Jones DTW, Sill M (2018). DNA methylation-based classification of central nervous system tumours. Nature.

[7] de Jong MC, Kors WA, de Graaf P, Castelijns JA, Kivela T, Moll AC (2014). Trilateral retinoblastoma: a systematic review and meta-analysis. Lancet Oncol.

[8] Deng MY, Sill M, Chiang J et al (2018) Molecularly defined diffuse leptomeningeal glioneuronal tumor (DLGNT) comprises two subgroups with distinct clinical and genetic features. Acta Neuropathol. 10.1007/s00401-018-1865-4 10.1007/s00401-018-1865-4 29766299

[9] El-Ayadi M, Egervari K, Merkler D (2018). Concurrent IDH1 and SMARCB1 Mutations in pediatric medulloblastoma; a case report. Front Neurol.

[10] Fernandez AF, Assenov Y, Martin-Subero JI (2012). A DNA methylation fingerprint of 1628 human samples. Genome Res.

[11] Goode B, Mondal G, Hyun M (2018). A recurrent kinase domain mutation in PRKCA defines chordoid glioma of the third ventricle. Nat Commun.

[12] Griewank G, Koelsche C, van de Nes J et al (2018) Integrated genomic classification of melanocytic tumors of the central nervous system using mutation analysis, copy number alterations and DNA methylation profiling. Clin Cancer Res. 10.1158/1078-0432.CCR-18-0763 10.1158/1078-0432.CCR-18-0763 29891723

[13] Heim S, Sill M, Jones DT (2016). Papillary tumor of the pineal region: a distinct molecular entity. Brain Pathol.

[14] Hovestadt V, Jones DT, Picelli S (2014). Decoding the regulatory landscape of medulloblastoma using DNA methylation sequencing. Nature.

[15] Hovestadt V, Zapatka M (2015) Conumee: enhanced copy-number variation analysis using Illumina methylation arrays. v.1.4.2 R package v.0.99.4. http://bioconductor.org/packages/conumee/

[16] Huse JT, Diamond EL, Wang L, Rosenblum MK (2015). Mixed glioma with molecular features of composite oligodendroglioma and astrocytoma: a true “oligoastrocytoma”?. Acta Neuropathol.

[17] Jakobiec FA, Kool M, Stagner AM, Pfister SM, Eagle RC, Proia AD, Korshunov A (2015). Intraocular medulloepitheliomas and embryonal tumors with multilayered rosettes of the brain: comparative roles of LIN28A and C19MC. Am J Ophthalmol.

[18] Johann PD, Erkek S, Zapatka M (2016). Atypical teratoid/rhabdoid tumors are comprised of three epigenetic subgroups with distinct enhancer landscapes. Cancer Cell.

[19] Koelsche C, Hovestadt V, Jones DT (2015). Melanotic tumors of the nervous system are characterized by distinct mutational, chromosomal and epigenomic profiles. Brain Pathol.

[20] Koelsche C, Sahm F, Paulus W (2014). BRAF V600E expression and distribution in desmoplastic infantile astrocytoma/ganglioglioma. Neuropathol Appl Neurobiol.

[21] Koelsche C, Wöhrer A, Jeibmann A, Schittenhelm J, Schindler G, Preusser M, Lasitschka F, von Deimling A, Capper D (2013). Mutant BRAF V600E protein in ganglioglioma is predominantly expressed by neuronal tumor cells. Acta Neuropathol.

[22] Kool M, Jones DT, Jager N (2014). Genome sequencing of SHH medulloblastoma predicts genotype-related response to smoothened inhibition. Cancer Cell.

[23] Korshunov A, Capper D, Reuss D (2016). Histologically distinct neuroepithelial tumors with histone 3 G34 mutation are molecularly similar and comprise a single nosologic entity. Acta Neuropathol.

[24] Korshunov A, Chavez L, Sharma T et al (2017) Epithelioid glioblastomas stratify into established diagnostic subsets upon integrated molecular analysis. Brain Pathol. 10.1111/bpa.12566 10.1111/bpa.12566 PMC7469088 28990704

[25] Korshunov A, Jakobiec FA, Eberhart CG (2015). Comparative integrated molecular analysis of intraocular medulloepitheliomas and central nervous system embryonal tumors with multilayered rosettes confirms that they are distinct nosologic entities. Neuropathology.

[26] Korshunov A, Ryzhova M, Hovestadt V (2015). Integrated analysis of pediatric glioblastoma reveals a subset of biologically favorable tumors with associated molecular prognostic markers. Acta Neuropathol.

[27] Letouze E, Martinelli C, Loriot C (2013). SDH mutations establish a hypermethylator phenotype in paraganglioma. Cancer Cell.

[28] Louis D, Ohgaki H, Wiestler O, Cavenee WK, Bosman F, Jaffe E, Lakhani S, Ohgaki H (2016). World Health Organization classification of tumours of the central nervous system. World Health Organization classification of tumours revised.

[29] Louis DN, Aldape K, Brat DJ (2017). Announcing cIMPACT-NOW: the Consortium to inform molecular and practical approaches to CNS tumor taxonomy. Acta Neuropathol.

[30] Louis DN, Wesseling P, Paulus W (2018). cIMPACT-NOW update 1: not otherwise specified (NOS) and not elsewhere classified (NEC). Acta Neuropathol.

[31] Mackay A, Burford A, Molinari V (2018). Molecular, pathological, radiological, and immune profiling of non-brainstem pediatric high-grade glioma from the HERBY Phase II randomized trial. Cancer Cell.

[32] Pajtler KW, Witt H, Sill M (2015). Molecular classification of ependymal tumors across all cns compartments, histopathological grades, and age groups. Cancer Cell.

[33] Parker M, Mohankumar KM, Punchihewa C (2014). C11orf95-RELA fusions drive oncogenic NF-kappaB signalling in ependymoma. Nature.

[34] Reinhardt A, Stichel D, Schrimpf D et al (2018) Anaplastic astrocytoma with piloid features, a novel molecular class of IDH wild-type glioma with recurrent MAPK pathway, CDKN2A/B and ATRX alterations. Acta Neuropathol. 10.1007/s00401-018-1837-8 10.1007/s00401-018-1837-8 29564591

[35] Reuss DE, Kratz A, Sahm F (2015). Adult IDH wild type astrocytomas biologically and clinically resolve into other tumor entities. Acta Neuropathol.

[36] Reuss DE, Sahm F, Schrimpf D (2015). ATRX and IDH1-R132H immunohistochemistry with subsequent copy number analysis and IDH sequencing as a basis for an “integrated” diagnostic approach for adult astrocytoma, oligodendroglioma and glioblastoma. Acta Neuropathol.

[37] Robinson JT, Thorvaldsdottir H, Winckler W, Guttman M, Lander ES, Getz G, Mesirov JP (2011). Integrative genomics viewer. Nat Biotechnol.

[38] Rodriguez FJ, Perry A, Rosenblum MK (2012). Disseminated oligodendroglial-like leptomeningeal tumor of childhood: a distinctive clinicopathologic entity. Acta Neuropathol.

[39] Rohrich M, Koelsche C, Schrimpf D (2016). Methylation-based classification of benign and malignant peripheral nerve sheath tumors. Acta Neuropathol.

[40] Sahm F, Jakobiec FA, Meyer J (2016). Somatic mutations of DICER1 and KMT2D are frequent in intraocular medulloepitheliomas. Genes Chromosom Cancer.

[41] Sahm F, Schrimpf D, Jones DT (2016). Next-generation sequencing in routine brain tumor diagnostics enables an integrated diagnosis and identifies actionable targets. Acta Neuropathol.

[42] Sahm F, Schrimpf D, Stichel D (2017). DNA methylation-based classification and grading system for meningioma: a multicentre, retrospective analysis. Lancet Oncol.

[43] Sekine S, Shibata T, Kokubu A, Morishita Y, Noguchi M, Nakanishi Y, Sakamoto M, Hirohashi S (2002). Craniopharyngiomas of adamantinomatous type harbor beta-catenin gene mutations. Am J Pathol.

[44] Shirahata M, Ono T, Stichel D (2018). Novel, improved grading system(s) for IDH-mutant astrocytic gliomas. Acta Neuropathol.

[45] Sturm D, Orr BA, Toprak UH (2016). New Brain Tumor Entities Emerge from Molecular Classification of CNS-PNETs. Cell.

[46] Sturm D, Witt H, Hovestadt V (2012). Hotspot mutations in H3F3A and IDH1 define distinct epigenetic and biological subgroups of glioblastoma. Cancer Cell.

[47] Suzuki H, Aoki K, Chiba K (2015). Mutational landscape and clonal architecture in grade II and III gliomas. Nat Genet.

[48] Taylor M, Northcott P, Korshunov A (2012). Molecular subgroups of medulloblastoma: the current consensus. Acta Neuropathol.

[49] Thom M, Blumcke I, Aronica E (2012). Long-term epilepsy-associated tumors. Brain Pathol.

[50] Thomas C, Sill M, Ruland V (2016). Methylation profiling of choroid plexus tumors reveals 3 clinically distinct subgroups. Neuro Oncol.

[51] Weber RG, Hoischen A, Ehrler M (2007). Frequent loss of chromosome 9, homozygous CDKN2A/p14(ARF)/CDKN2B deletion and low TSC1 mRNA expression in pleomorphic xanthoastrocytomas. Oncogene.

[52] Wiestler B, Capper D, Sill M (2014). Integrated DNA methylation and copy-number profiling identify three clinically and biologically relevant groups of anaplastic glioma. Acta Neuropathol.

[53] Wilcox P, Li CC, Lee M, Shivalingam B, Brennan J, Suter CM, Kaufman K, Lum T, Buckland ME (2015). Oligoastrocytomas: throwing the baby out with the bathwater?. Acta Neuropathol.

[54] Witt H, Mack S, Ryzhova M (2011). Delineation of two clinically and molecularly distinct subgroups of posterior fossa ependymoma. Cancer Cell.

[55] Wood MD, Tihan T, Perry A (2018). Multimodal molecular analysis of astroblastoma enables reclassification of most cases into more specific molecular entities. Brain Pathol.

[56] Worst BC, van Tilburg CM, Balasubramanian GP (2016). Next-generation personalised medicine for high-risk paediatric cancer patients—the INFORM pilot study. Eur J Cancer.

