# Supplementary material for: Practical implementation of DNA methylation and copy-number-based CNS tumor diagnostics: the Heidelberg experience
Source: Acta Neuropathol. 2018 Jul 2;136(2):181–210. doi: 10.1007/s00401-018-1879-y (PMC6060790; doi:10.1007/s00401-018-1879-y)
Supplement: Supplementary file 1 — Summary CNV plots of all tumor-associated classes included in the CNS tumor Classifier in alphabetic order. (PDF 590 kb) [file 401_2018_1879_MOESM1_ESM.pdf]

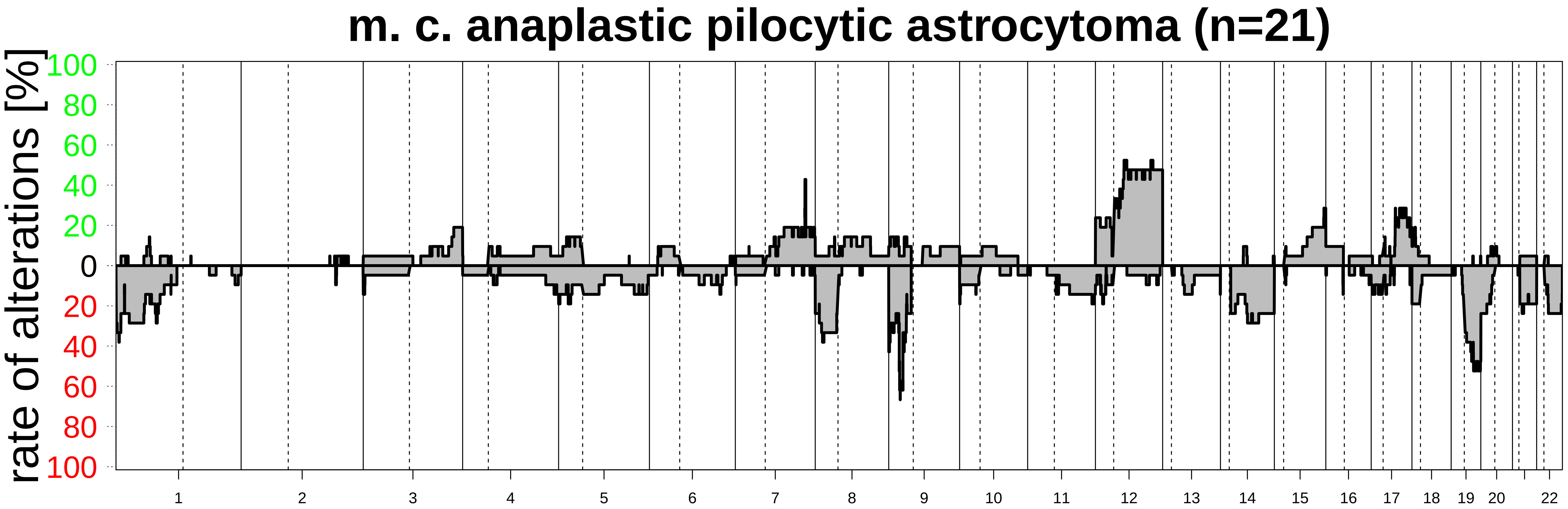
m. c. anaplastic pilocytic astrocytoma (n=21)
rate of alterations [%]
100
80
60
40
20
0
20
40
60
80
100
1
2
3
4
5
6
7
8
9
10
11
12
13
14
15
16
17
18
19
20
22

m. c. (anaplastic) pleomorphic xanthoastrocytoma (n=44)

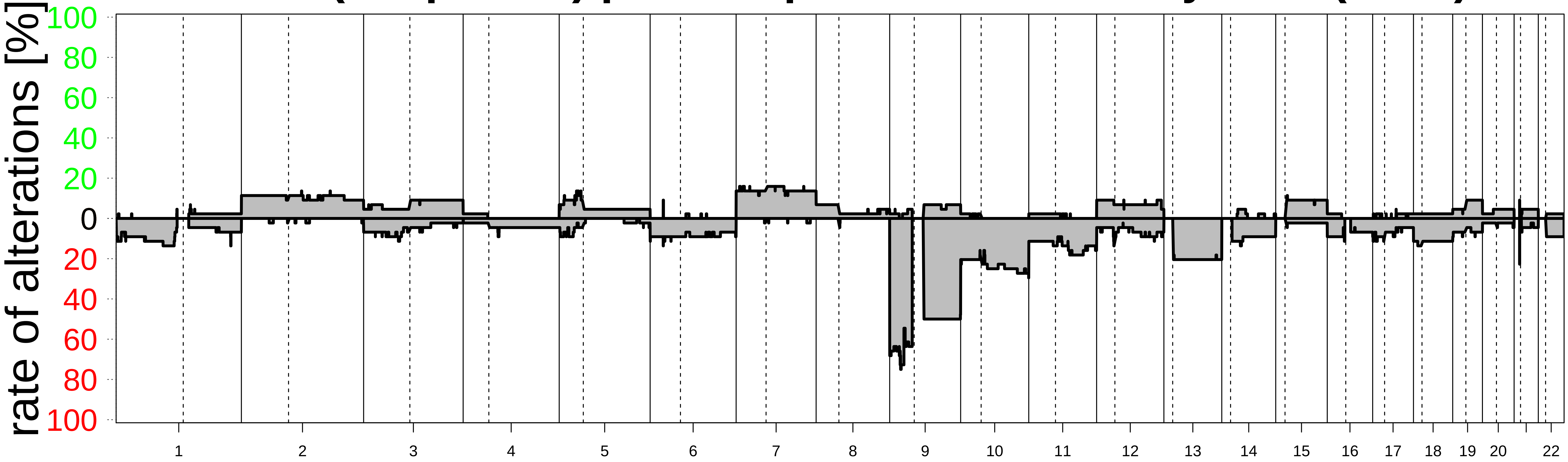

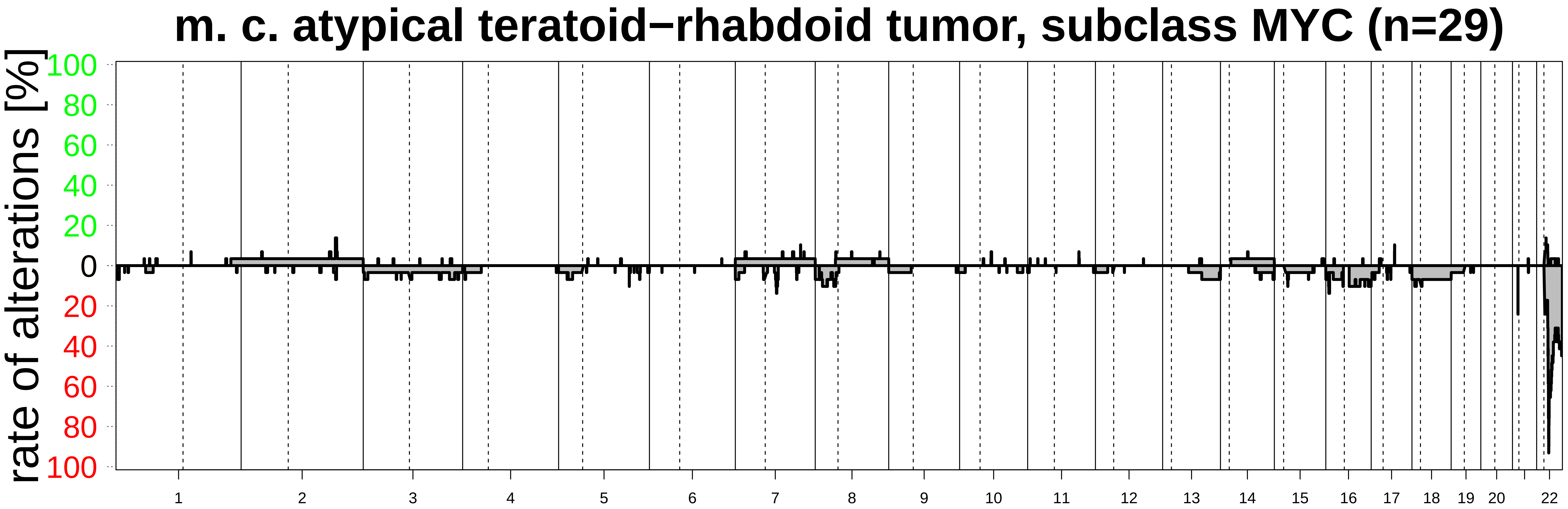
m. c. atypical teratoid−rhabdoid tumor, subclass MYC (n=29)
rate of alterations [%]
100
80
60
40
20
0
20
40
60
80
100
1
2
3
4
5
6
7
8
9
10
11
12
13
14
15
16
17
18
19
20
22

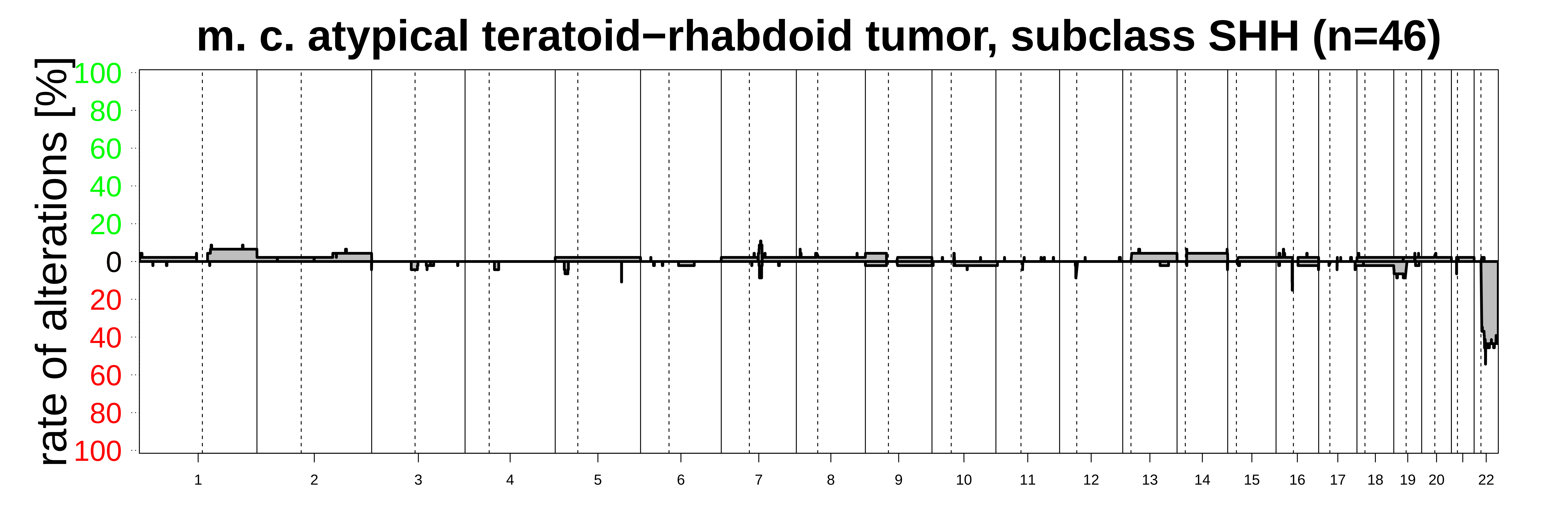
m. c. atypical teratoid−rhabdoid tumor, subclass SHH (n=46)
rate of alterations [%]
100
80
60
40
20
0
20
40
60
80
100
1
2
3
4
5
6
7
8
9
10
11
12
13
14
15
16
17
18
19
20
22

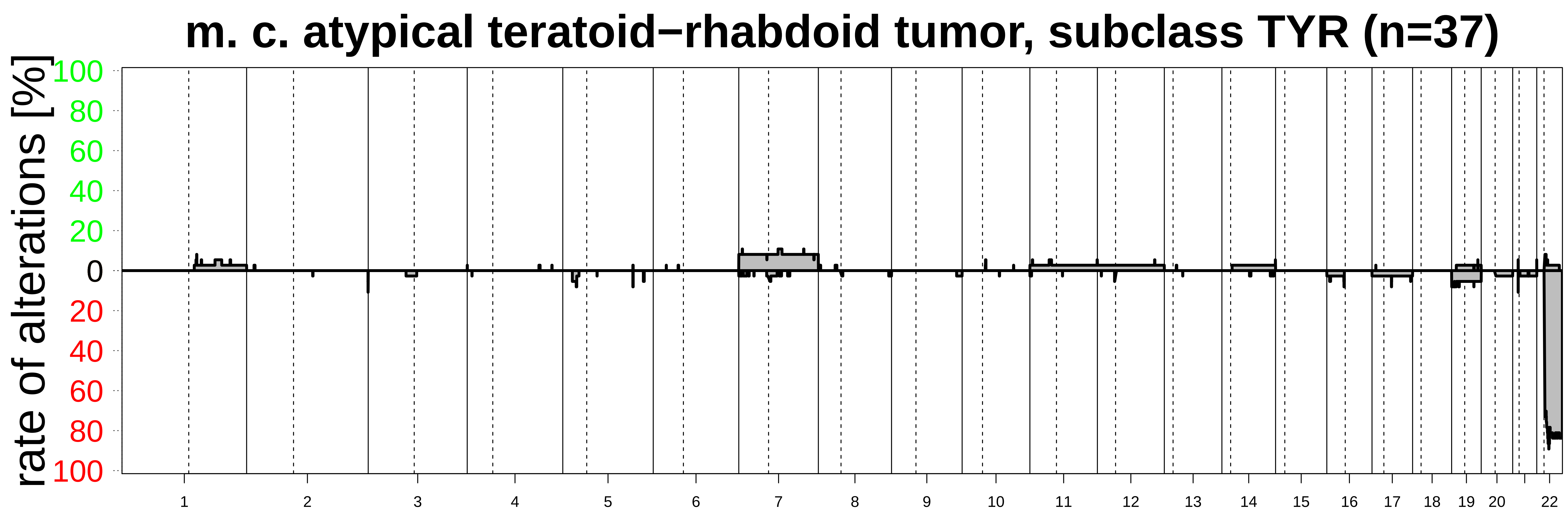
m. c. atypical teratoid−rhabdoid tumor, subclass TYR (n=37)
rate of alterations [%]
100
80
60
40
20
0
20
40
60
80
100
1
2
3
4
5
6
7
8
9
10
11
12
13
14
15
16
17
18
19
20
22

m. c. central neurocytoma (n=21)

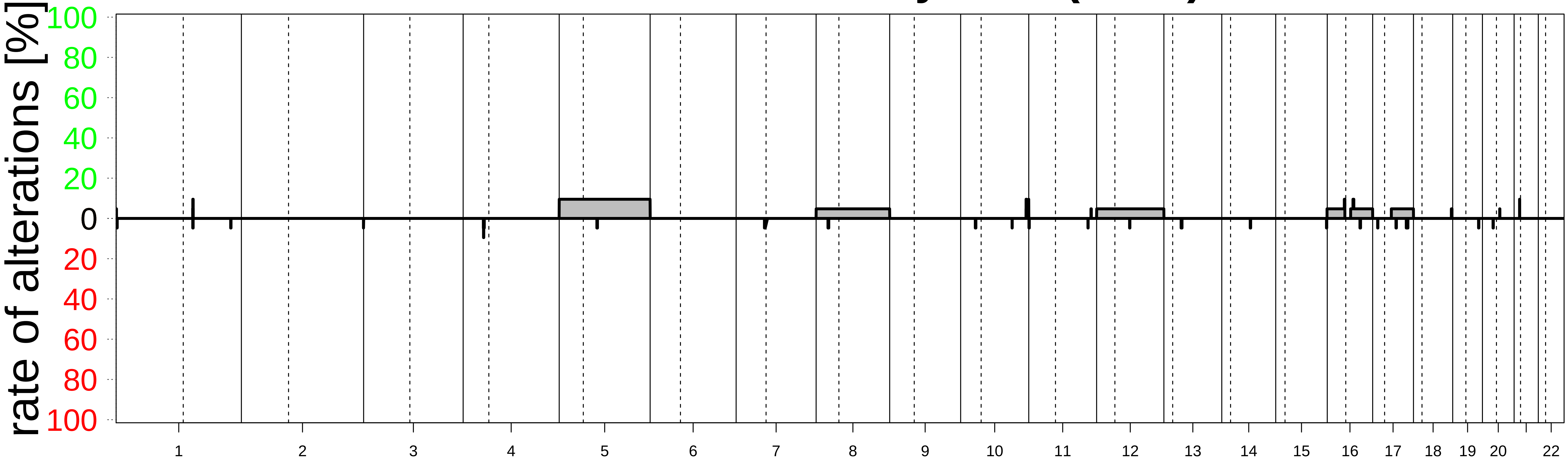

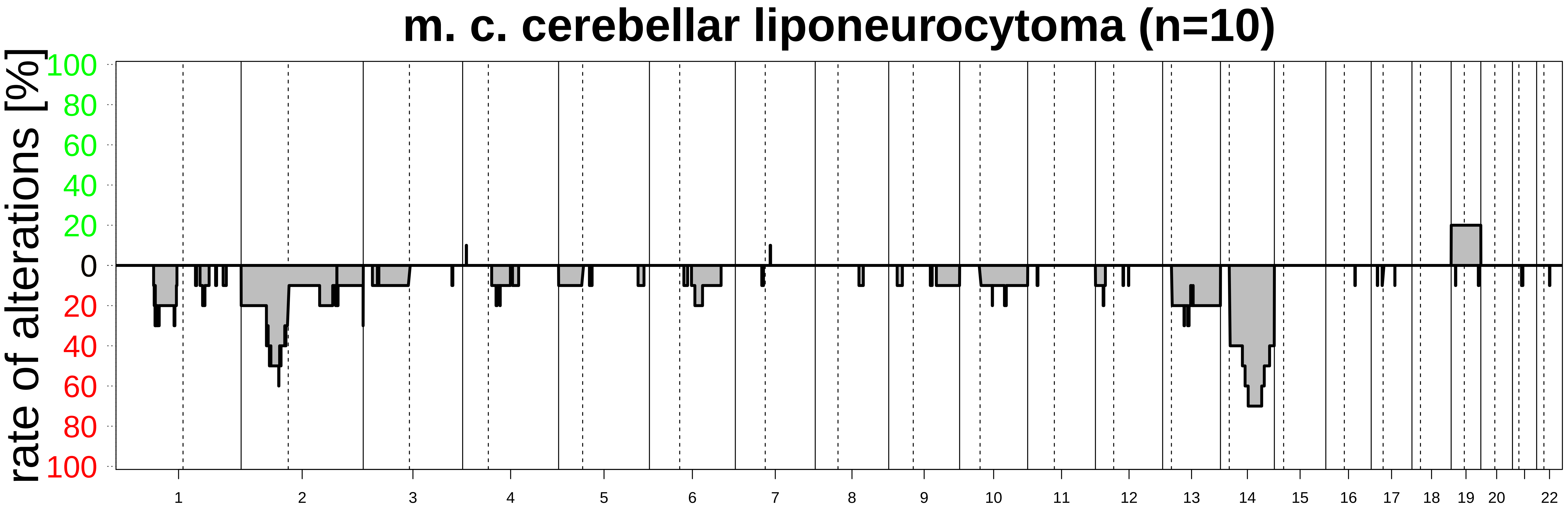
m. c. cerebellar liponeurocytoma (n=10)
rate of alterations [%]
100
80
60
40
20
0
20
40
60
80
100
1
2
3
4
5
6
7
8
9
10
11
12
13
14
15
16
17
18
19
20
22

m. c. chordoid glioma of the third ventricle (n=12)

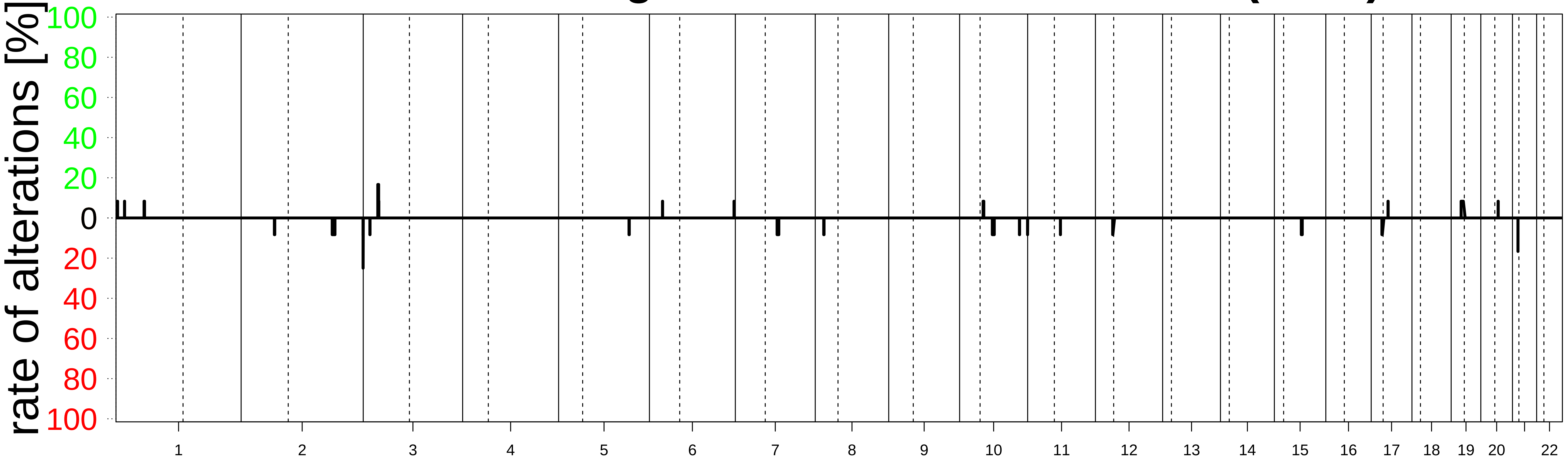

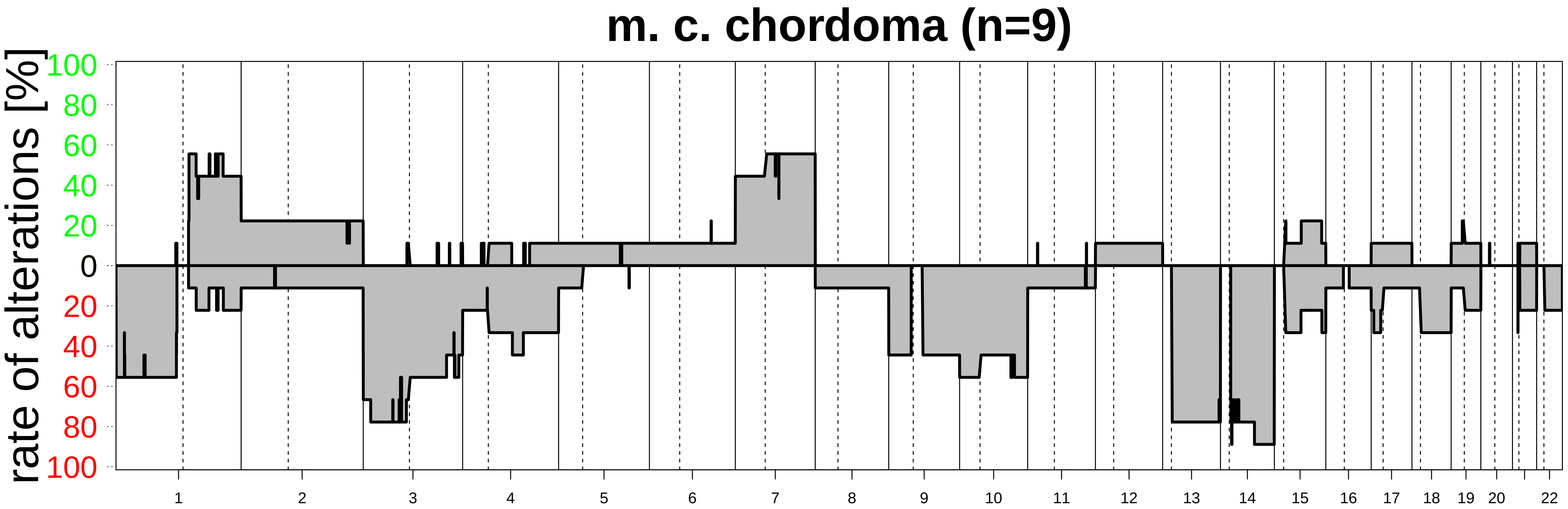

m. c. chordoma (n=9)
rate of alterations [%]
100
80
60
40
20
0
20
40
60
80
100
1
2
3
4
5
6
7
8
9
10
11
12
13
14
15
16
17
18
19
20
22

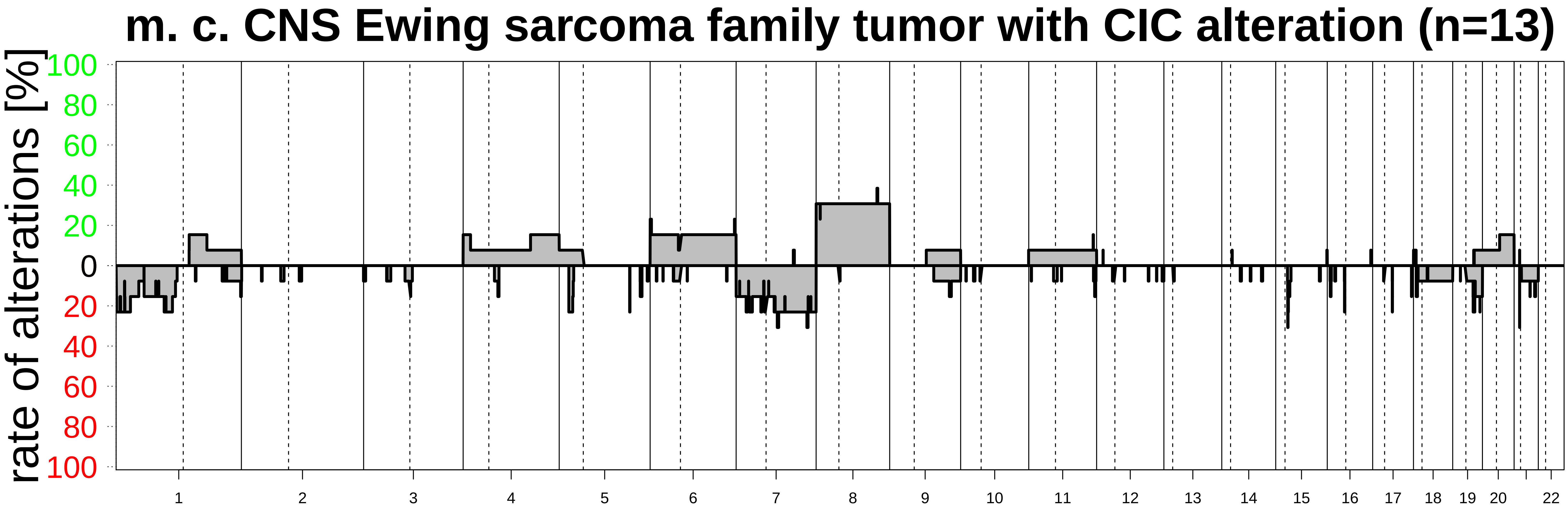
m. c. CNS Ewing sarcoma family tumor with CIC alteration (n=13)
rate of alterations [%]
100
80
60
40
20
0
20
40
60
80
100
1
2
3
4
5
6
7
8
9
10
11
12
13
14
15
16
17
18
19
20
22

m. c. CNS high grade neuroepithelial tumor with BCOR alteration (n=23)

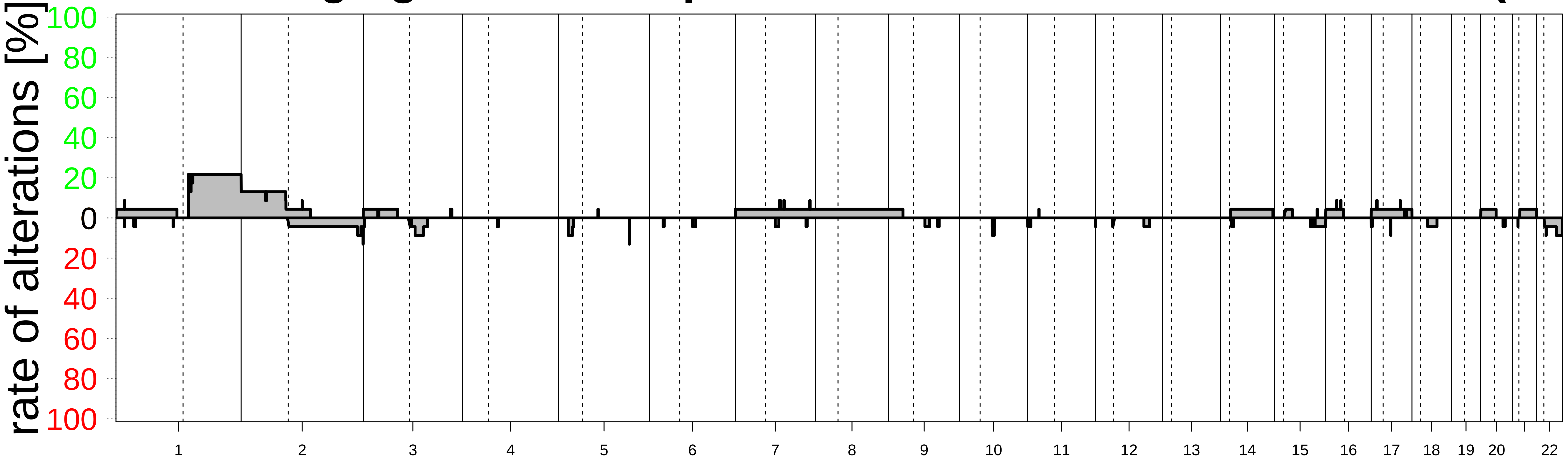

m. c. CNS high grade neuroepithelial tumor with MN1 alteration (n=21)

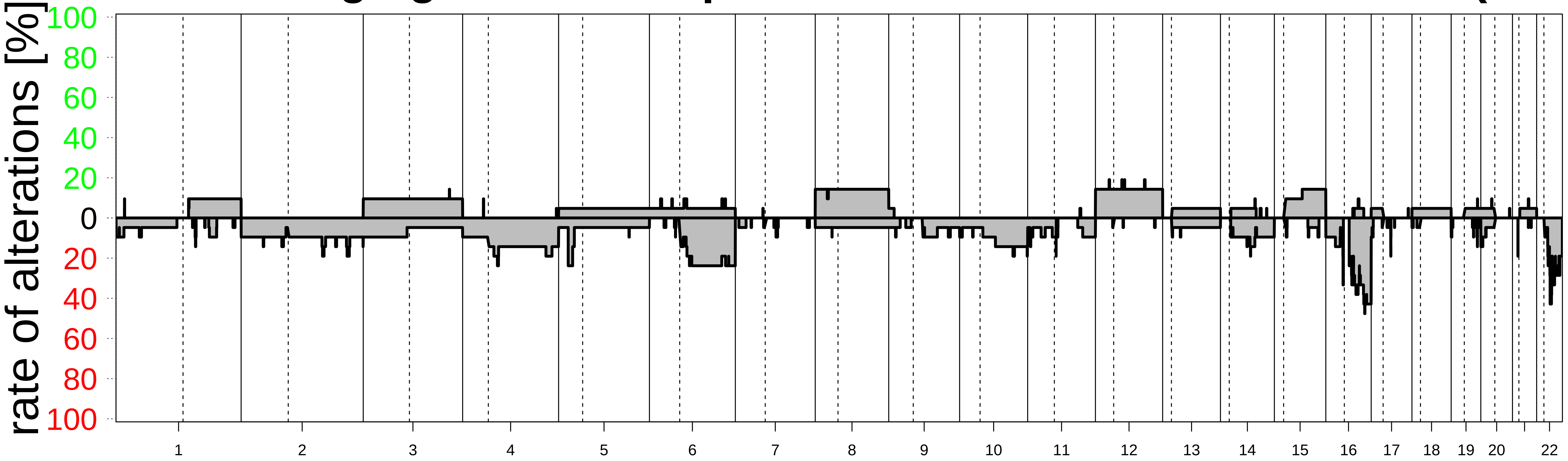

m. c. CNS neuroblastoma with FOXR2 activation (n=39)

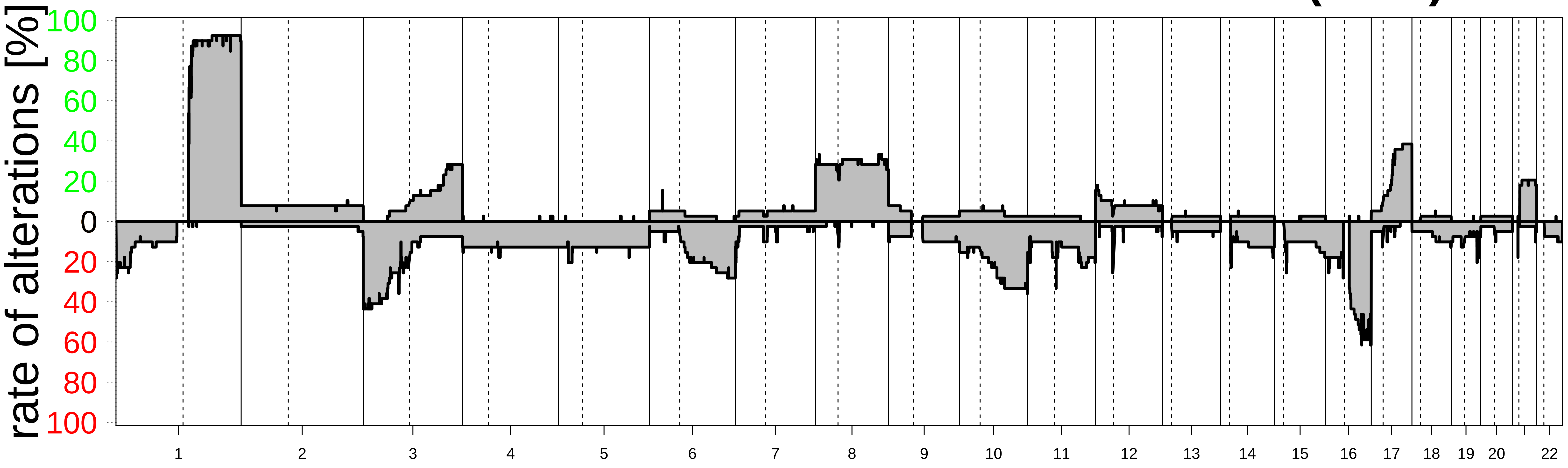

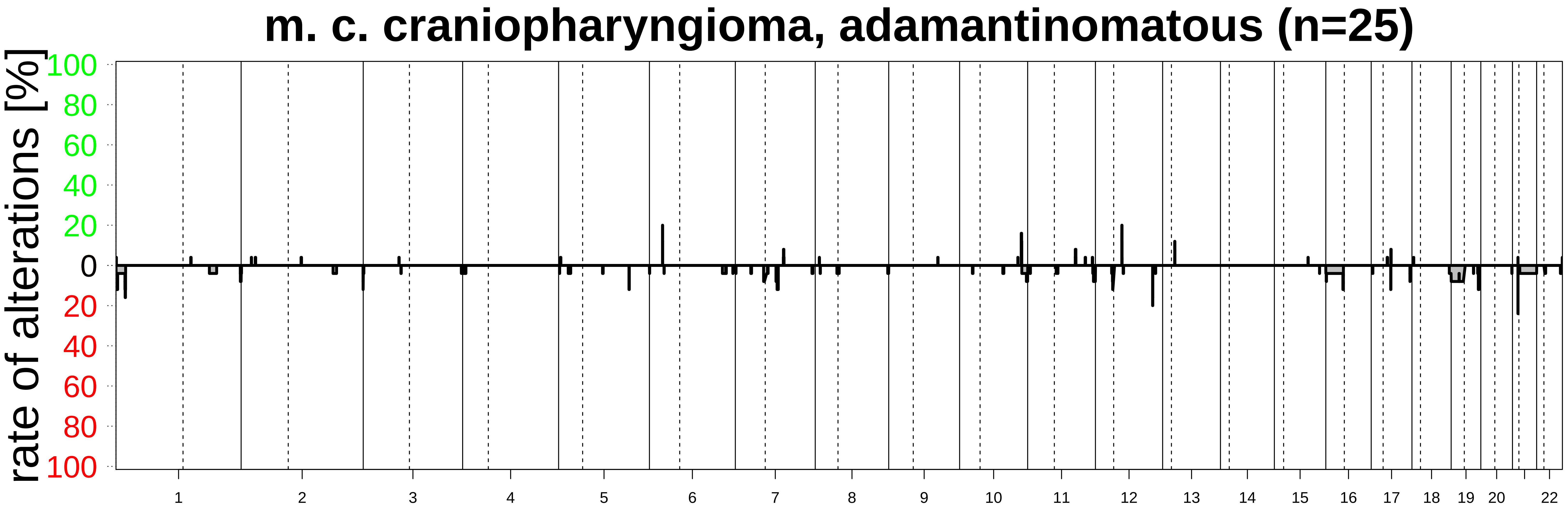
m. c. craniopharyngioma, adamantinomatous (n=25)
rate of alterations [%]
100
80
60
40
20
0
20
40
60
80
100
1
2
3
4
5
6
7
8
9
10
11
12
13
14
15
16
17
18
19
20
22

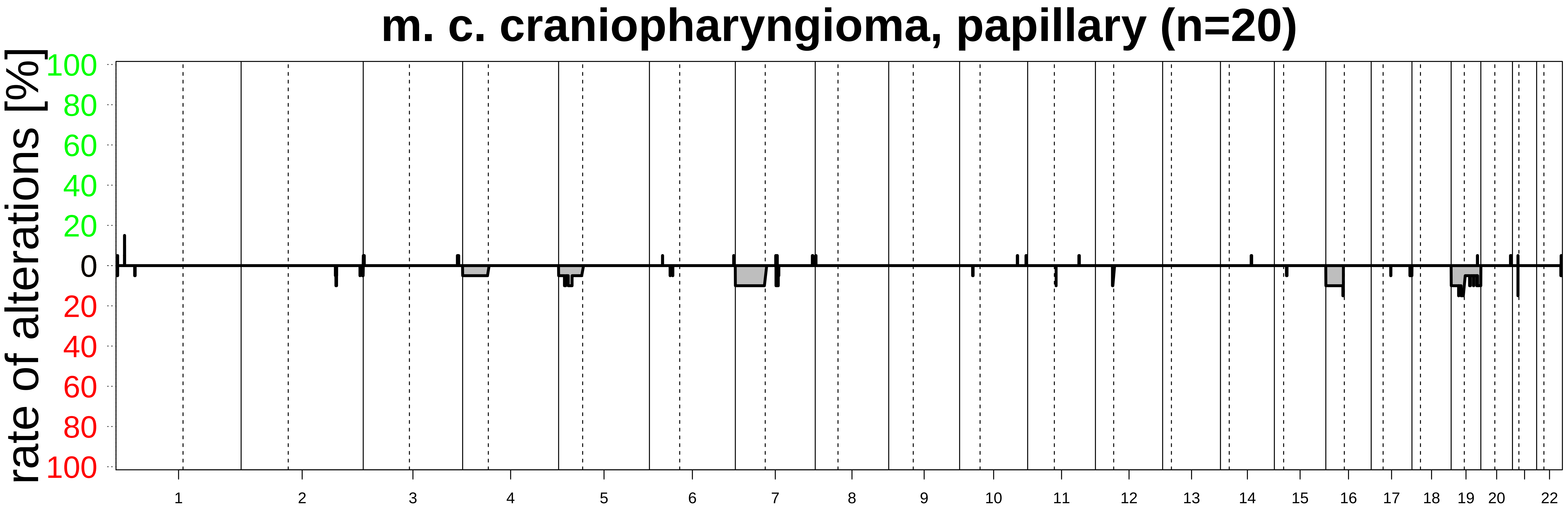

m. c. craniopharyngioma, papillary (n=20)
rate of alterations [%]
100
80
60
40
20
0
20
40
60
80
100
1
2
3
4
5
6
7
8
9
10
11
12
13
14
15
16
17
18
19
20
22

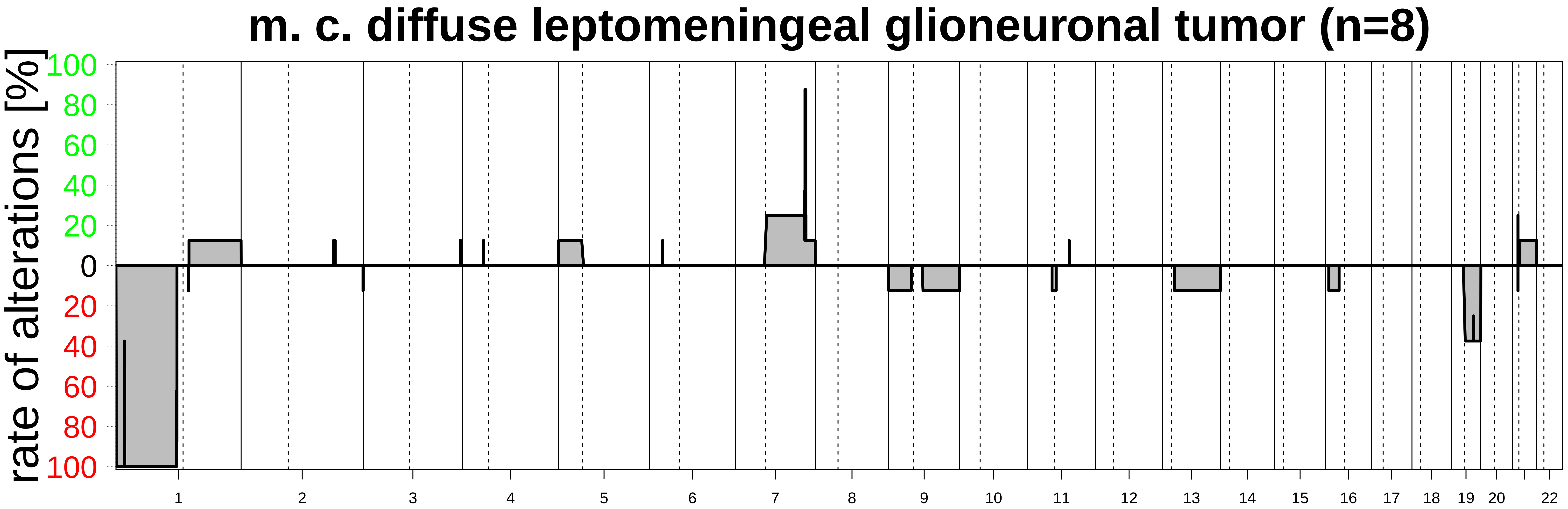

m. c. diffuse leptomeningeal glioneuronal tumor (n=8)
rate of alterations [%]
100
80
60
40
20
0
20
40
60
80
100
1
2
3
4
5
6
7
8
9
10
11
12
13
14
15
16
17
18
19
20
22

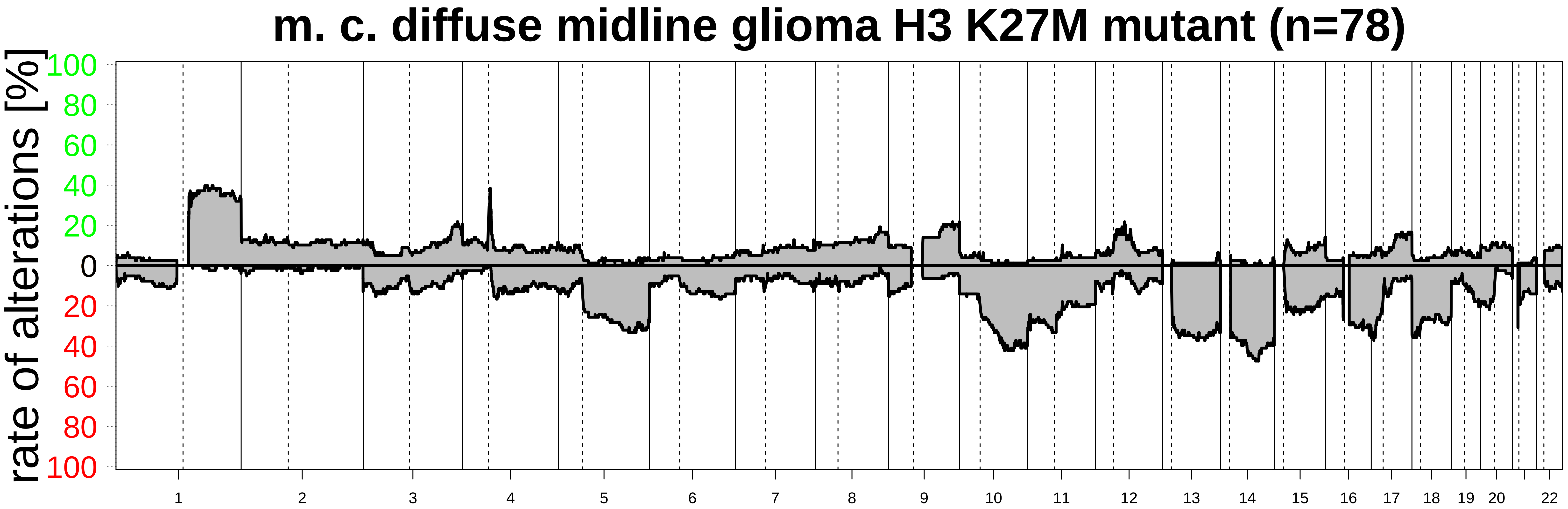
m. c. diffuse midline glioma H3 K27M mutant (n=78)
rate of alterations [%]
100
80
60
40
20
0
20
40
60
80
100
1
2
3
4
5
6
7
8
9
10
11
12
13
14
15
16
17
18
19
20
22

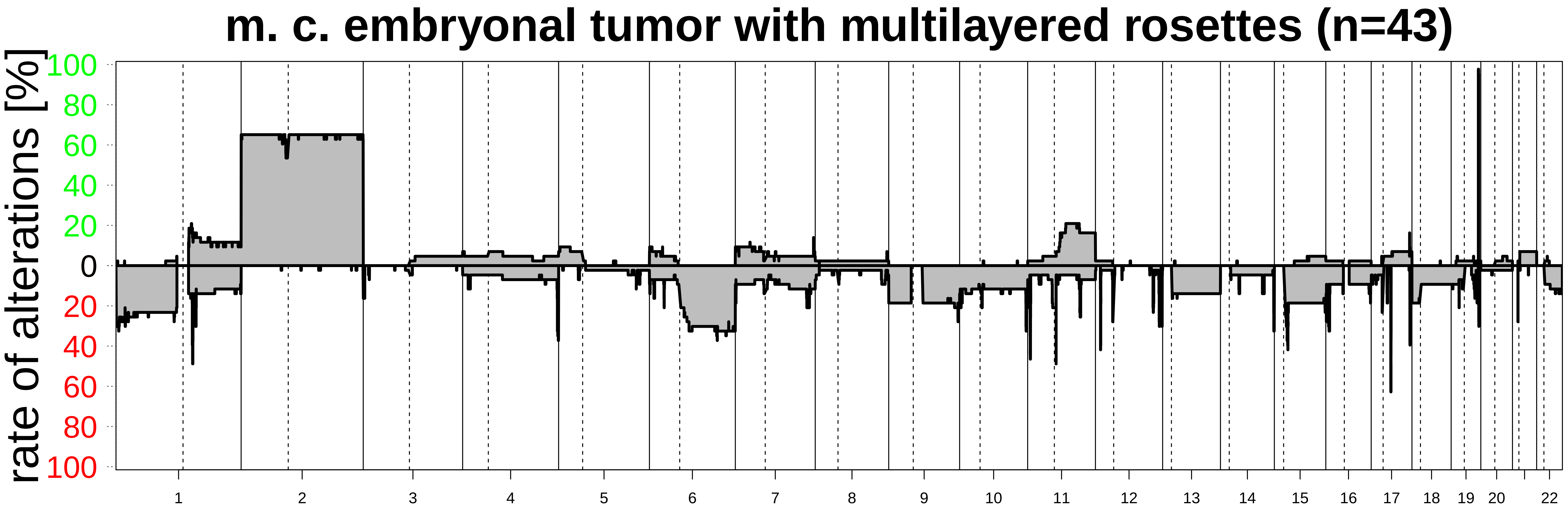
m. c. embryonal tumor with multilayered rosettes (n=43)
rate of alterations [%]
100
80
60
40
20
0
20
40
60
80
100
1
2
3
4
5
6
7
8
9
10
11
12
13
14
15
16
17
18
19
20
22

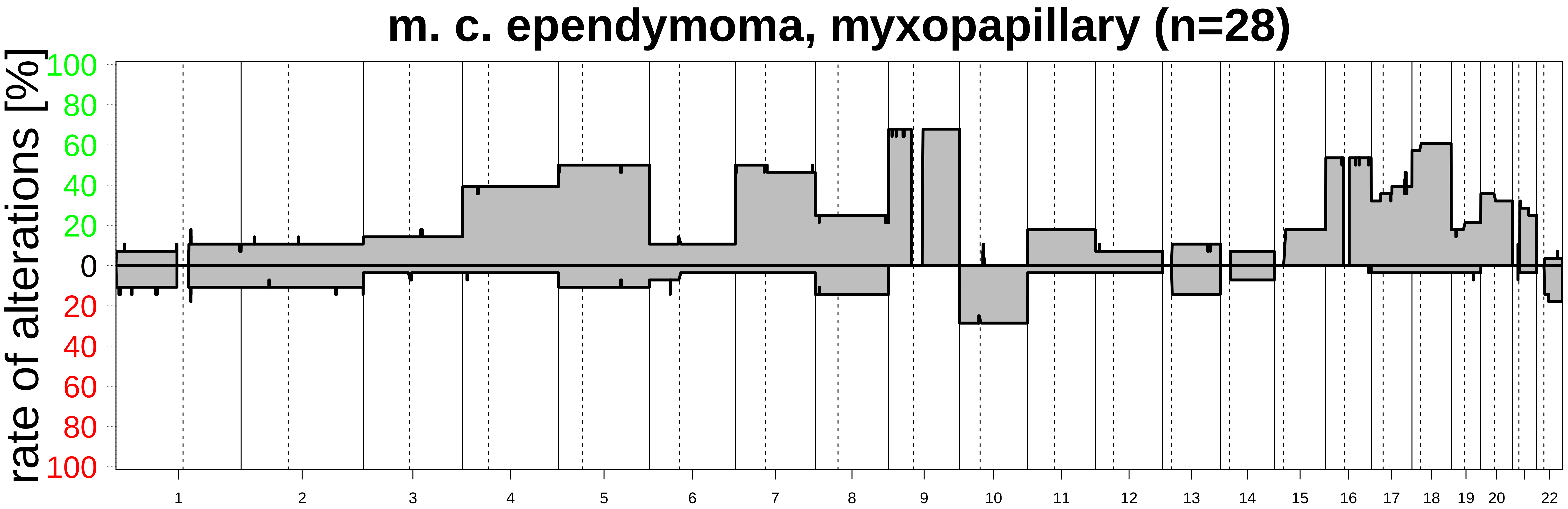
m. c. ependymoma, myxopapillary (n=28)
rate of alterations [%]
100
80
60
40
20
0
20
40
60
80
100
1
2
3
4
5
6
7
8
9
10
11
12
13
14
15
16
17
18
19
20
22

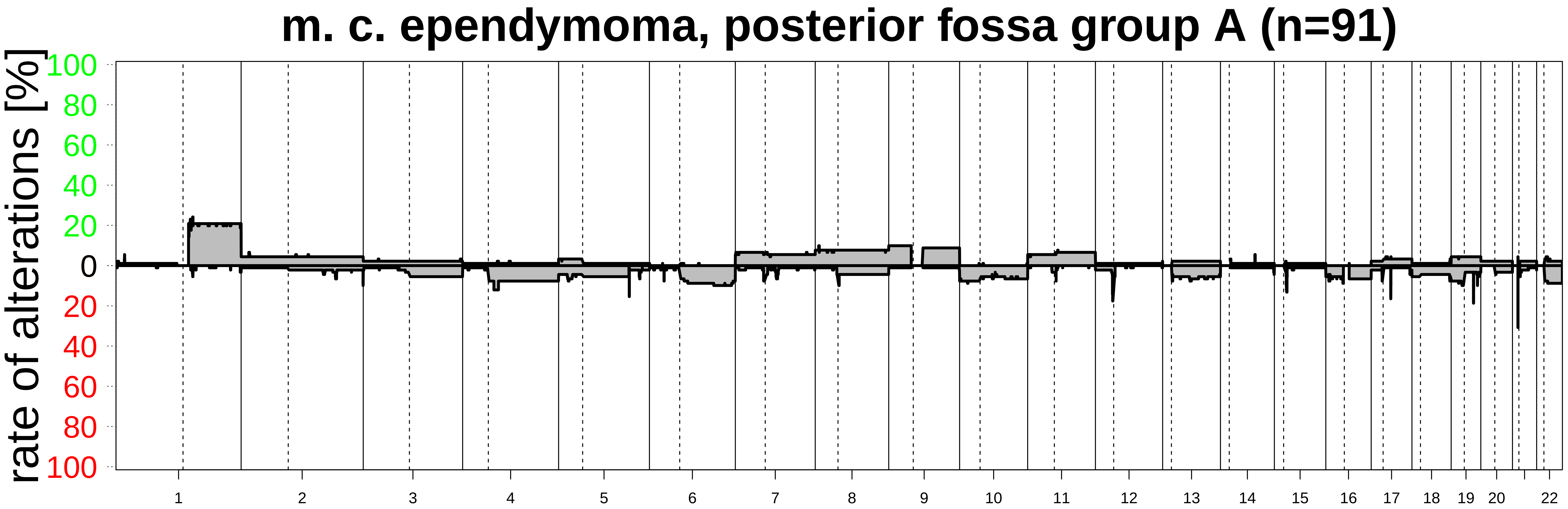
m. c. ependymoma, posterior fossa group A (n=91)
rate of alterations [%]
100
80
60
40
20
0
20
40
60
80
100
1
2
3
4
5
6
7
8
9
10
11
12
13
14
15
16
17
18
19
20
22

m. c. ependymoma, posterior fossa group B (n=51)

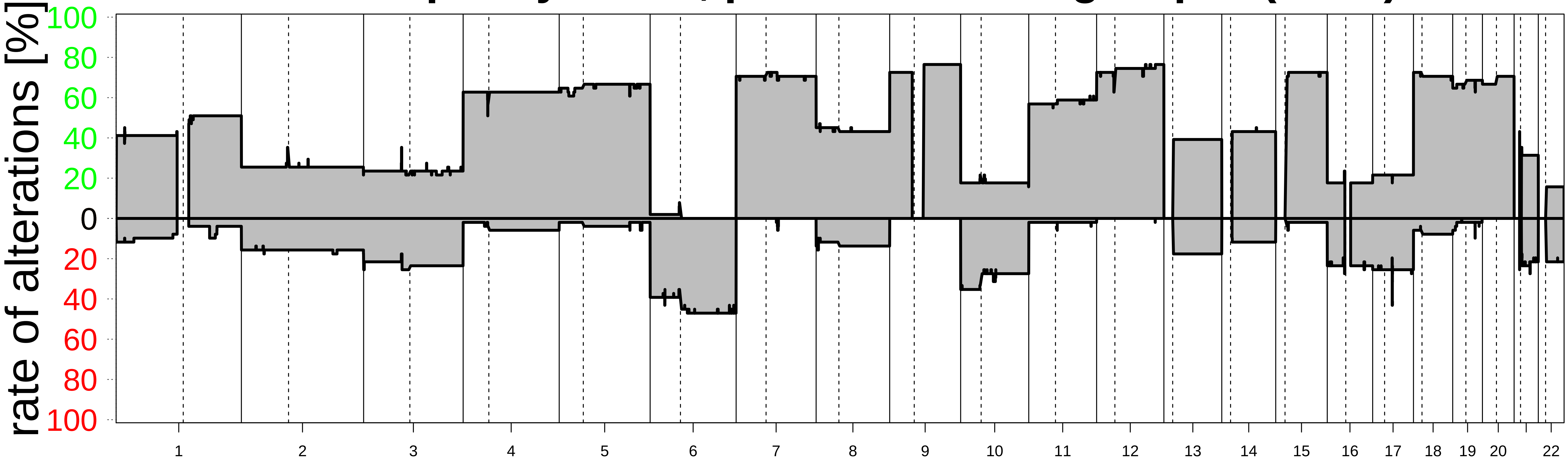

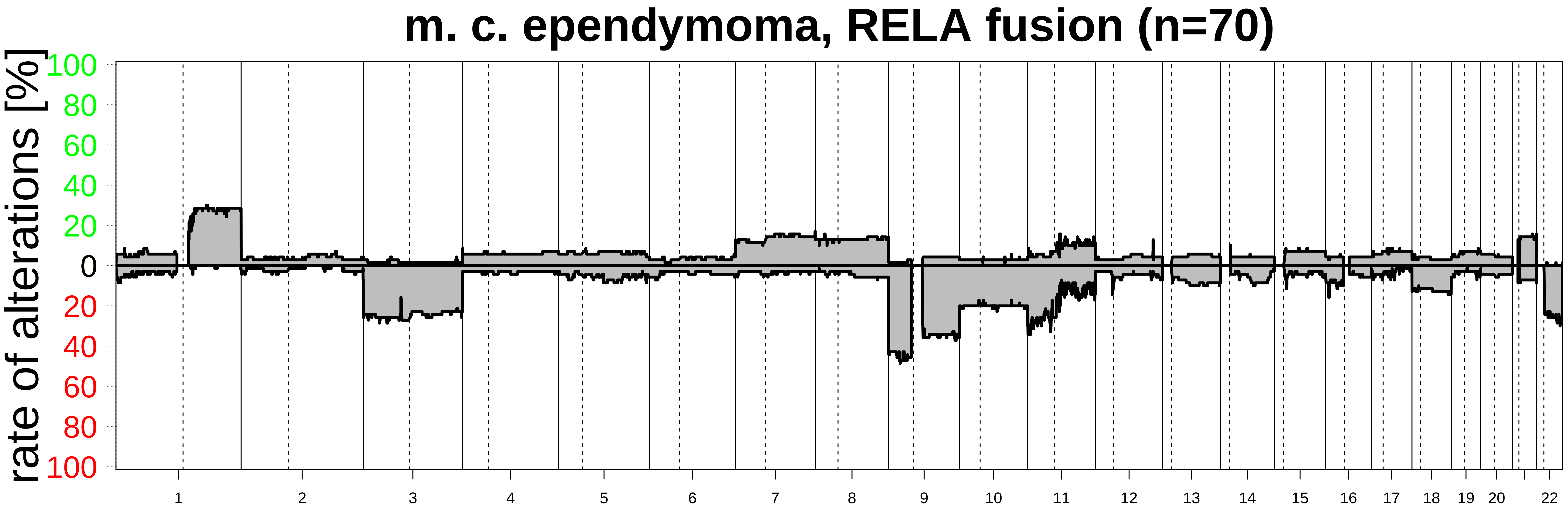

m. c. ependymoma, RELA fusion (n=70)
rate of alterations [%]
100
80
60
40
20
0
20
40
60
80
100
1
2
3
4
5
6
7
8
9
10
11
12
13
14
15
16
17
18
19
20
22

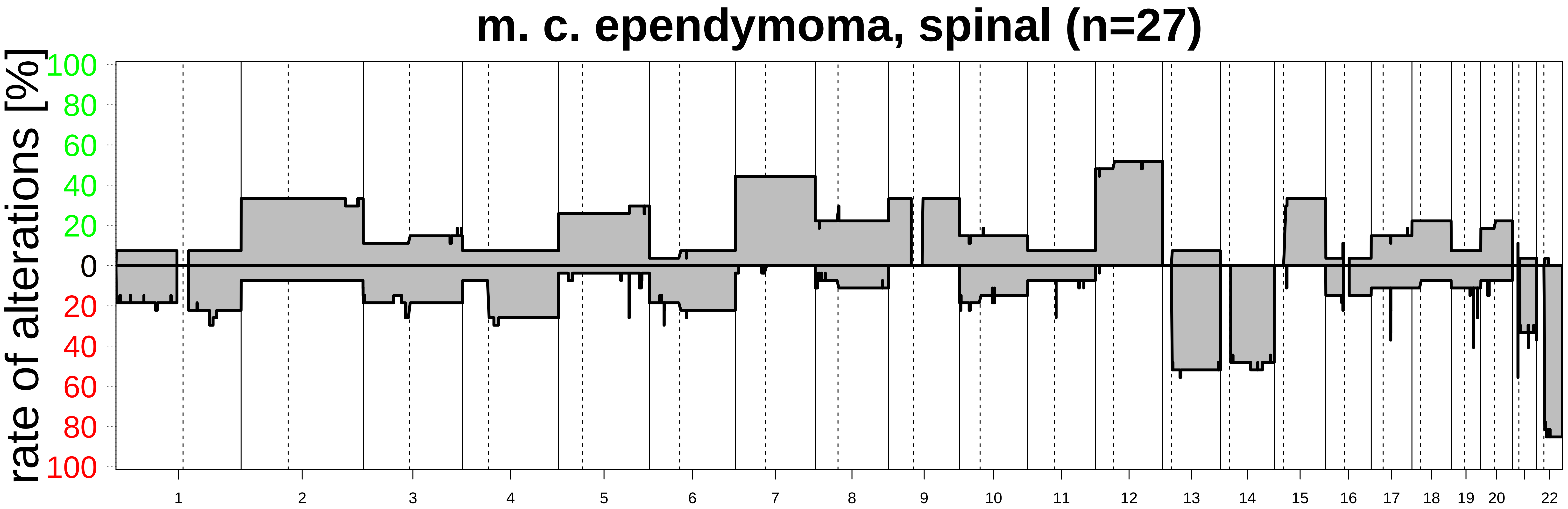
m. c. ependymoma, spinal (n=27)
rate of alterations [%]
100
80
60
40
20
0
20
40
60
80
100
1
2
3
4
5
6
7
8
9
10
11
12
13
14
15
16
17
18
19
20
22

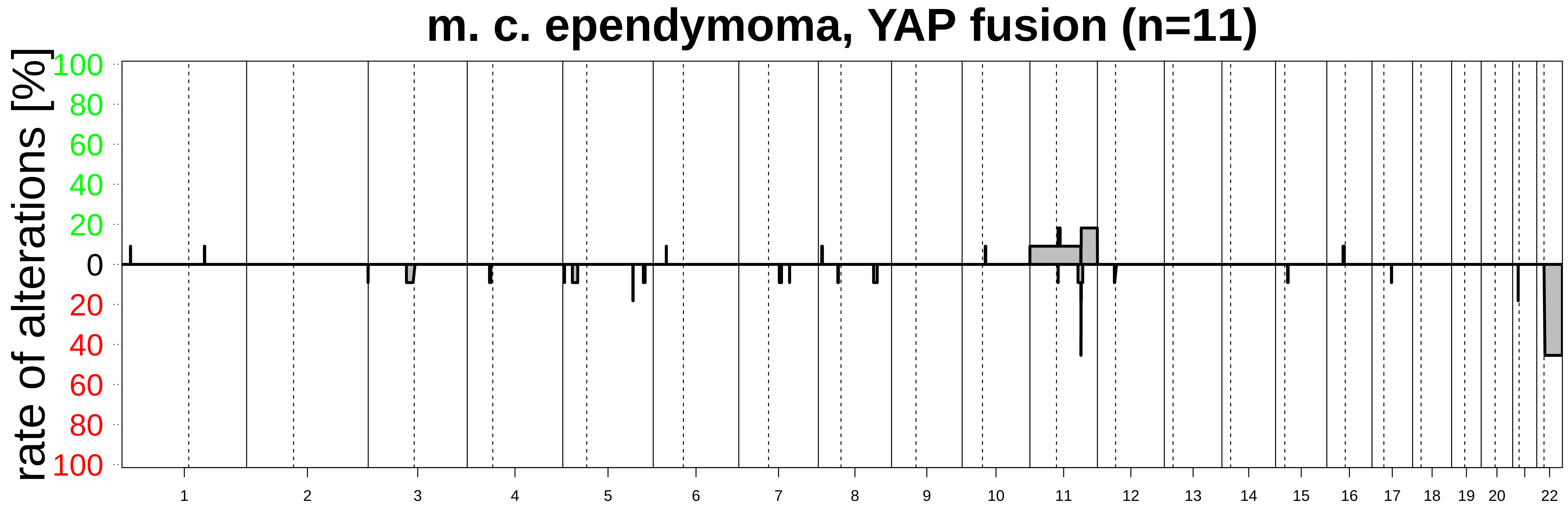
m. c. ependymoma, YAP fusion (n=11)
rate of alterations [%]
100
80
60
40
20
0
20
40
60
80
100
1
2
3
4
5
6
7
8
9
10
11
12
13
14
15
16
17
18
19
20
22

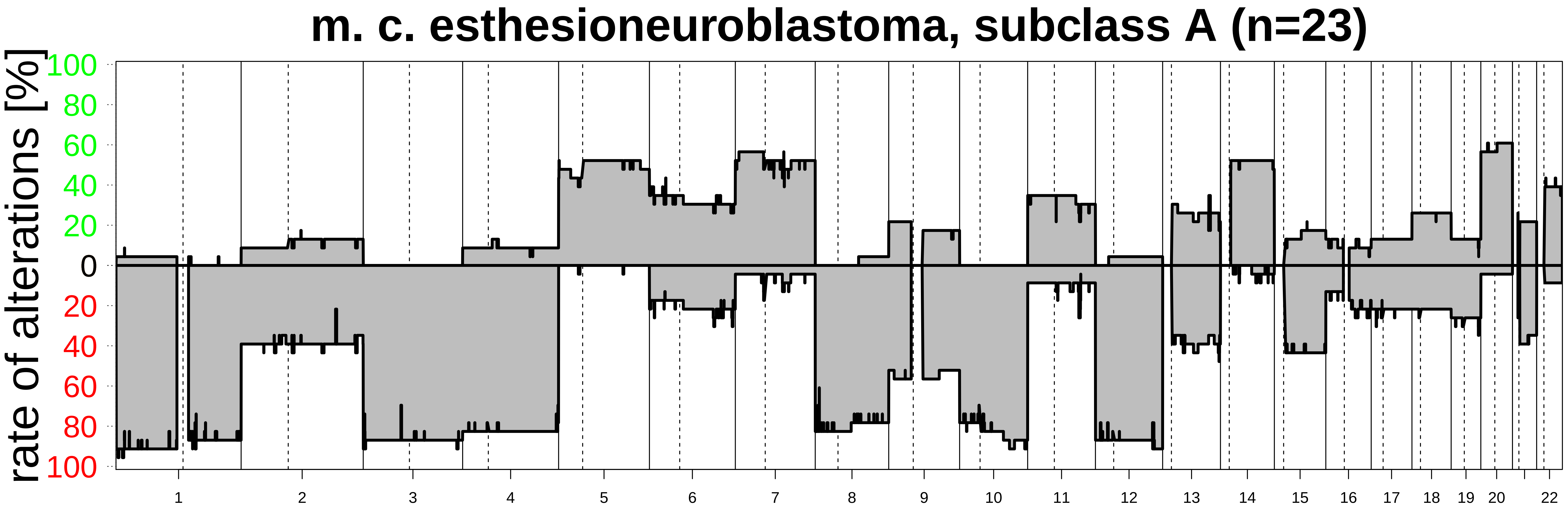

m. c. esthesioneuroblastoma, subclass A (n=23)
rate of alterations [%]
100
80
60
40
20
0
20
40
60
80
100
1
2
3
4
5
6
7
8
9
10
11
12
13
14
15
16
17
18
19
20
22

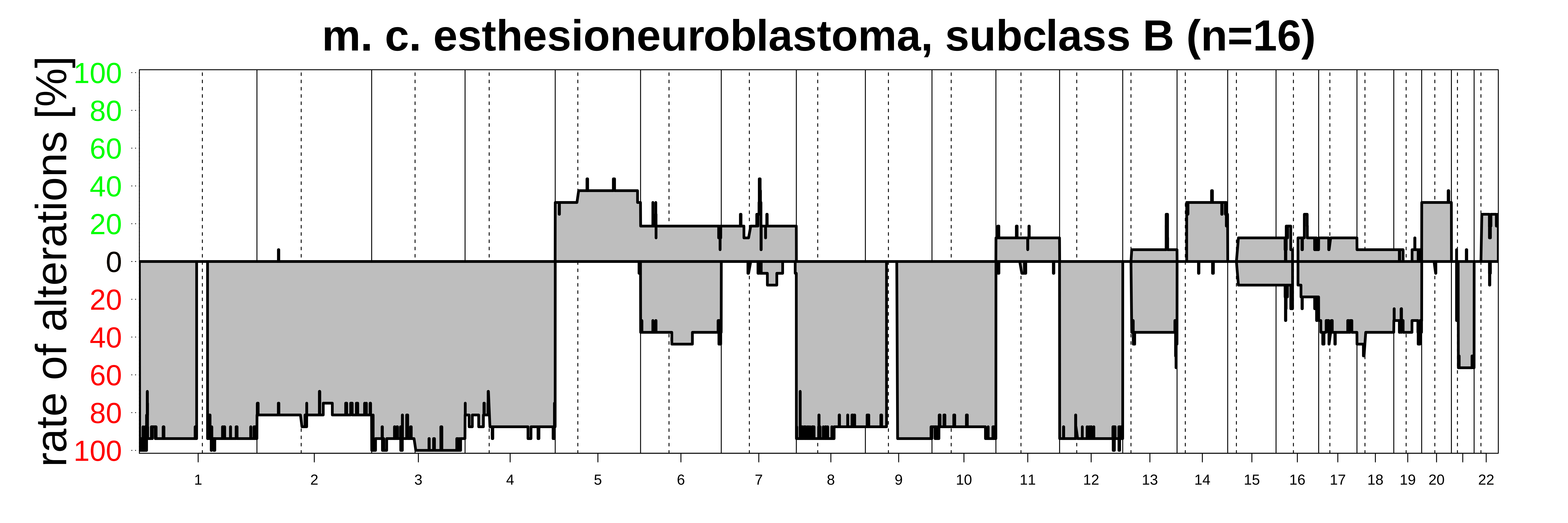
m. c. esthesioneuroblastoma, subclass B (n=16)
rate of alterations [%]
100
80
60
40
20
0
20
40
60
80
100
1
2
3
4
5
6
7
8
9
10
11
12
13
14
15
16
17
18
19
20
22

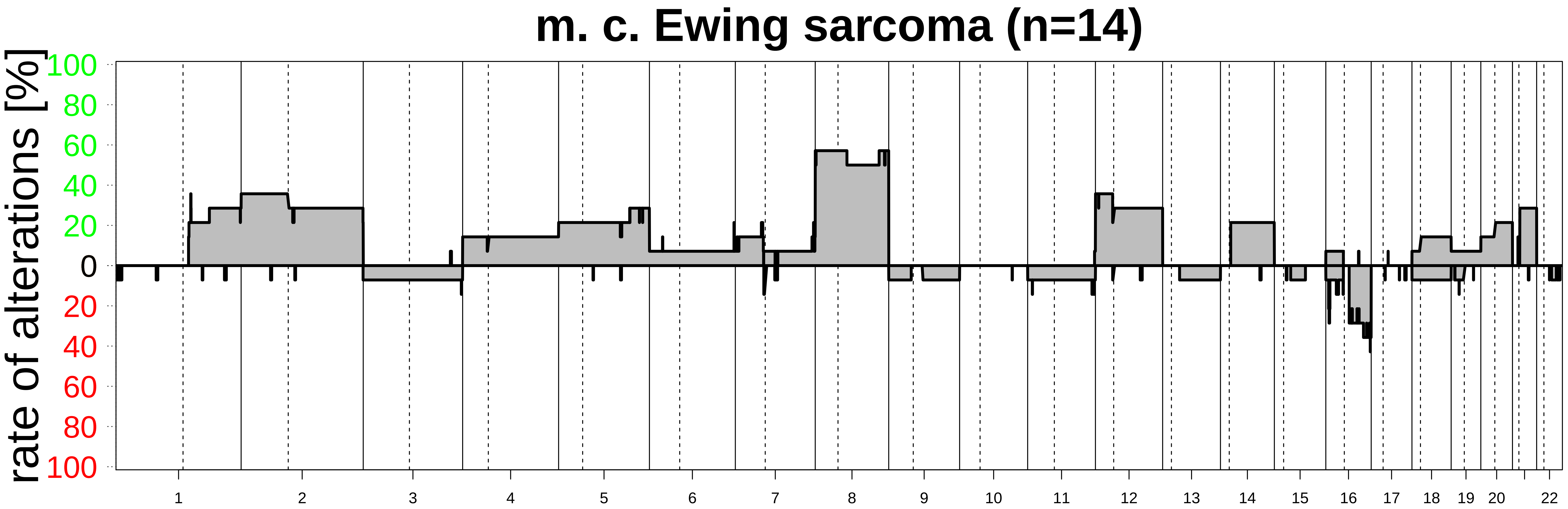
m. c. Ewing sarcoma (n=14)
rate of alterations [%]
100
80
60
40
20
0
20
40
60
80
100
1
2
3
4
5
6
7
8
9
10
11
12
13
14
15
16
17
18
19
20
22

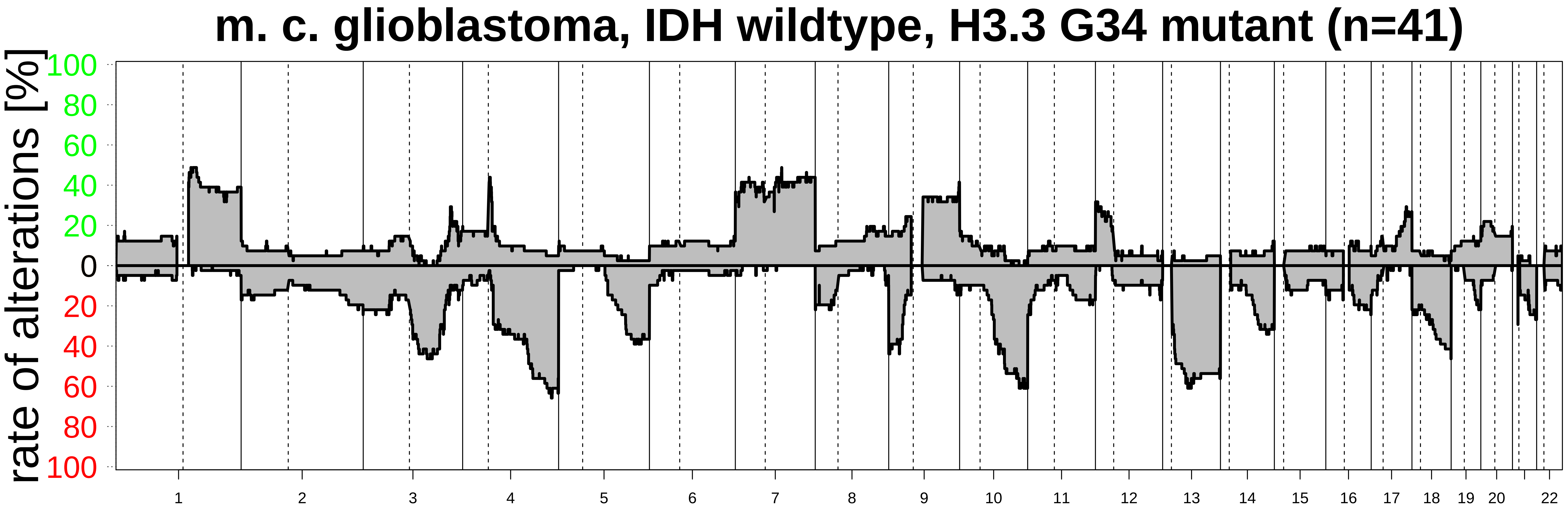

m. c. glioblastoma, IDH wildtype, H3.3 G34 mutant (n=41)
rate of alterations [%]
100
80
60
40
20
0
20
40
60
80
100
1
2
3
4
5
6
7
8
9
10
11
12
13
14
15
16
17
18
19
20
22

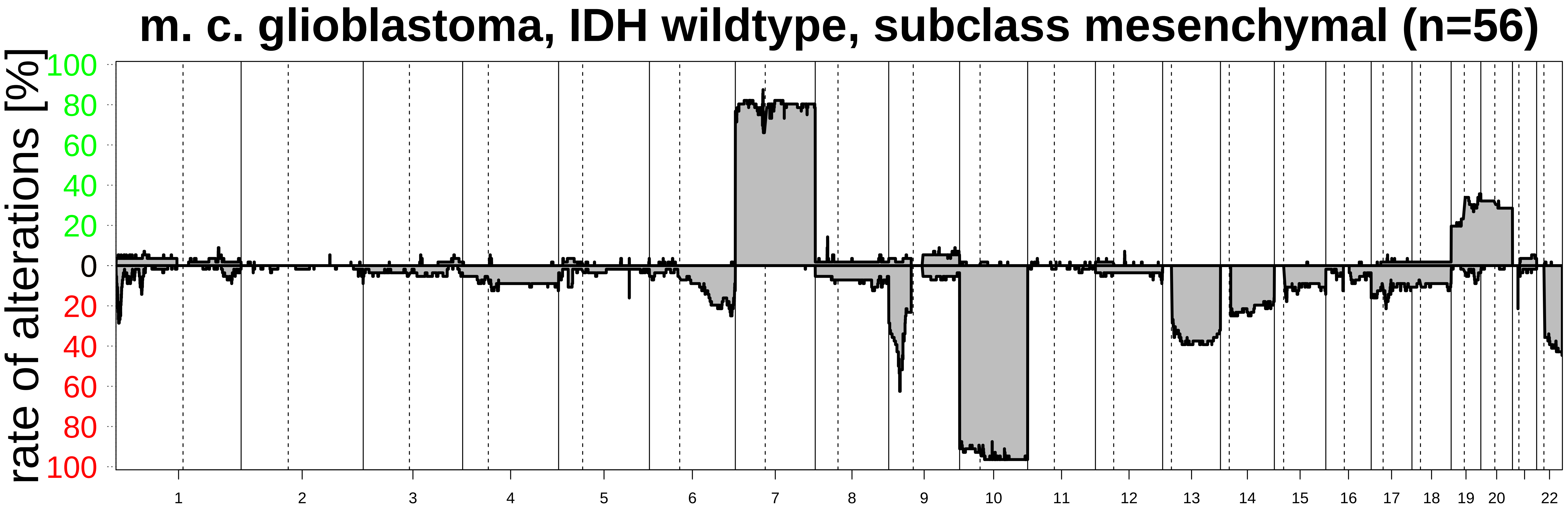
m. c. glioblastoma, IDH wildtype, subclass mesenchymal (n=56)
rate of alterations [%]
100
80
60
40
20
0
20
40
60
80
100
1
2
3
4
5
6
7
8
9
10
11
12
13
14
15
16
17
18
19
20
22

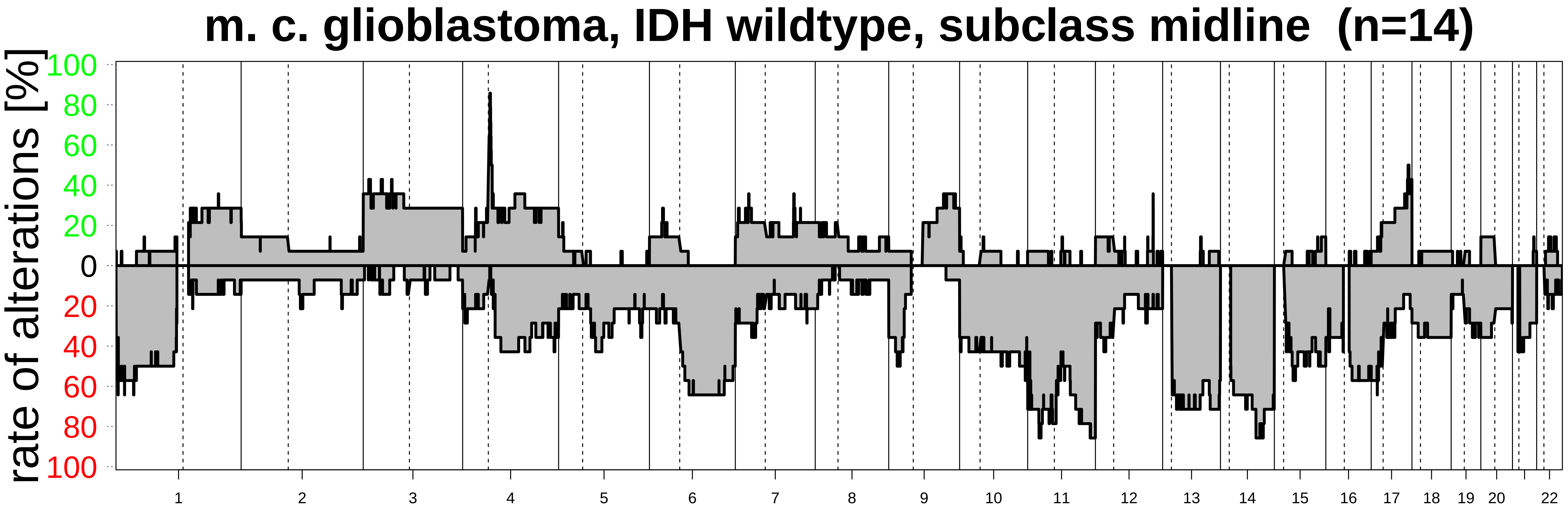

m. c. glioblastoma, IDH wildtype, subclass midline (n=14)
rate of alterations [%]
100
80
60
40
20
0
20
40
60
80
100
1
2
3
4
5
6
7
8
9
10
11
12
13
14
15
16
17
18
19
20
22

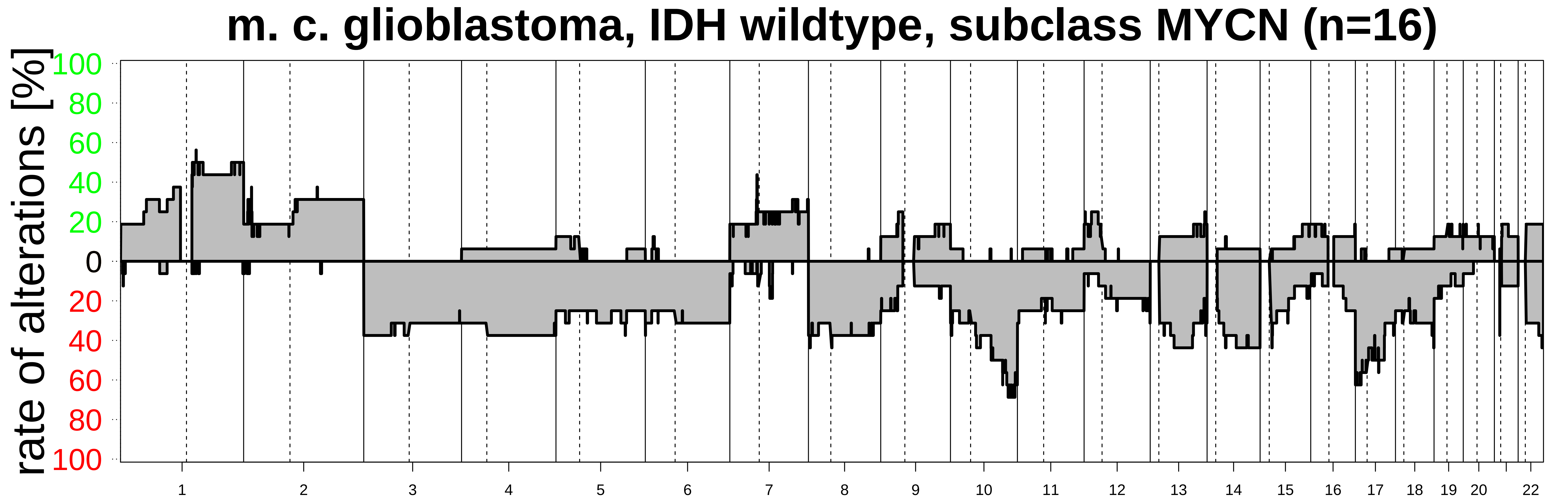
m. c. glioblastoma, IDH wildtype, subclass MYCN (n=16)
rate of alterations [%]
100
80
60
40
20
0
20
40
60
80
100
1
2
3
4
5
6
7
8
9
10
11
12
13
14
15
16
17
18
19
20
22

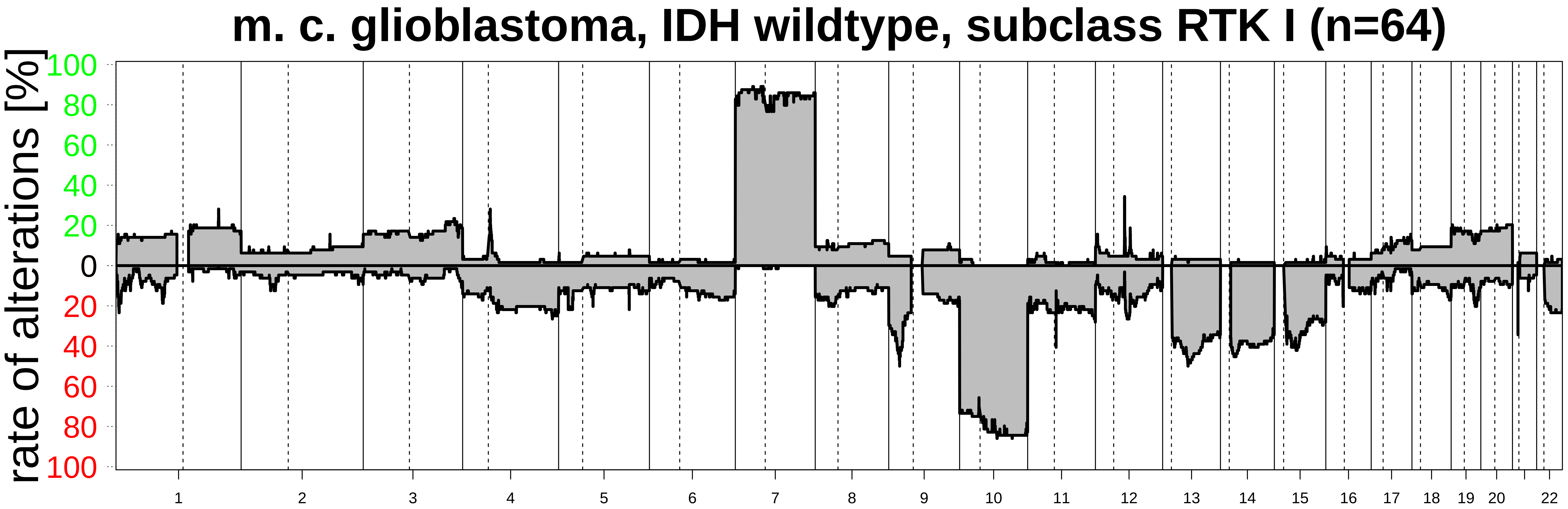

m. c. glioblastoma, IDH wildtype, subclass RTK I (n=64)
rate of alterations [%]
100
80
60
40
20
0
20
40
60
80
100
1
2
3
4
5
6
7
8
9
10
11
12
13
14
15
16
17
18
19
20
22

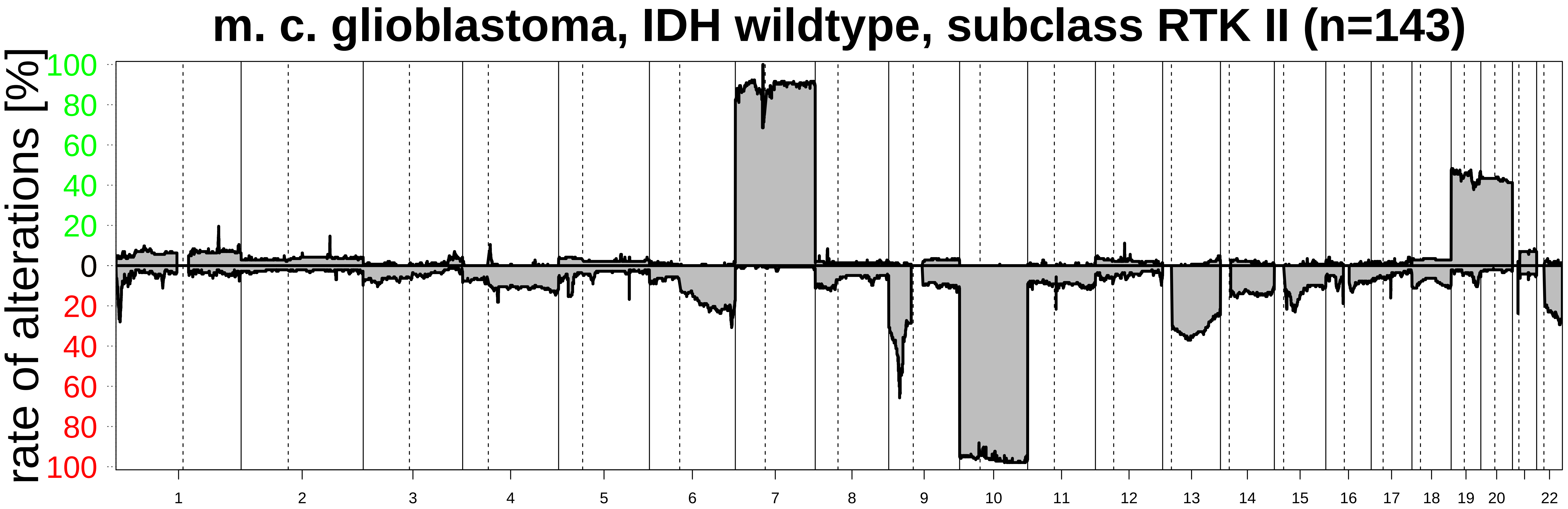

m. c. glioblastoma, IDH wildtype, subclass RTK II (n=143)
rate of alterations [%]
100
80
60
40
20
0
20
40
60
80
100
1
2
3
4
5
6
7
8
9
10
11
12
13
14
15
16
17
18
19
20
22

m. c. glioblastoma, IDH wildtype, subclass RTK III (n=13)

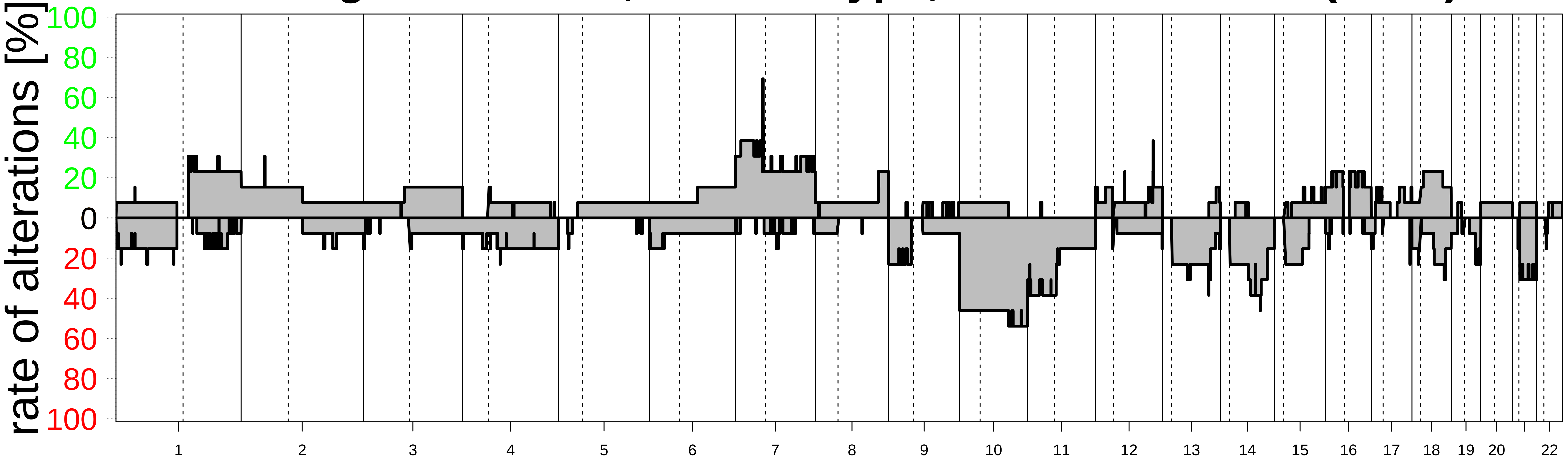

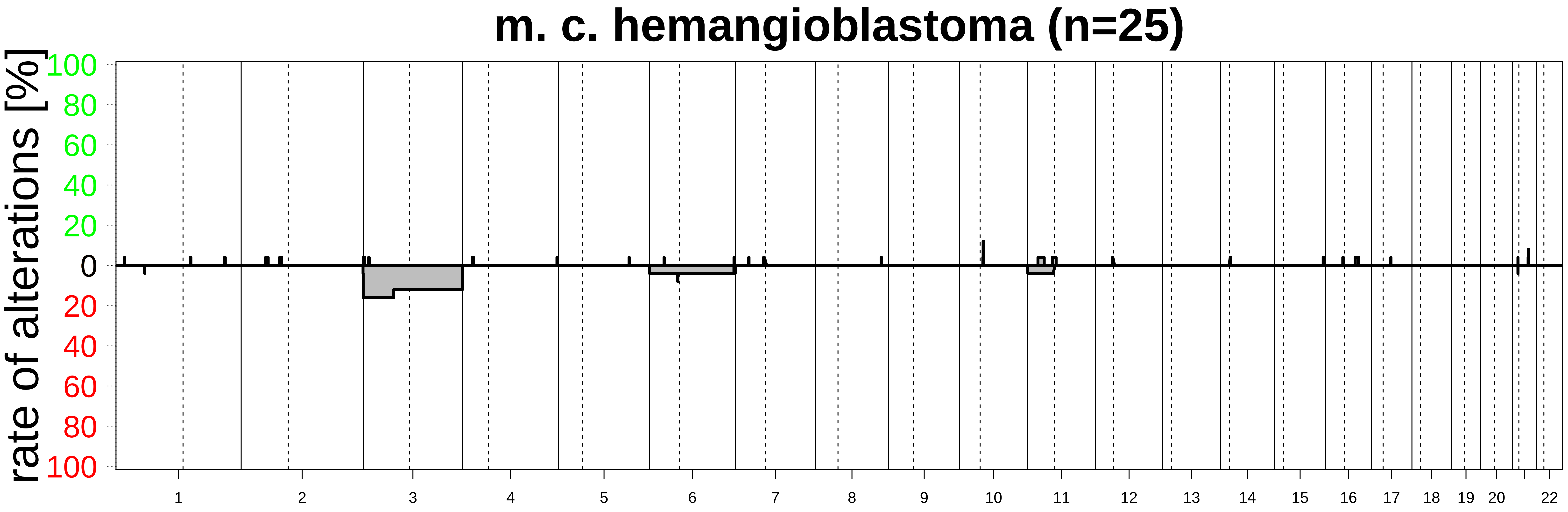

m. c. hemangioblastoma (n=25)
rate of alterations [%]
100
80
60
40
20
0
20
40
60
80
100
1
2
3
4
5
6
7
8
9
10
11
12
13
14
15
16
17
18
19
20
22

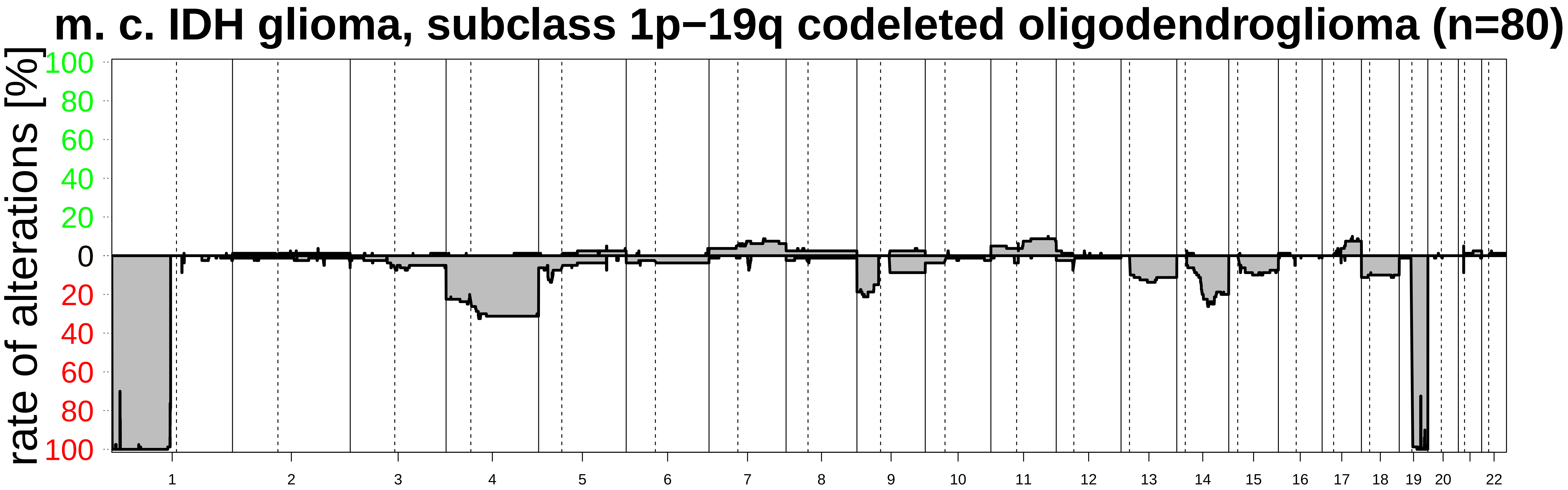

m. c. IDH glioma, subclass 1p−19q codeleted oligodendroglioma (n=80)
rate of alterations [%]
100
80
60
40
20
0
20
40
60
80
100
1
2
3
4
5
6
7
8
9
10
11
12
13
14
15
16
17
18
19
20
22

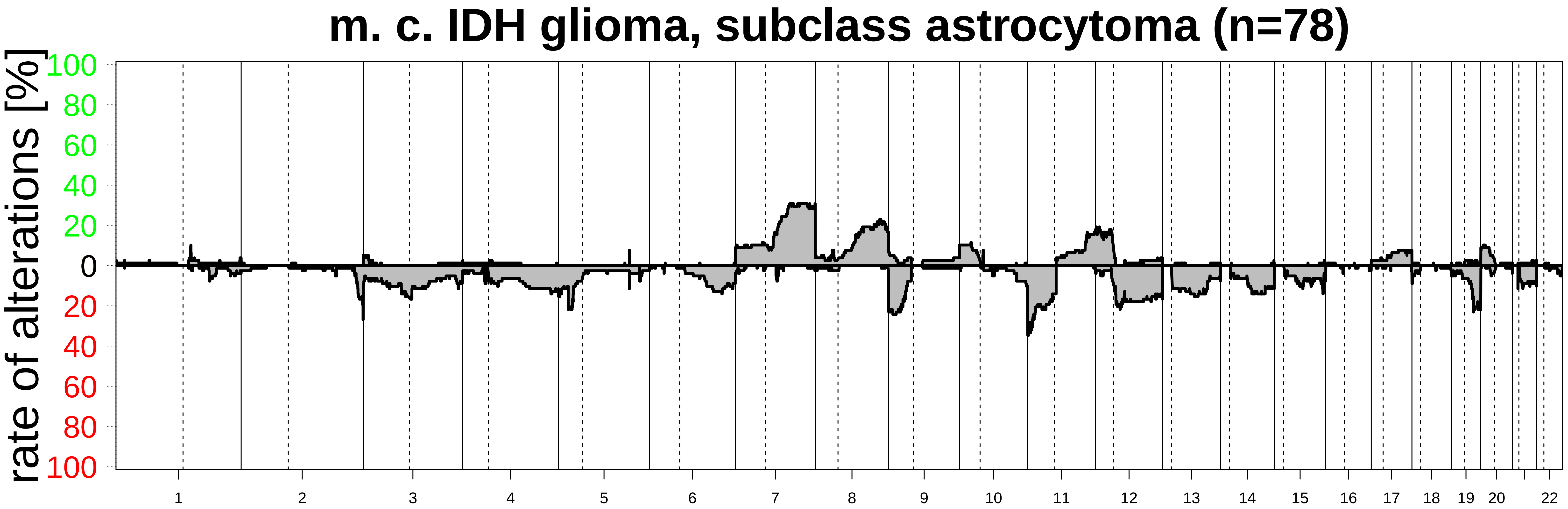
m. c. IDH glioma, subclass astrocytoma (n=78)
rate of alterations [%]
100
80
60
40
20
0
20
40
60
80
100
1
2
3
4
5
6
7
8
9
10
11
12
13
14
15
16
17
18
19
20
22

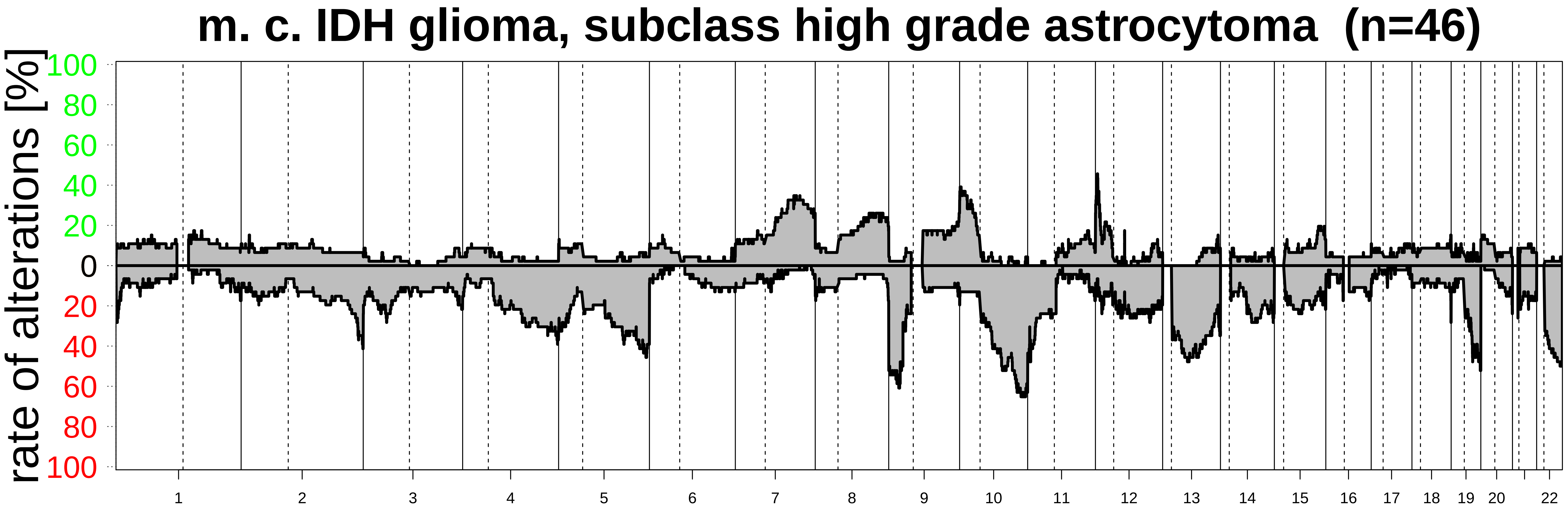
m. c. IDH glioma, subclass high grade astrocytoma (n=46)
rate of alterations [%]
100
80
60
40
20
0
20
40
60
80
100
1
2
3
4
5
6
7
8
9
10
11
12
13
14
15
16
17
18
19
20
22

m. c. infantile hemispheric glioma (n=10)

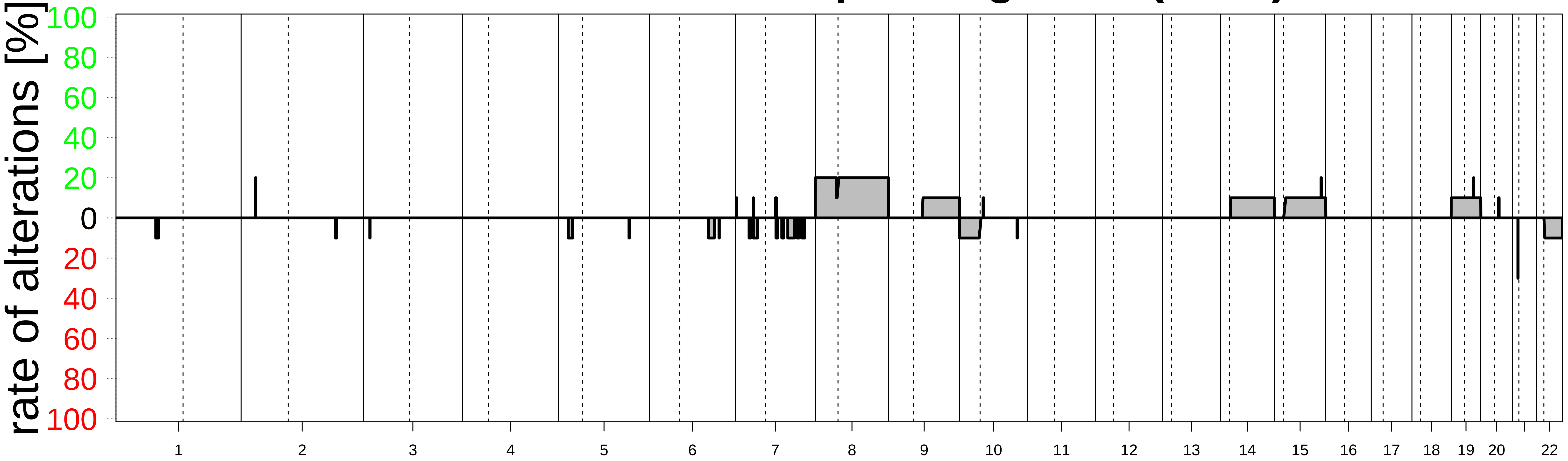

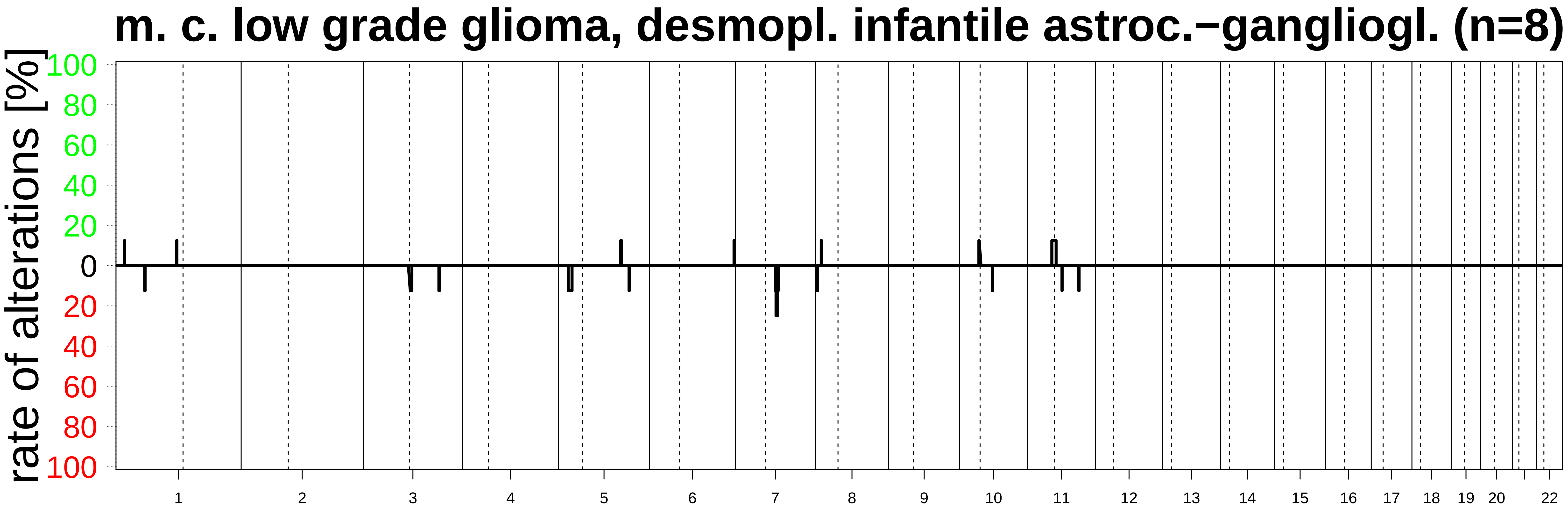
m. c. low grade glioma, desmopl. infantile astroc.−gangliogl. (n=8)
rate of alterations [%]
100
80
60
40
20
0
20
40
60
80
100
1
2
3
4
5
6
7
8
9
10
11
12
13
14
15
16
17
18
19
20
22

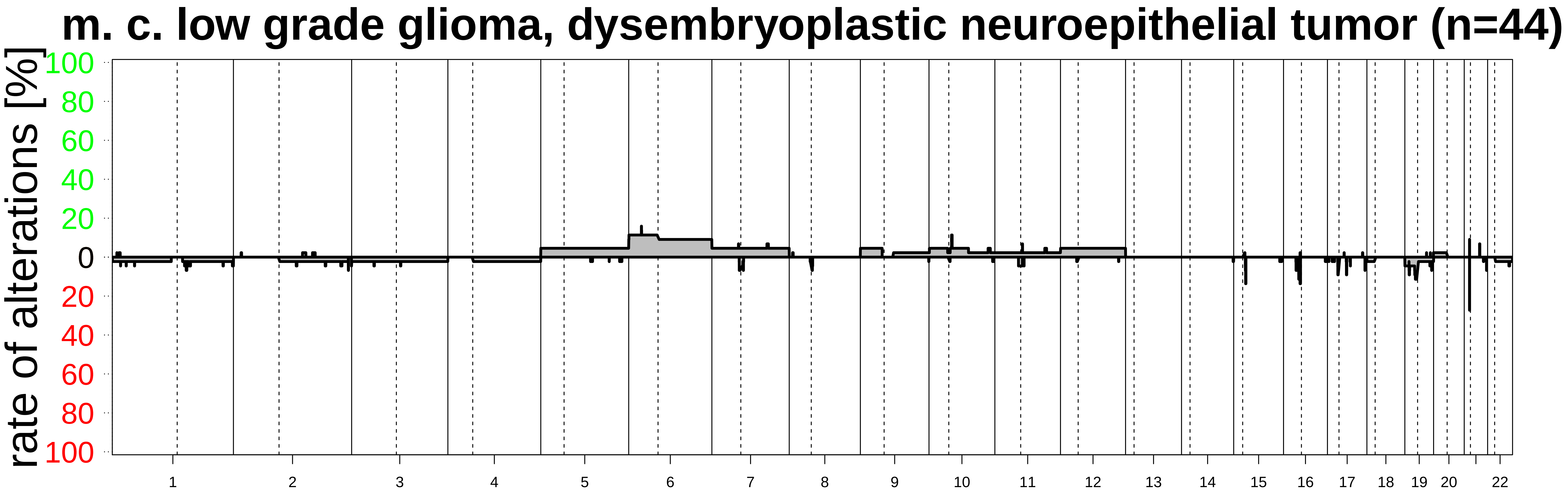
m. c. low grade glioma, dysembryoplastic neuroepithelial tumor (n=44)
rate of alterations [%]
100
80
60
40
20
0
20
40
60
80
100
1
2
3
4
5
6
7
8
9
10
11
12
13
14
15
16
17
18
19
20
22

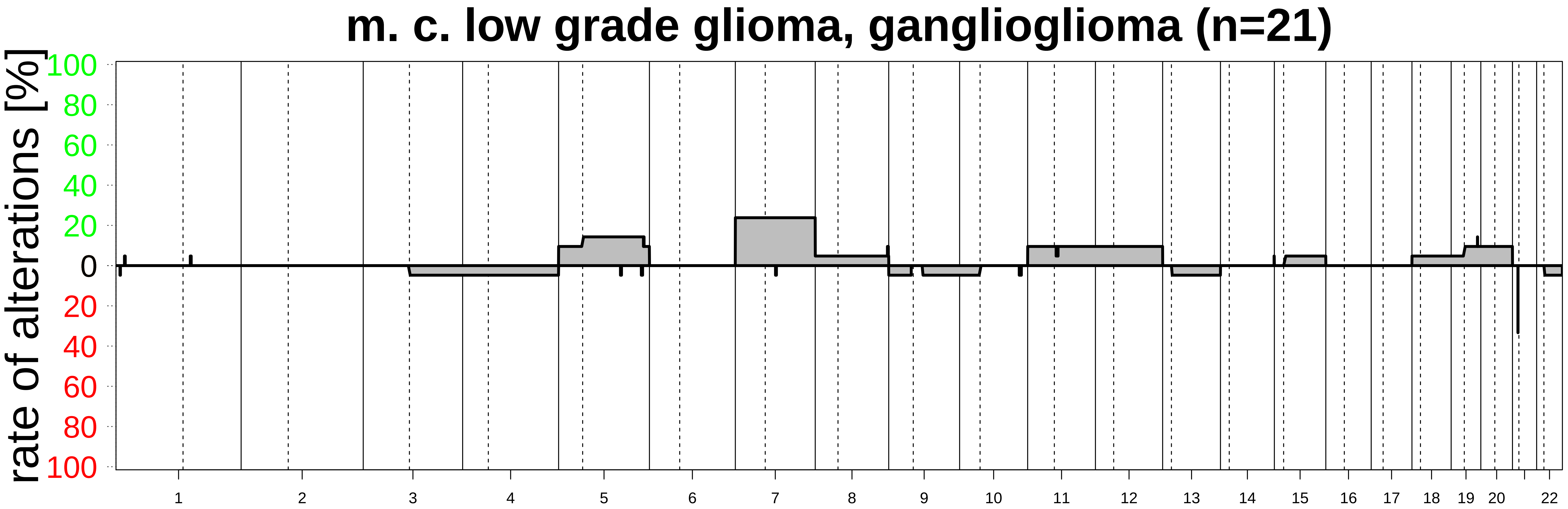
m. c. low grade glioma, ganglioglioma (n=21)
rate of alterations [%]
100
80
60
40
20
0
20
40
60
80
100
1
2
3
4
5
6
7
8
9
10
11
12
13
14
15
16
17
18
19
20
22

m. c. low grade glioma, MYB−MYBL1 (n=22)

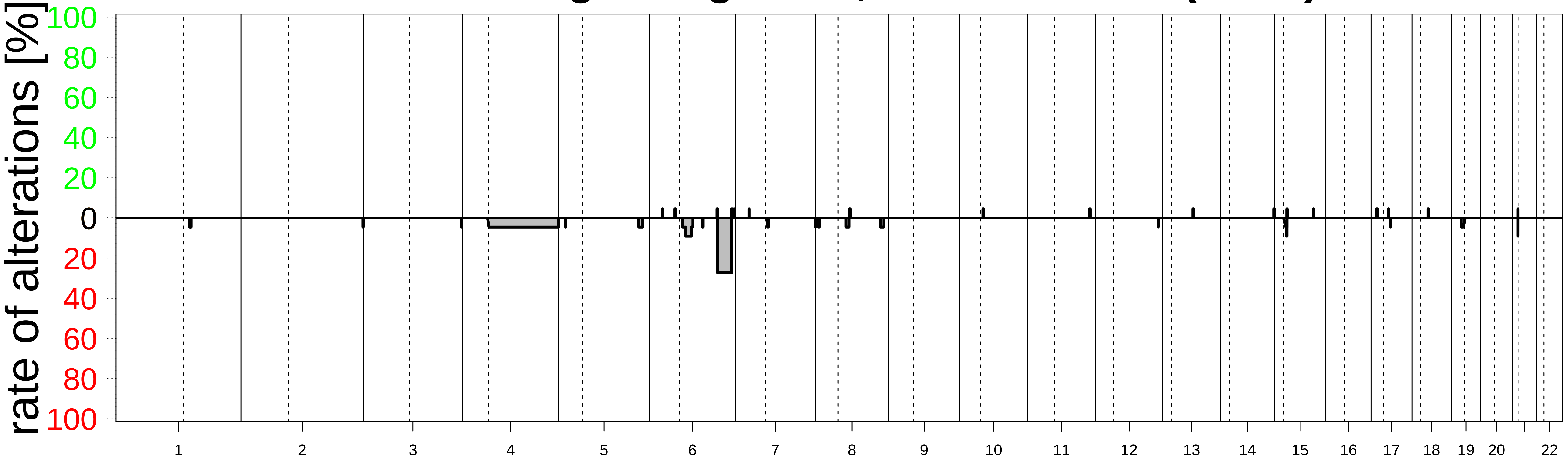

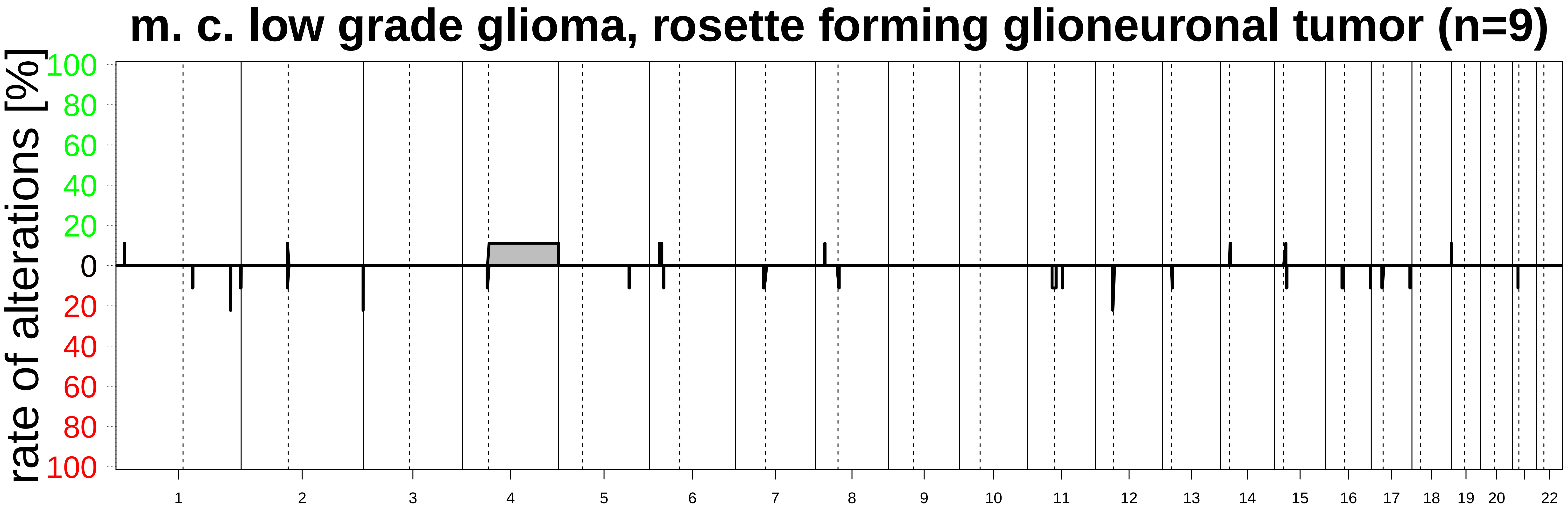
m. c. low grade glioma, rosette forming glioneuronal tumor (n=9)
rate of alterations [%]
100
80
60
40
20
0
20
40
60
80
100
1
2
3
4
5
6
7
8
9
10
11
12
13
14
15
16
17
18
19
20
22

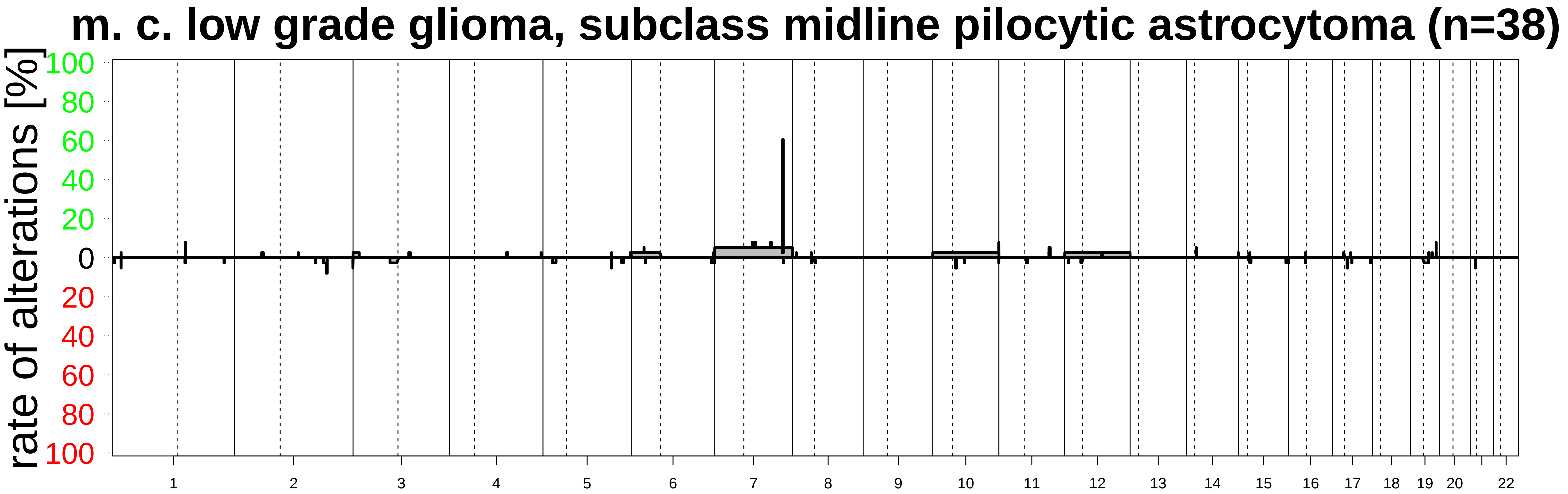

m. c. low grade glioma, subclass midline pilocytic astrocytoma (n=38)
rate of alterations [%]
100
80
60
40
20
0
20
40
60
80
100
1
2
3
4
5
6
7
8
9
10
11
12
13
14
15
16
17
18
19
20
22

m. c. low grade glioma, subcl. hemispheric piloc. astroc. − gangliogl. (n=2

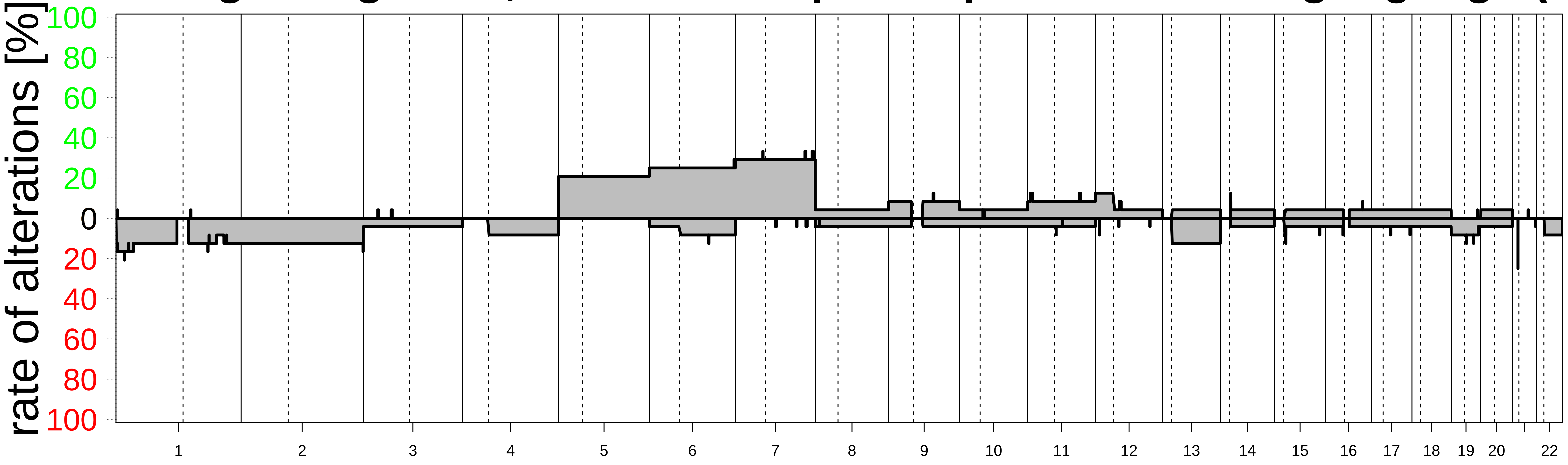

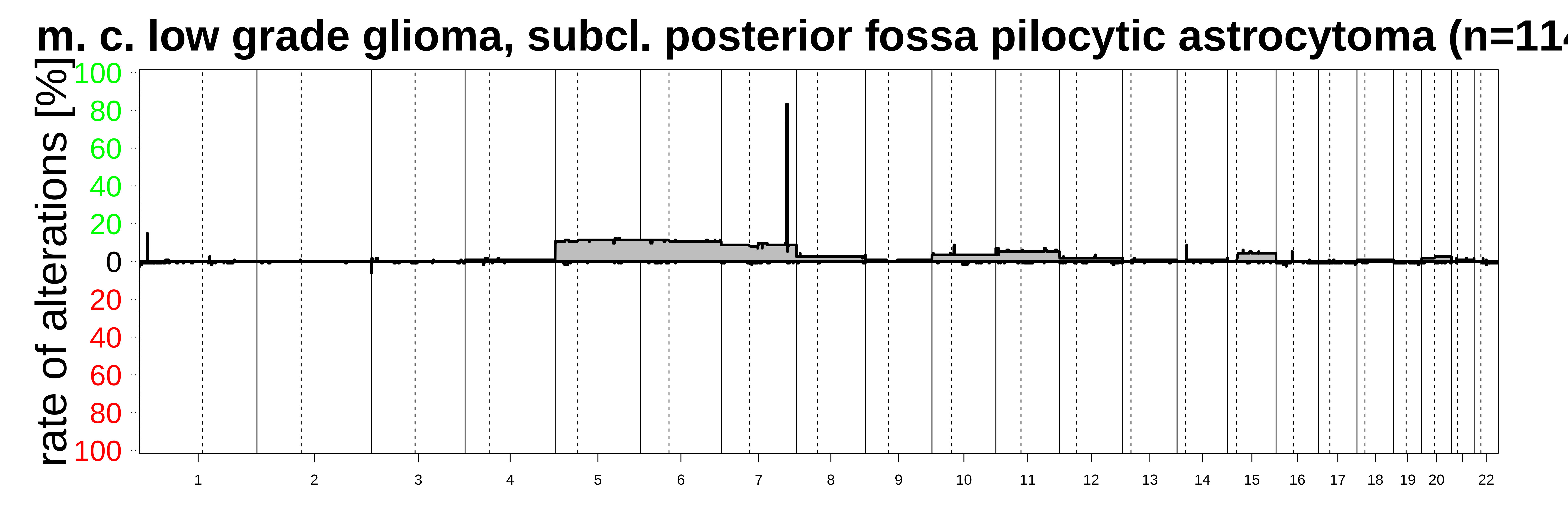

m. c. low grade glioma, subcl. posterior fossa pilocytic astrocytoma (n=114
rate of alterations [%]
100
80
60
40
20
0
20
40
60
80
100
1
2
3
4
5
6
7
8
9
10
11
12
13
14
15
16
17
18
19
20
22

m. c. low grade glioma, subependymal giant cell astrocytoma (n=21)

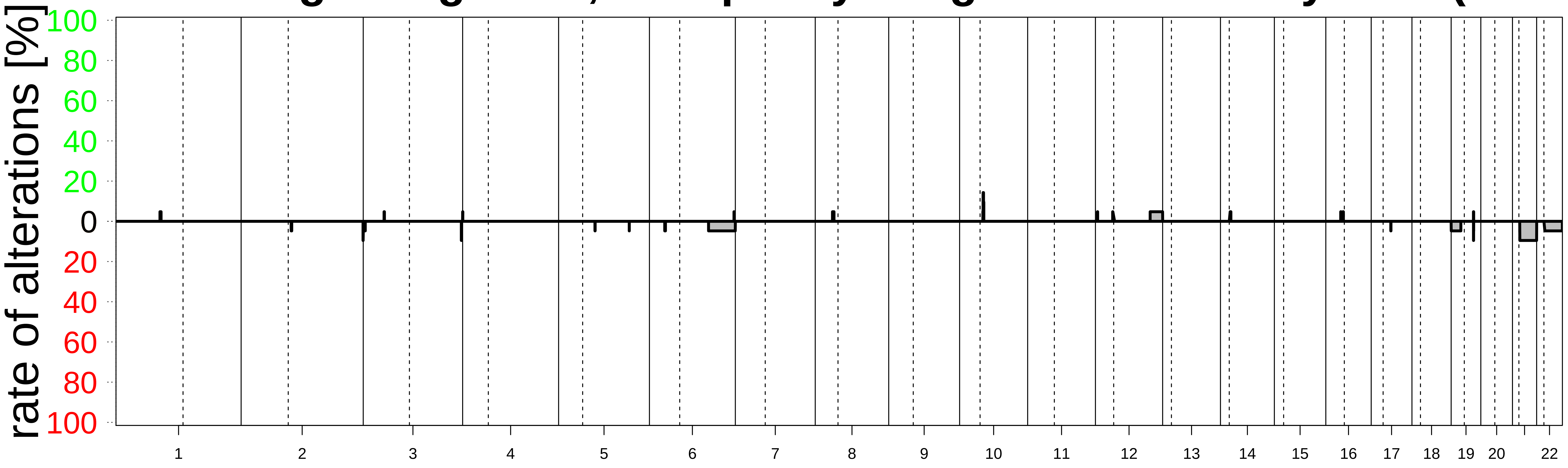

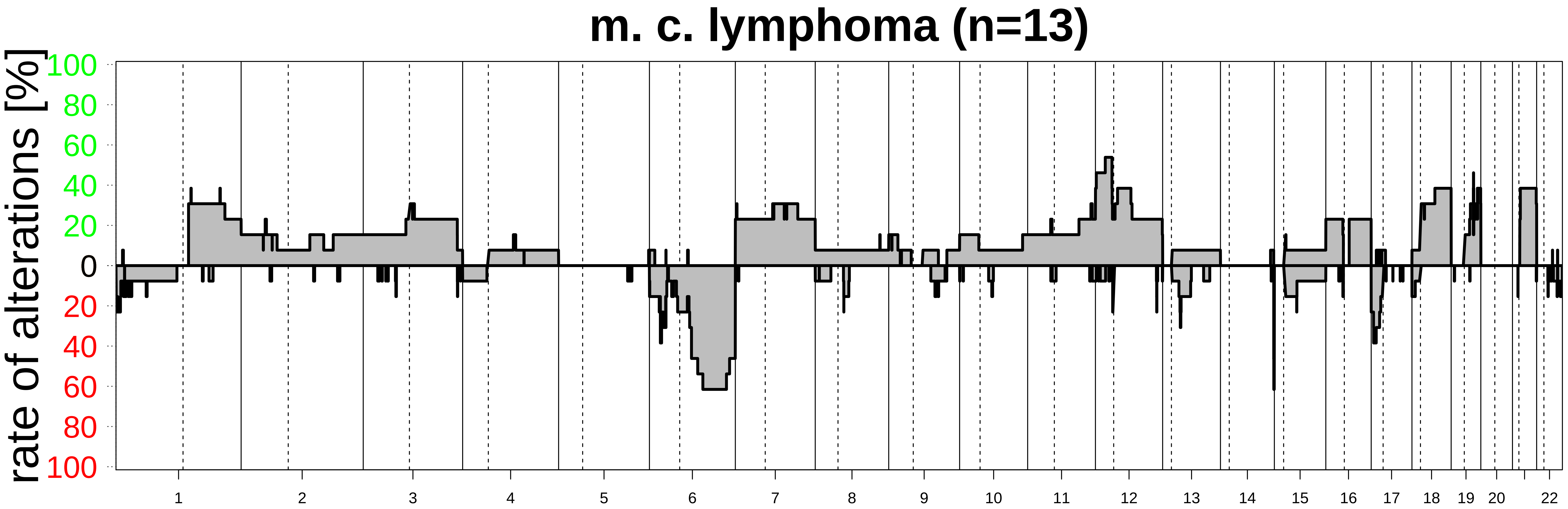
m. c. lymphoma (n=13)
rate of alterations [%]
100
80
60
40
20
0
20
40
60
80
100
1
2
3
4
5
6
7
8
9
10
11
12
13
14
15
16
17
18
19
20
22

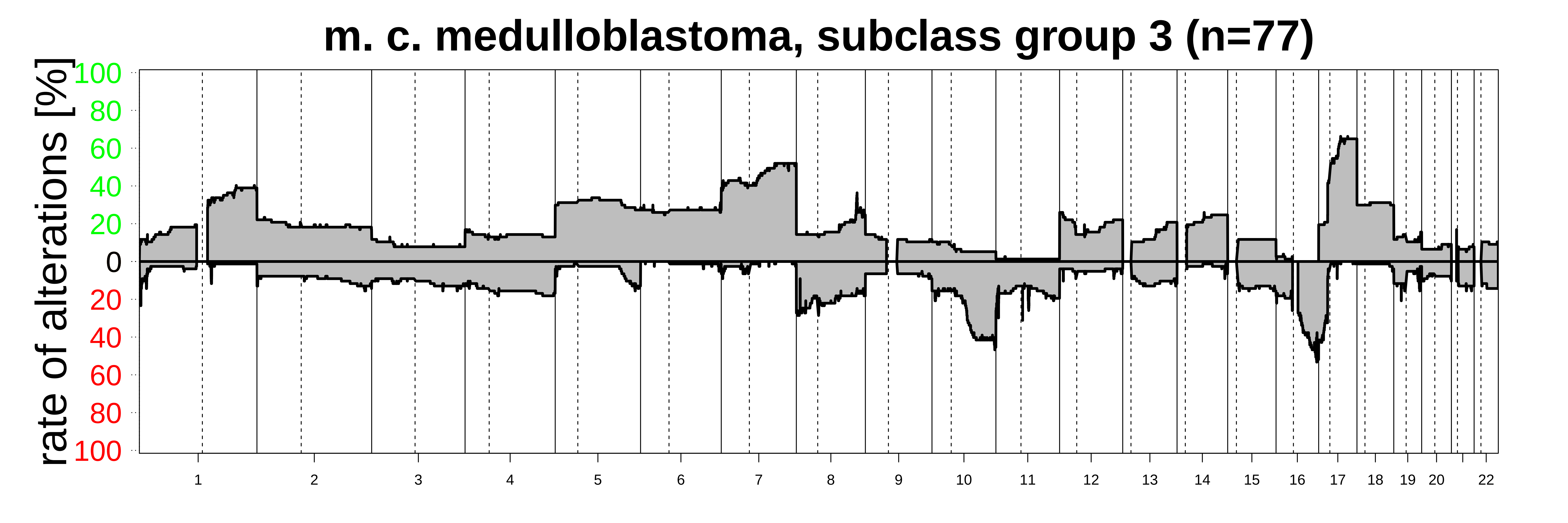
m. c. medulloblastoma, subclass group 3 (n=77)
rate of alterations [%]
100
80
60
40
20
0
20
40
60
80
100
1
2
3
4
5
6
7
8
9
10
11
12
13
14
15
16
17
18
19
20
22

m. c. medulloblastoma, subclass group 4 (n=138)

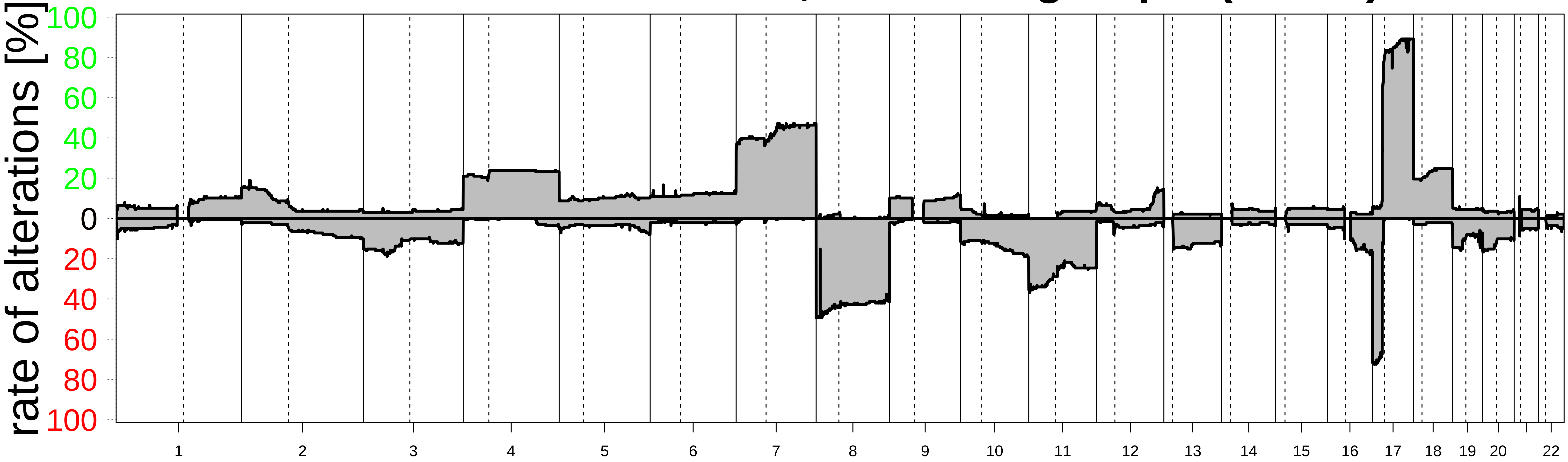

m. c. medulloblastoma, subclass SHH A (children and adult) (n=84)

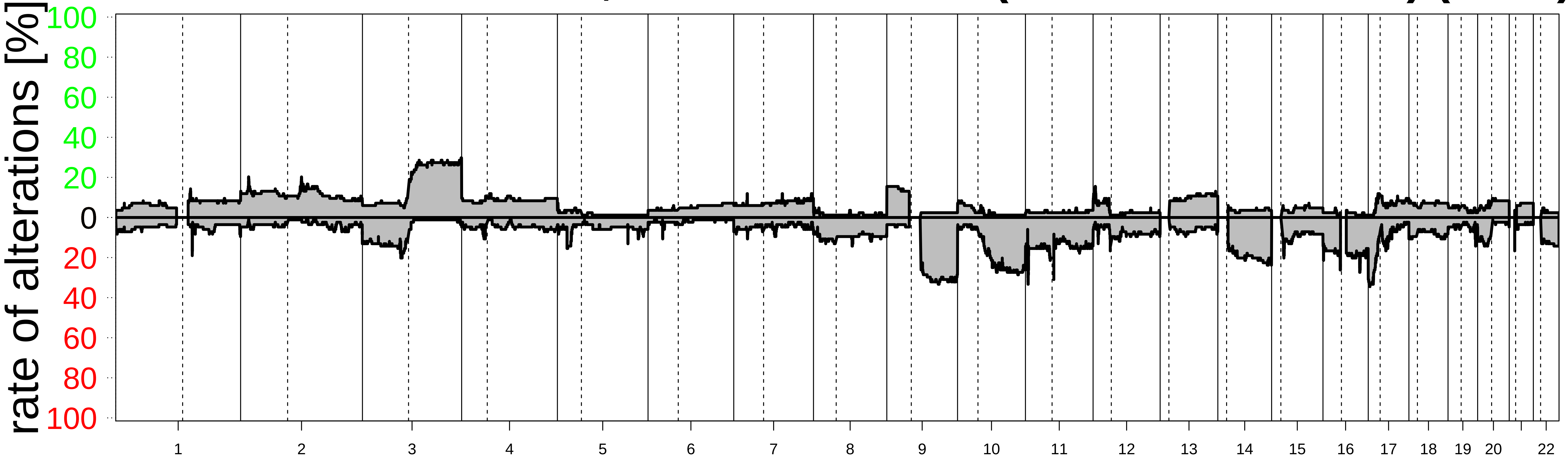

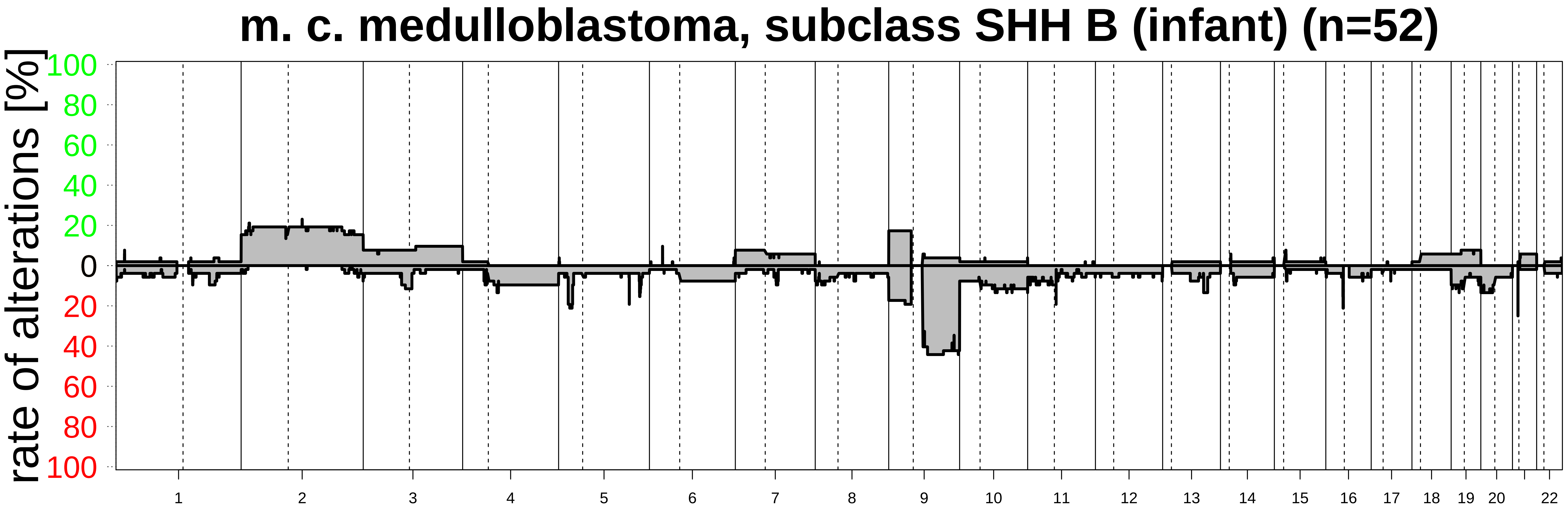
m. c. medulloblastoma, subclass SHH B (infant) (n=52)
rate of alterations [%]
100
80
60
40
20
0
20
40
60
80
100
1
2
3
4
5
6
7
8
9
10
11
12
13
14
15
16
17
18
19
20
22

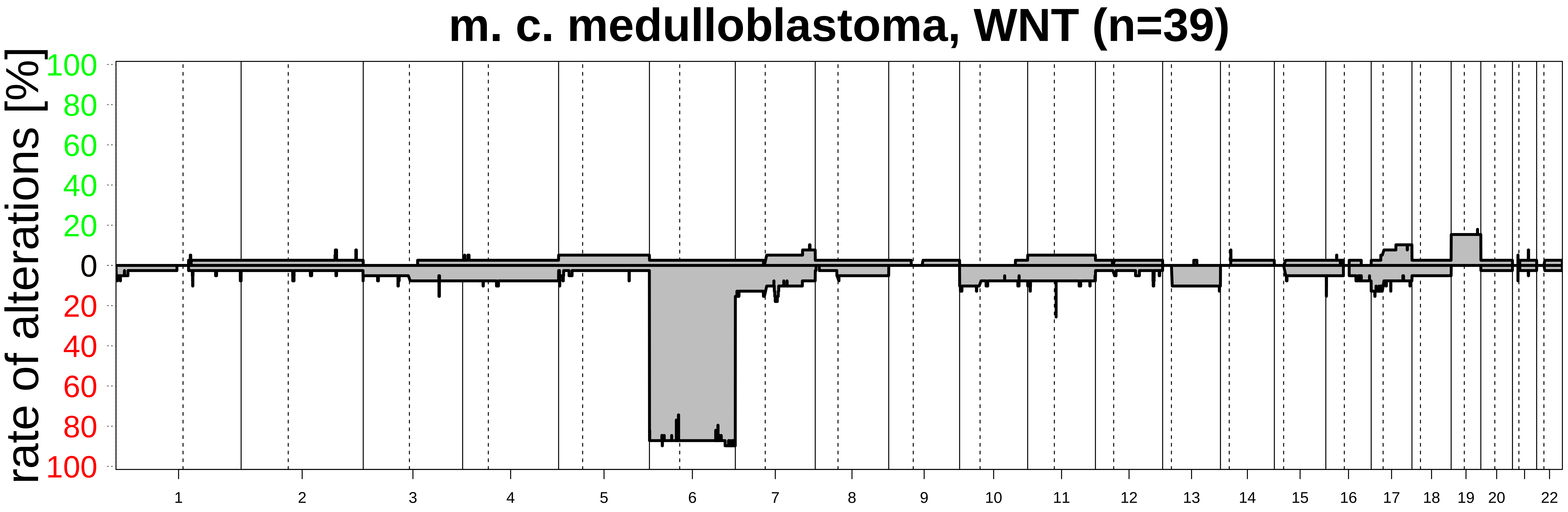
m. c. medulloblastoma, WNT (n=39)
rate of alterations [%]
100
80
60
40
20
0
20
40
60
80
100
1
2
3
4
5
6
7
8
9
10
11
12
13
14
15
16
17
18
19
20
22

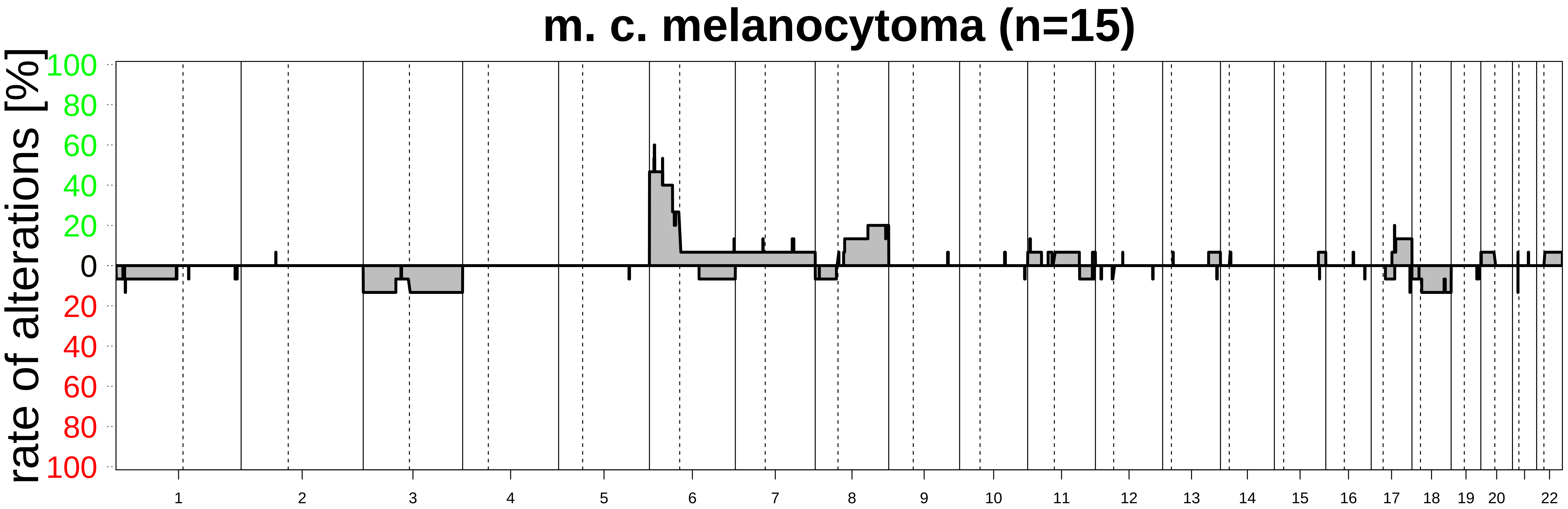
m. c. melanocytoma (n=15)
rate of alterations [%]
100
80
60
40
20
0
20
40
60
80
100
1
2
3
4
5
6
7
8
9
10
11
12
13
14
15
16
17
18
19
20
22

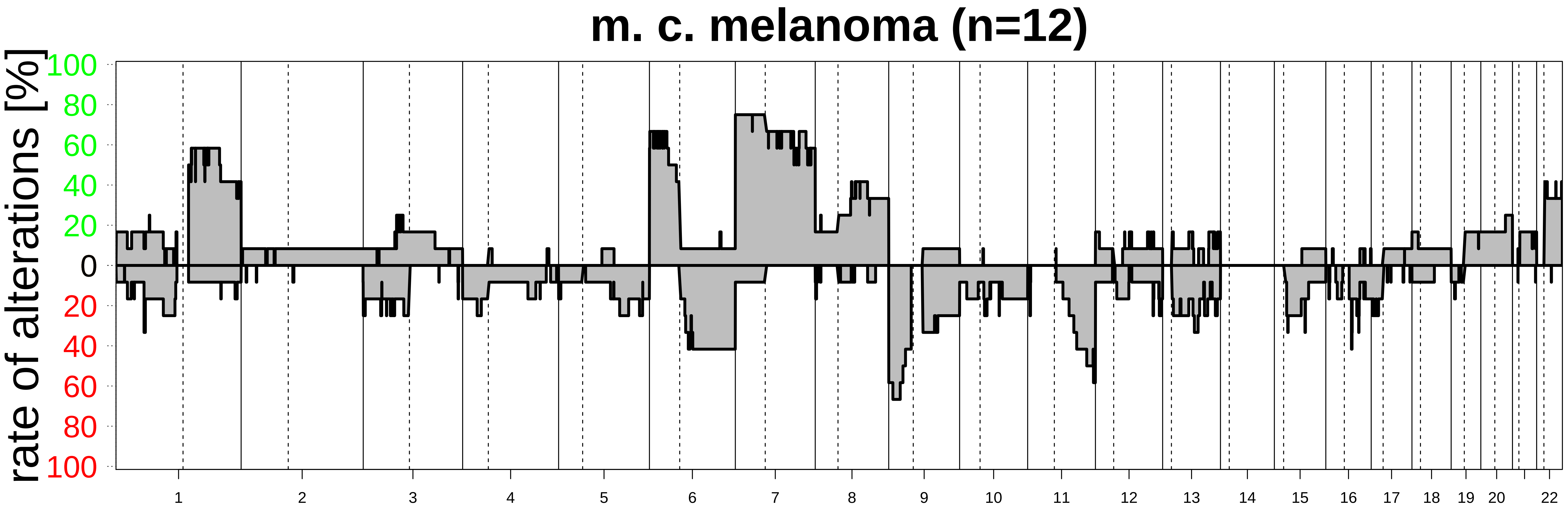

m. c. melanoma (n=12)
rate of alterations [%]
100
80
60
40
20
0
20
40
60
80
100
1
2
3
4
5
6
7
8
9
10
11
12
13
14
15
16
17
18
19
20
22

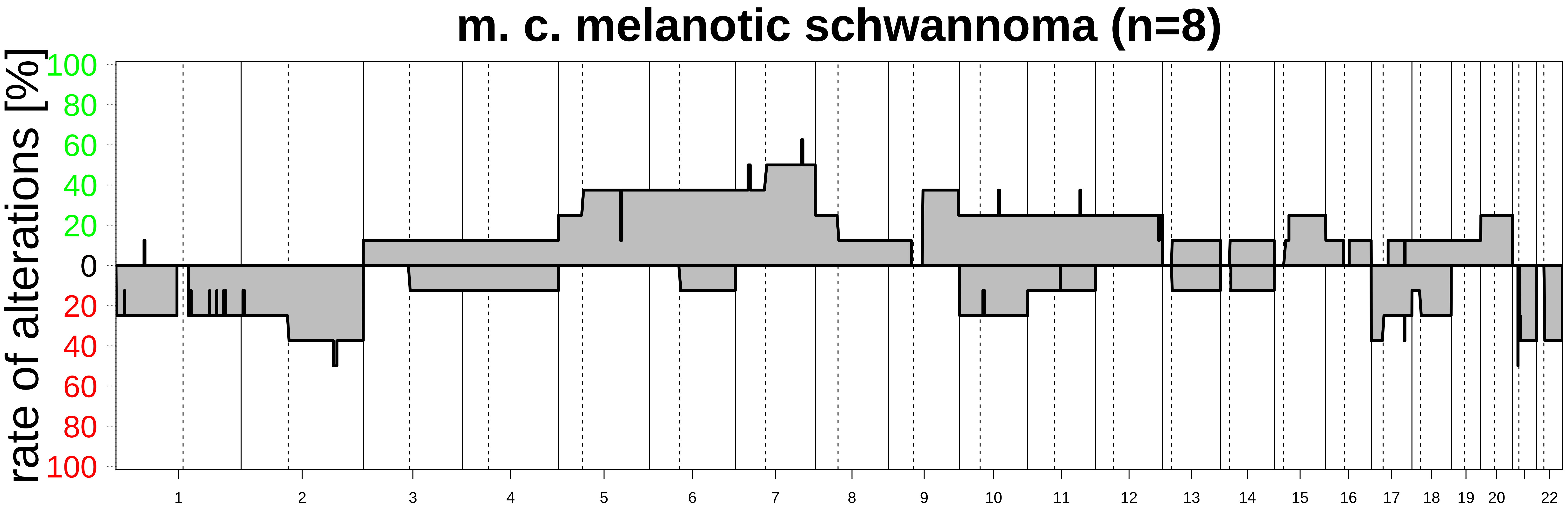

m. c. melanotic schwannoma (n=8)
rate of alterations [%]
100
80
60
40
20
0
20
40
60
80
100
1
2
3
4
5
6
7
8
9
10
11
12
13
14
15
16
17
18
19
20
22

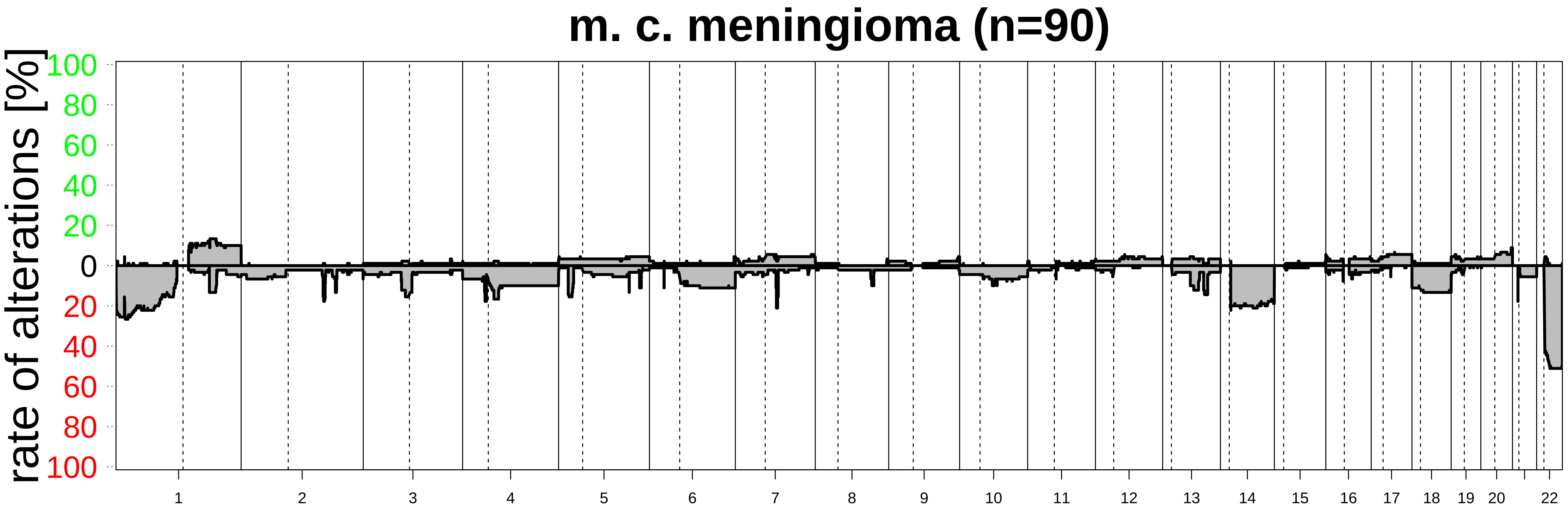
m. c. meningioma (n=90)
rate of alterations [%]
100
80
60
40
20
0
20
40
60
80
100
1
2
3
4
5
6
7
8
9
10
11
12
13
14
15
16
17
18
19
20
22

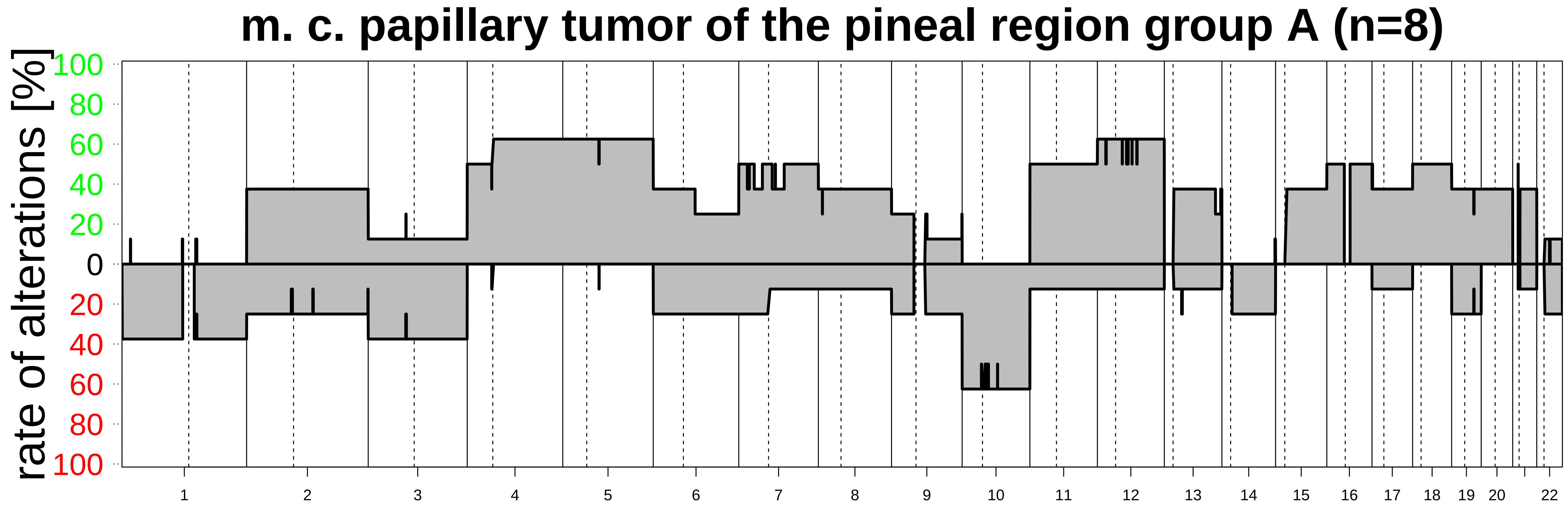
m. c. papillary tumor of the pineal region group A (n=8)
rate of alterations [%]
100
80
60
40
20
0
20
40
60
80
100
1
2
3
4
5
6
7
8
9
10
11
12
13
14
15
16
17
18
19
20
22

m. c. papillary tumor of the pineal region group B (n=22)

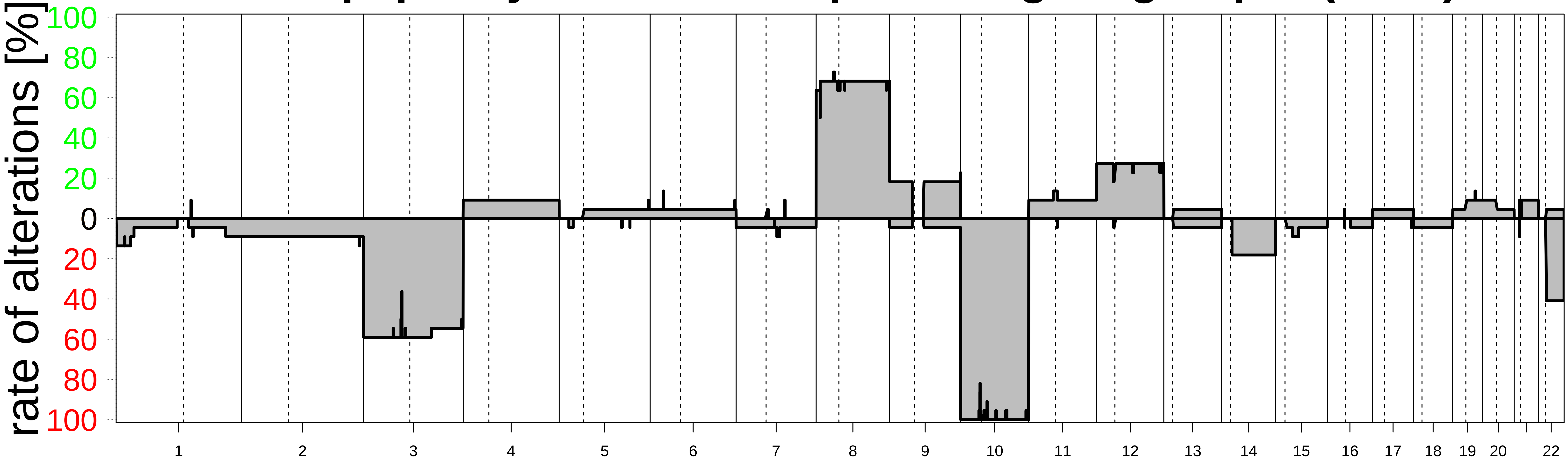

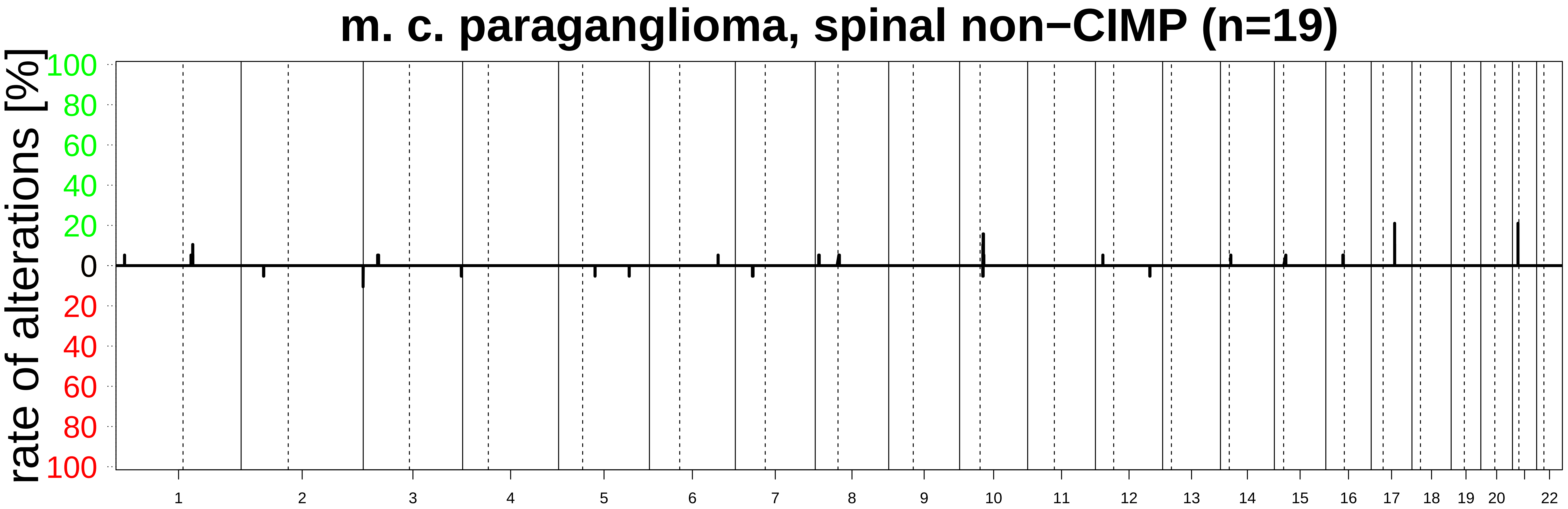

m. c. paraganglioma, spinal non−CIMP (n=19)
rate of alterations [%]
100
80
60
40
20
0
20
40
60
80
100
1
2
3
4
5
6
7
8
9
10
11
12
13
14
15
16
17
18
19
20
22

m. c. pineal parenchymal tumor (n=19)

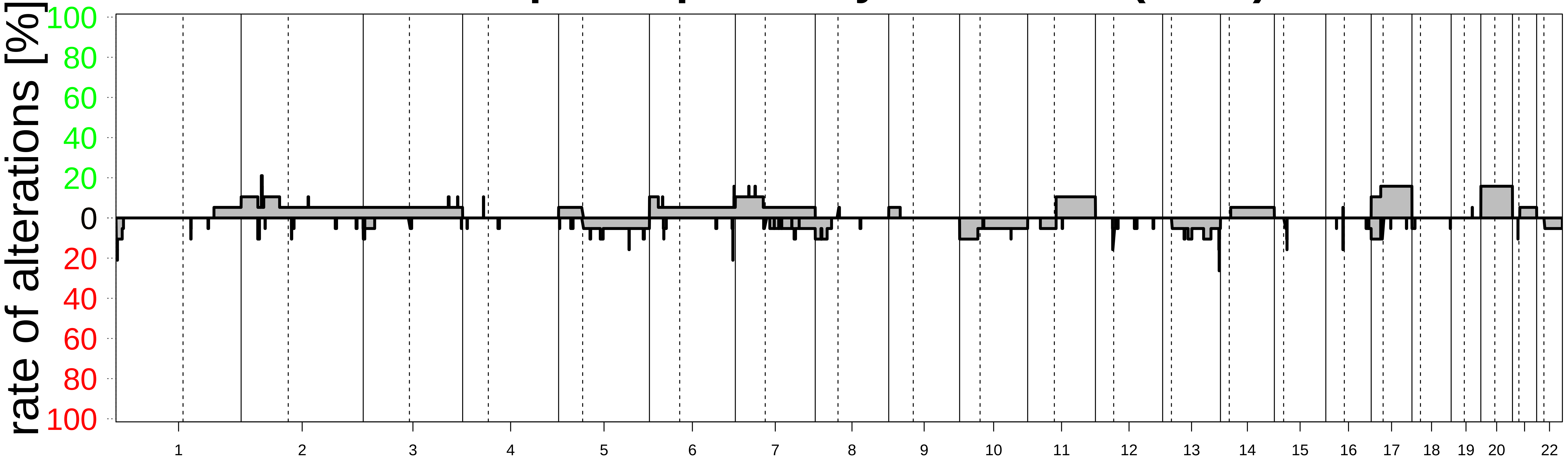

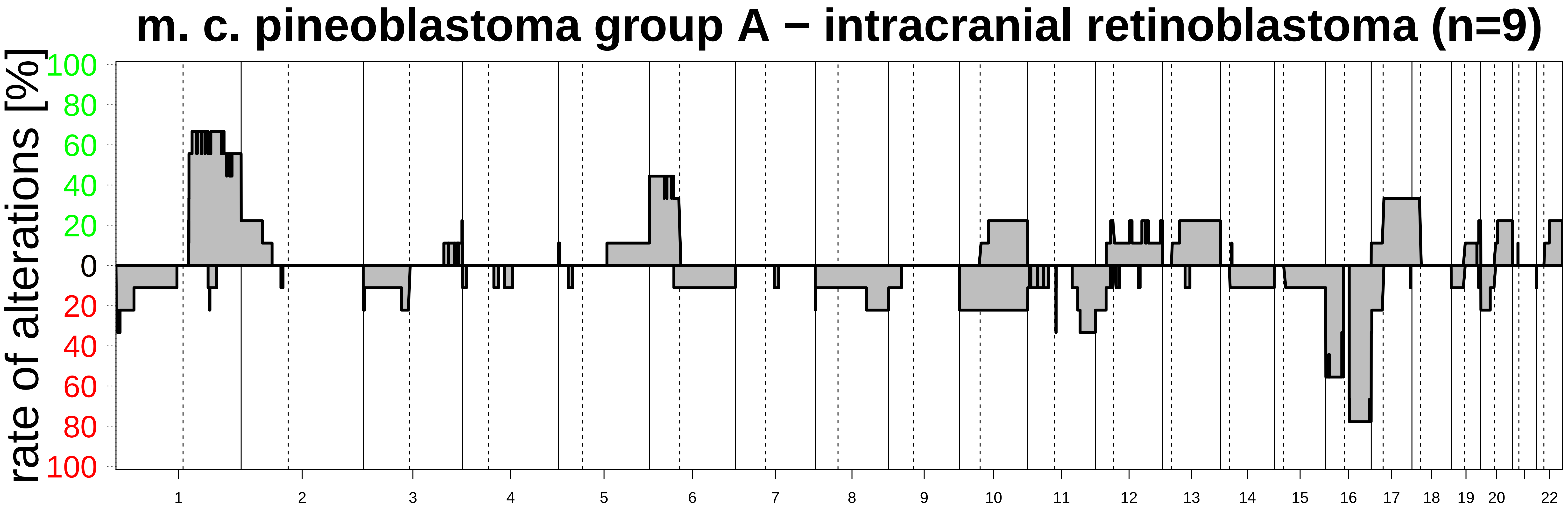
m. c. pineoblastoma group A − intracranial retinoblastoma (n=9)
rate of alterations [%]
100
80
60
40
20
0
20
40
60
80
100
1
2
3
4
5
6
7
8
9
10
11
12
13
14
15
16
17
18
19
20
22

m. c. pineoblastoma group B (n=22)

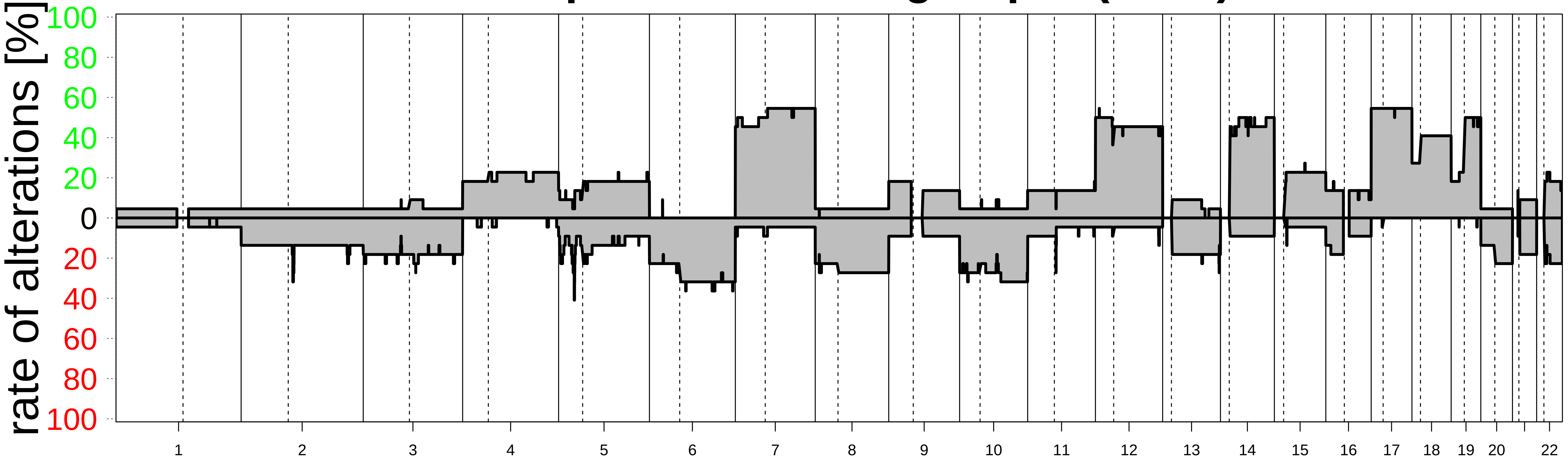

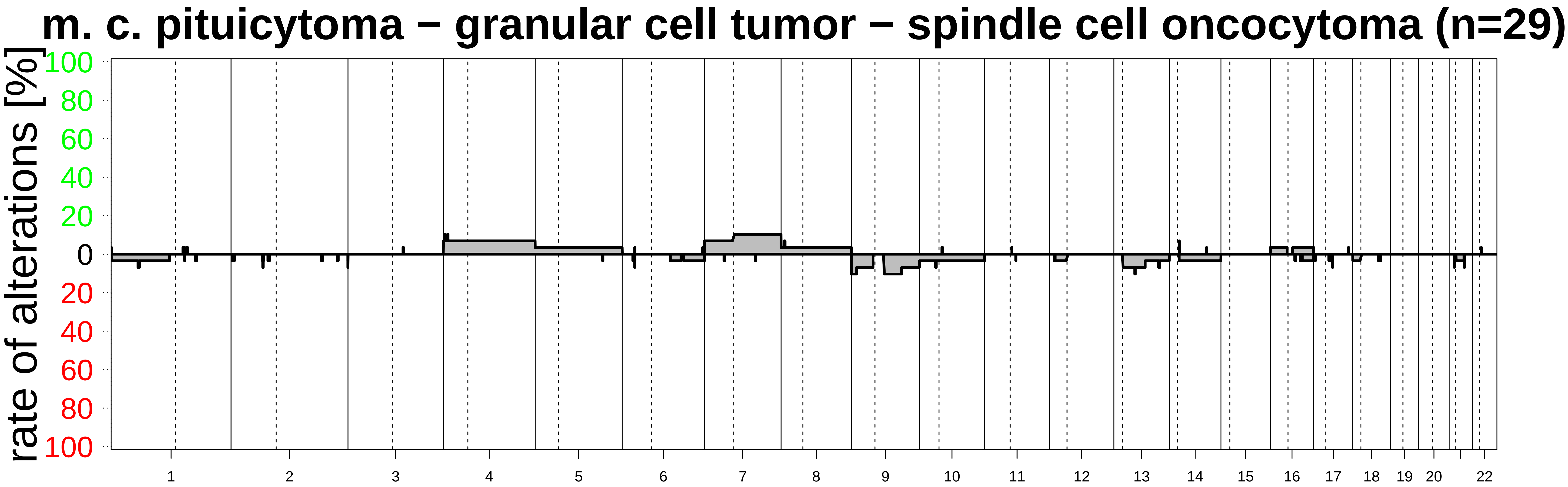
m. c. pituicytoma − granular cell tumor − spindle cell oncocytoma (n=29)
rate of alterations [%]
100
80
60
40
20
0
20
40
60
80
100
1
2
3
4
5
6
7
8
9
10
11
12
13
14
15
16
17
18
19
20
22

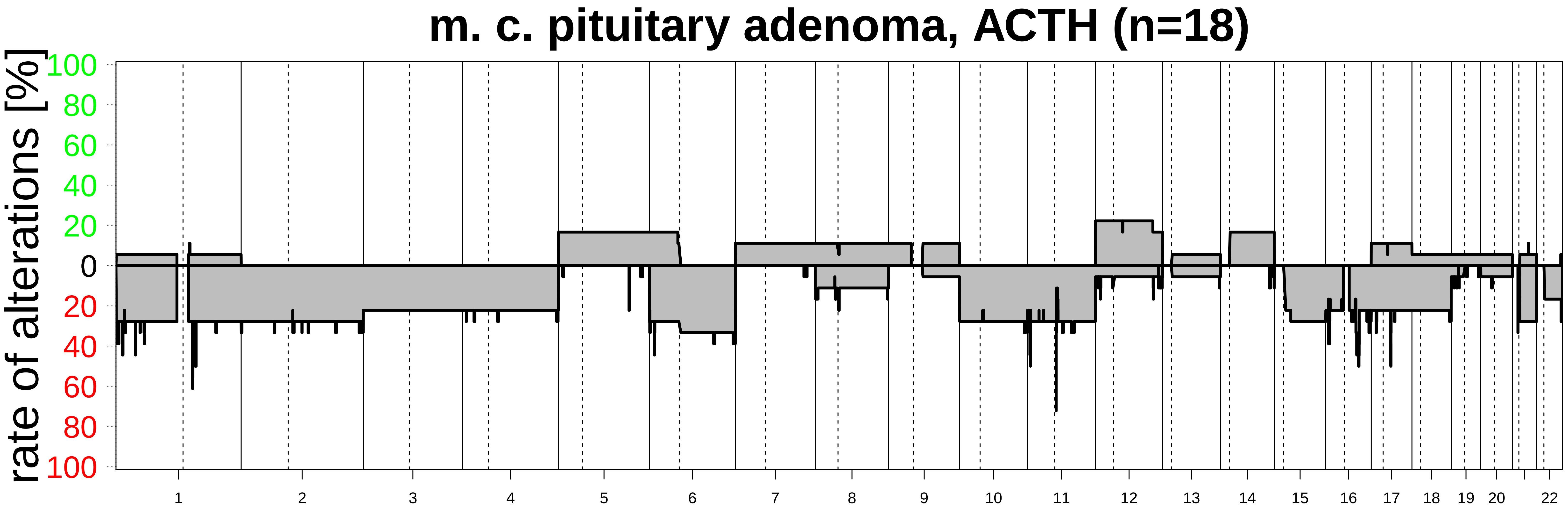

m. c. pituitary adenoma, ACTH (n=18)
rate of alterations [%]
100
80
60
40
20
0
20
40
60
80
100
1
2
3
4
5
6
7
8
9
10
11
12
13
14
15
16
17
18
19
20
22

m. c. pituitary adenoma, FSH−LH (n=21)

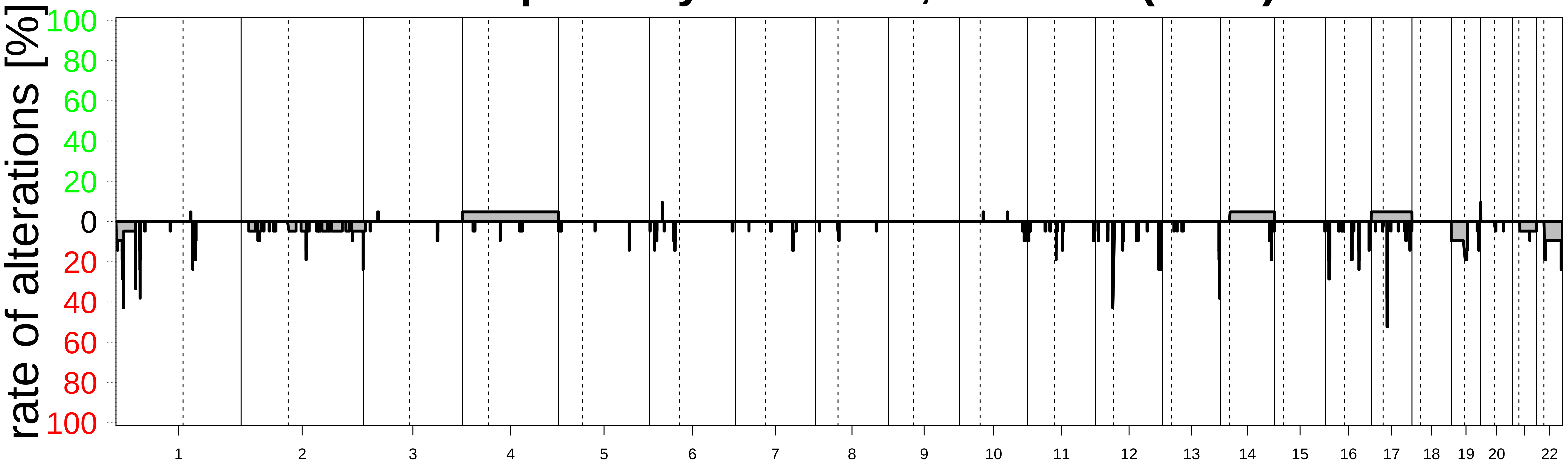

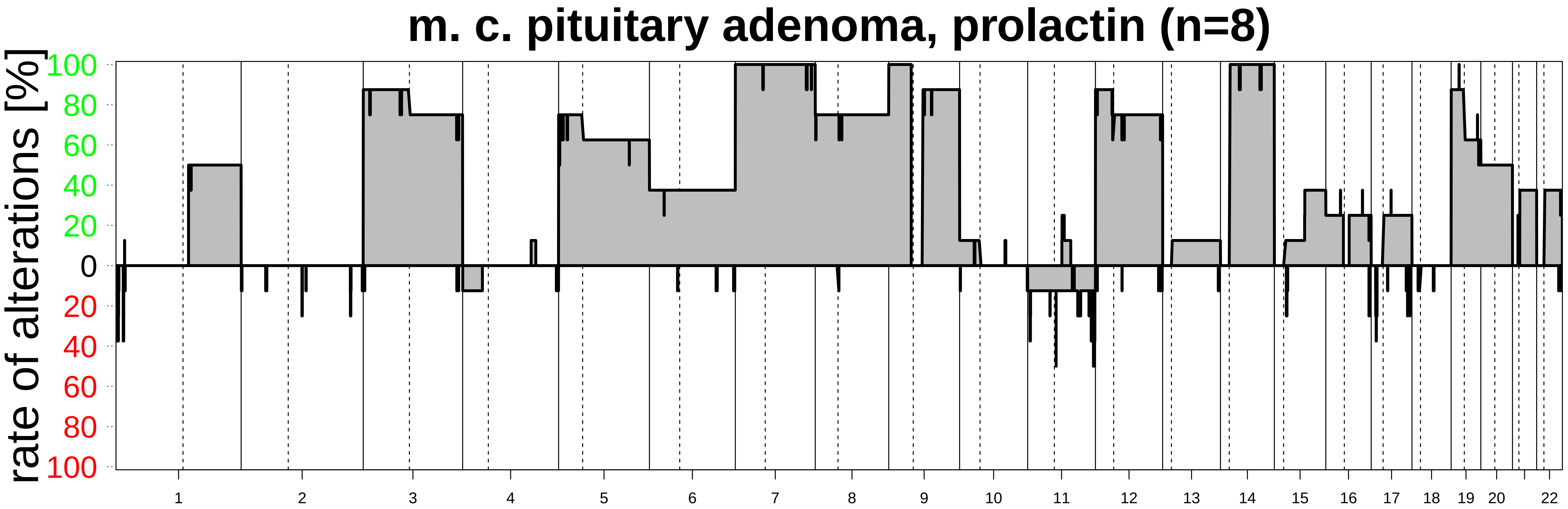
m. c. pituitary adenoma, prolactin (n=8)
rate of alterations [%]
100
80
60
40
20
0
20
40
60
80
100
1
2
3
4
5
6
7
8
9
10
11
12
13
14
15
16
17
18
19
20
22

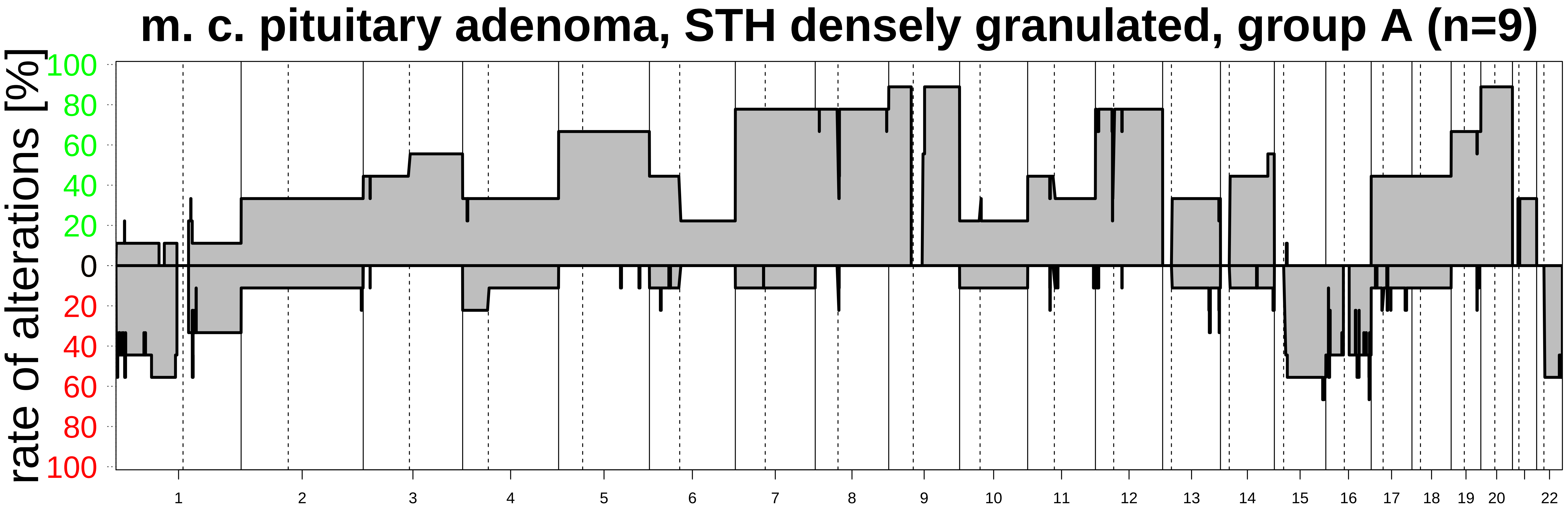
m. c. pituitary adenoma, STH densely granulated, group A (n=9)
rate of alterations [%]
100
80
60
40
20
0
20
40
60
80
100
1
2
3
4
5
6
7
8
9
10
11
12
13
14
15
16
17
18
19
20
22

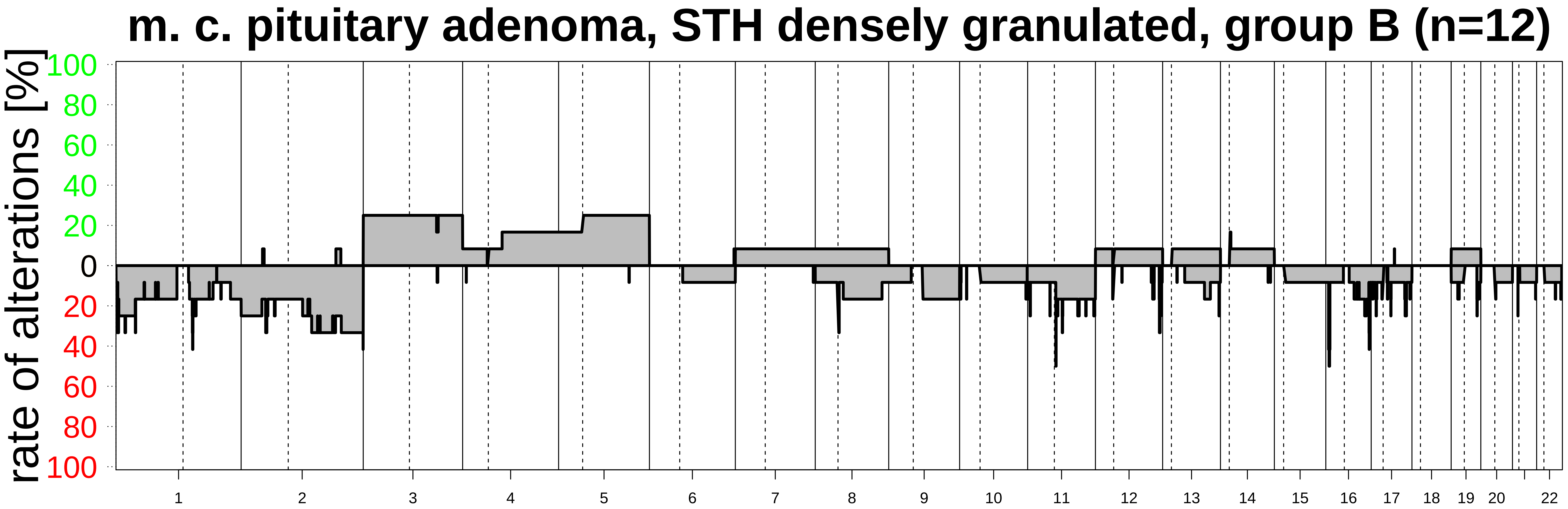
m. c. pituitary adenoma, STH densely granulated, group B (n=12)
rate of alterations [%]
100
80
60
40
20
0
20
40
60
80
100
1 2 3 4 5 6 7 8 9 10 11 12 13 14 15 16 17 18 19 20 22

m. c. pituitary adenoma, STH sparsely granulated (n=17)

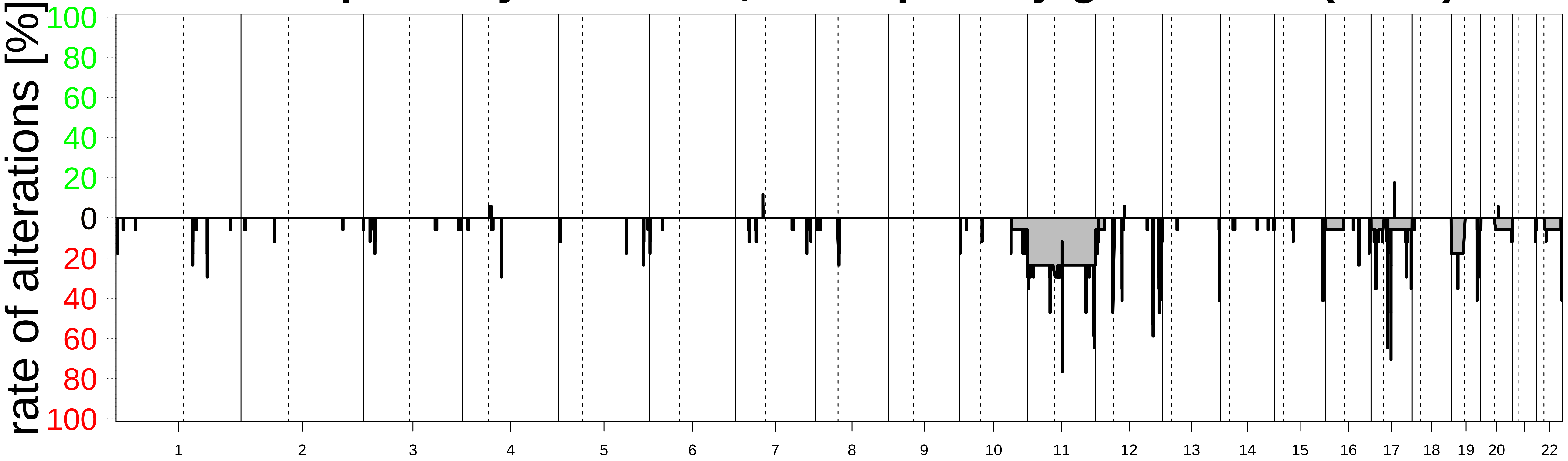

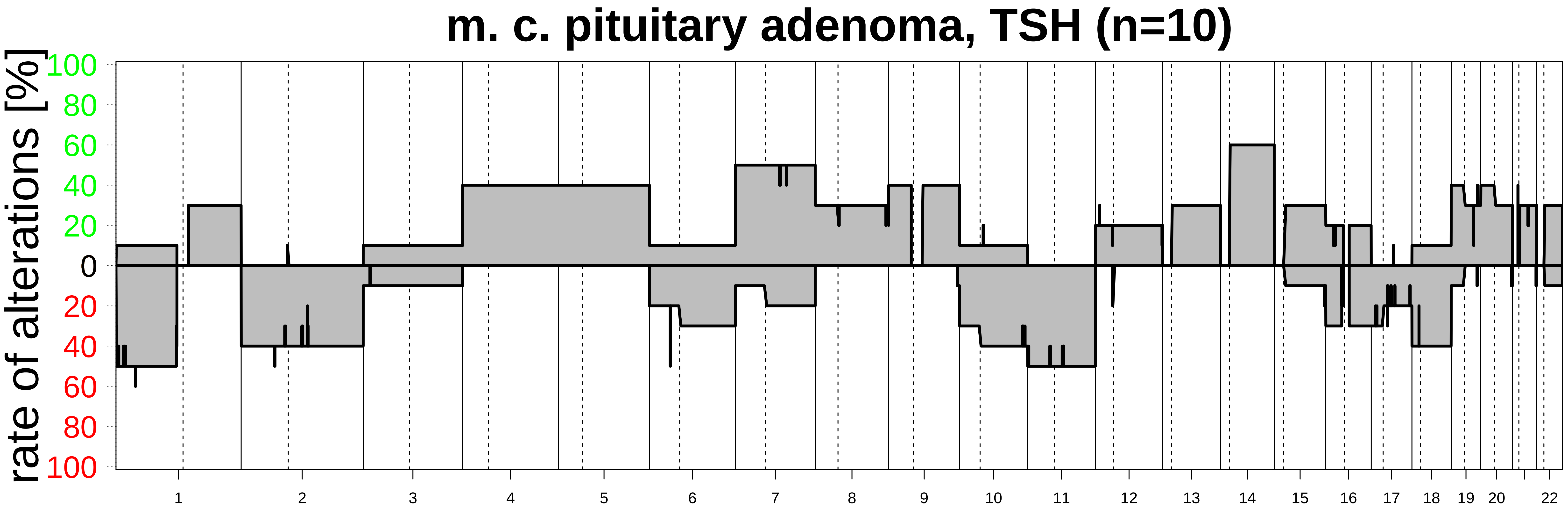

m. c. pituitary adenoma, TSH (n=10)
rate of alterations [%]
100
80
60
40
20
0
20
40
60
80
100
1
2
3
4
5
6
7
8
9
10
11
12
13
14
15
16
17
18
19
20
22

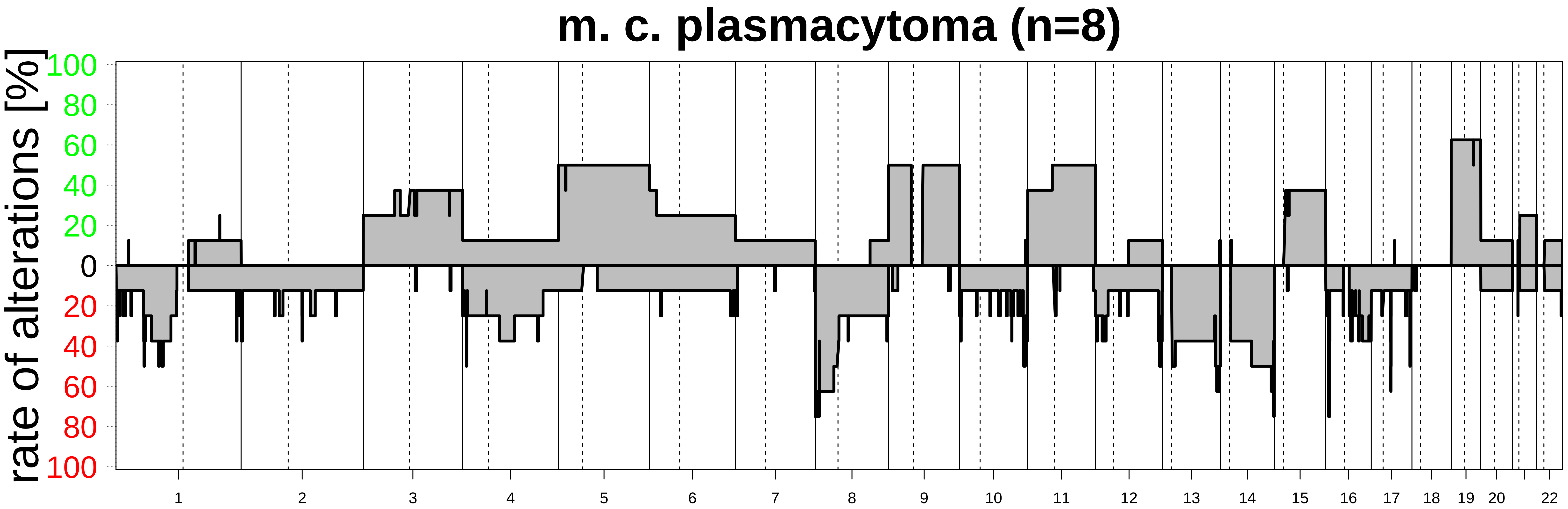
m. c. plasmacytoma (n=8)
rate of alterations [%]
100
80
60
40
20
0
20
40
60
80
100
1
2
3
4
5
6
7
8
9
10
11
12
13
14
15
16
17
18
19
20
22

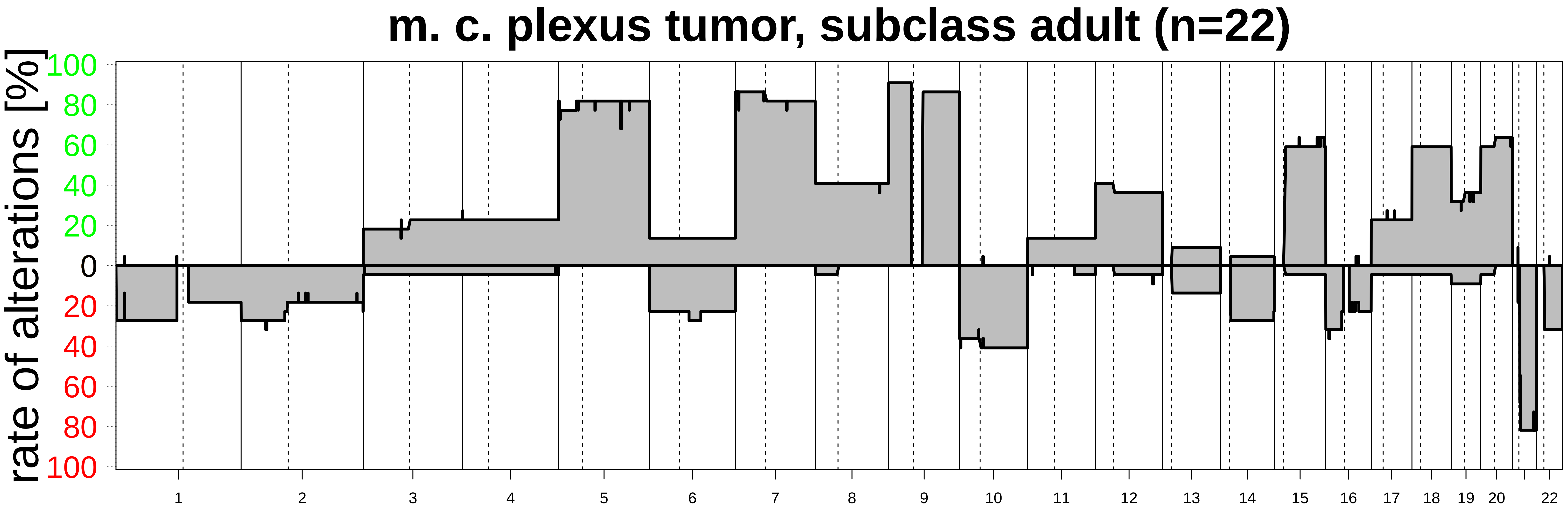
m. c. plexus tumor, subclass adult (n=22)
rate of alterations [%]
100
80
60
40
20
0
20
40
60
80
100
1
2
3
4
5
6
7
8
9
10
11
12
13
14
15
16
17
18
19
20
22

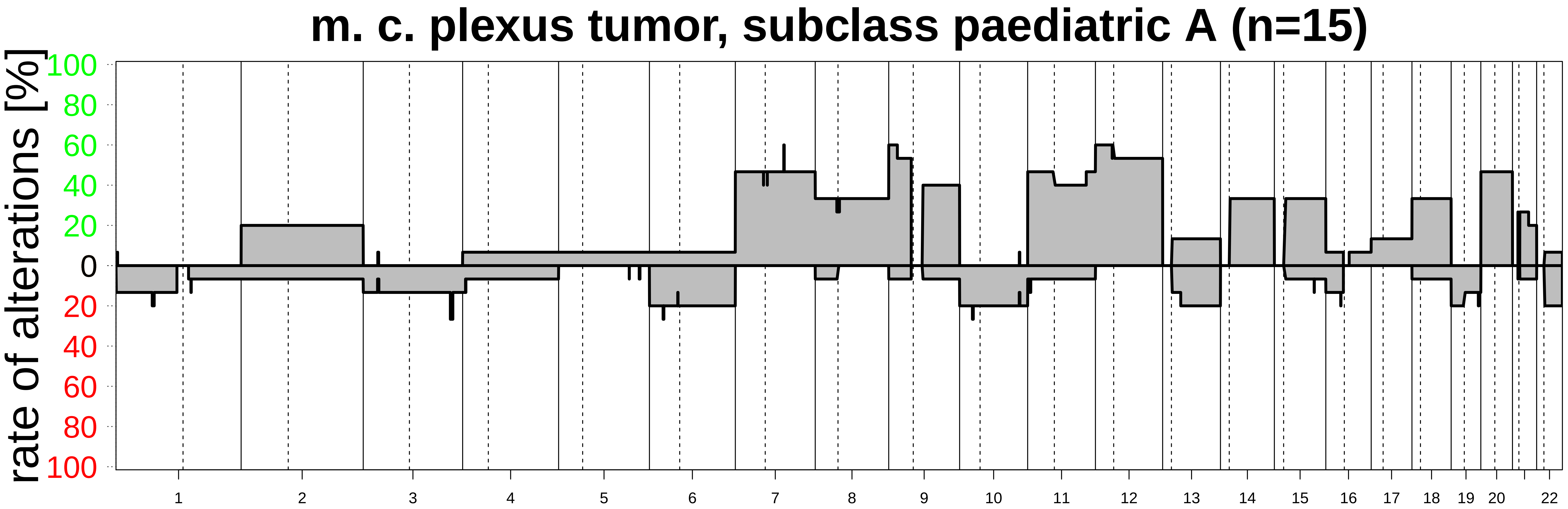
m. c. plexus tumor, subclass paediatric A (n=15)
rate of alterations [%]
100
80
60
40
20
0
20
40
60
80
100
1
2
3
4
5
6
7
8
9
10
11
12
13
14
15
16
17
18
19
20
22

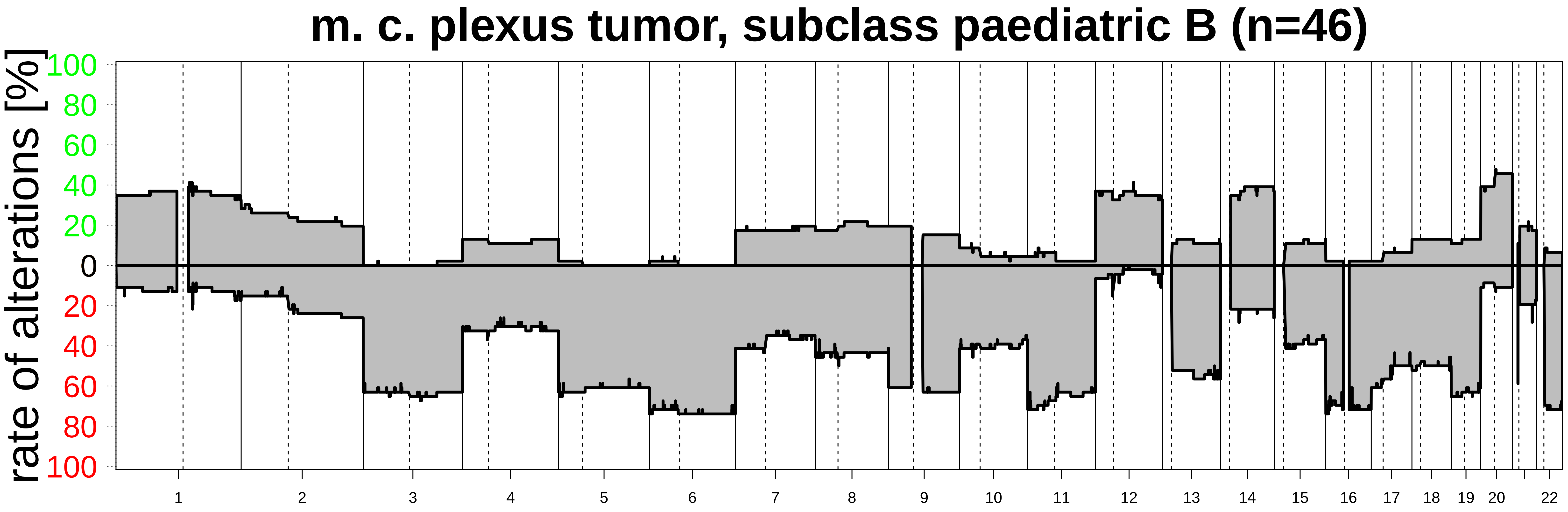

m. c. plexus tumor, subclass paediatric B (n=46)
rate of alterations [%]
100
80
60
40
20
0
20
40
60
80
100
1
2
3
4
5
6
7
8
9
10
11
12
13
14
15
16
17
18
19
20
22

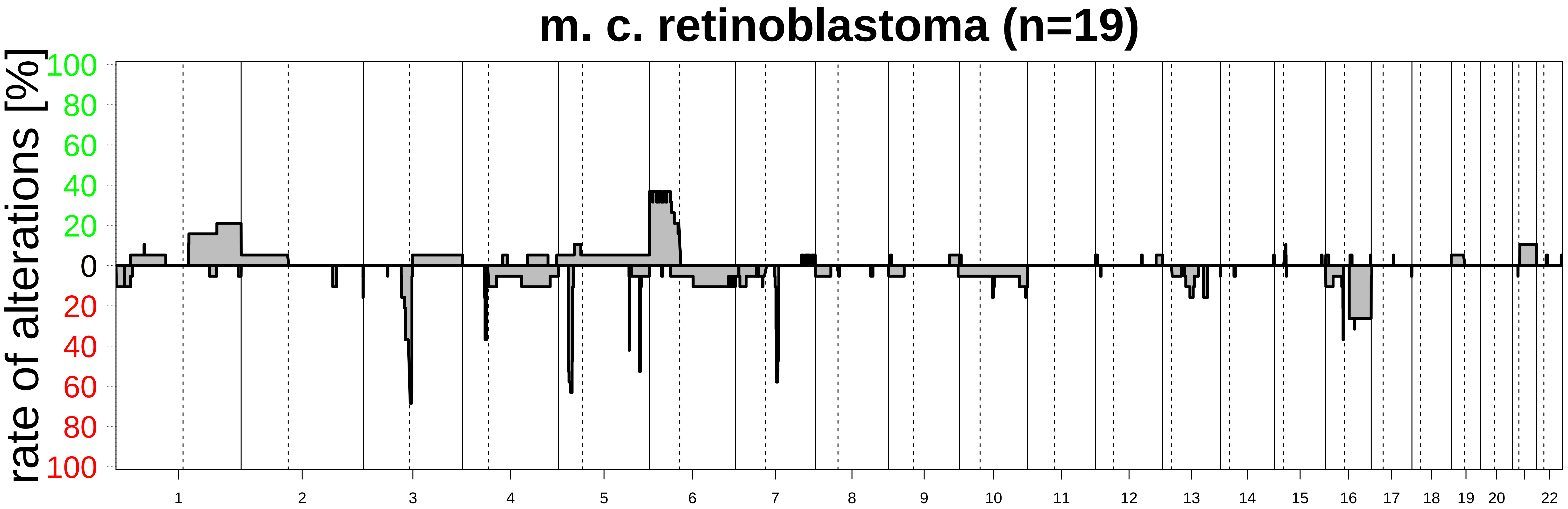
m. c. retinoblastoma (n=19)
rate of alterations [%]
100
80
60
40
20
0
20
40
60
80
100
1
2
3
4
5
6
7
8
9
10
11
12
13
14
15
16
17
18
19
20
22

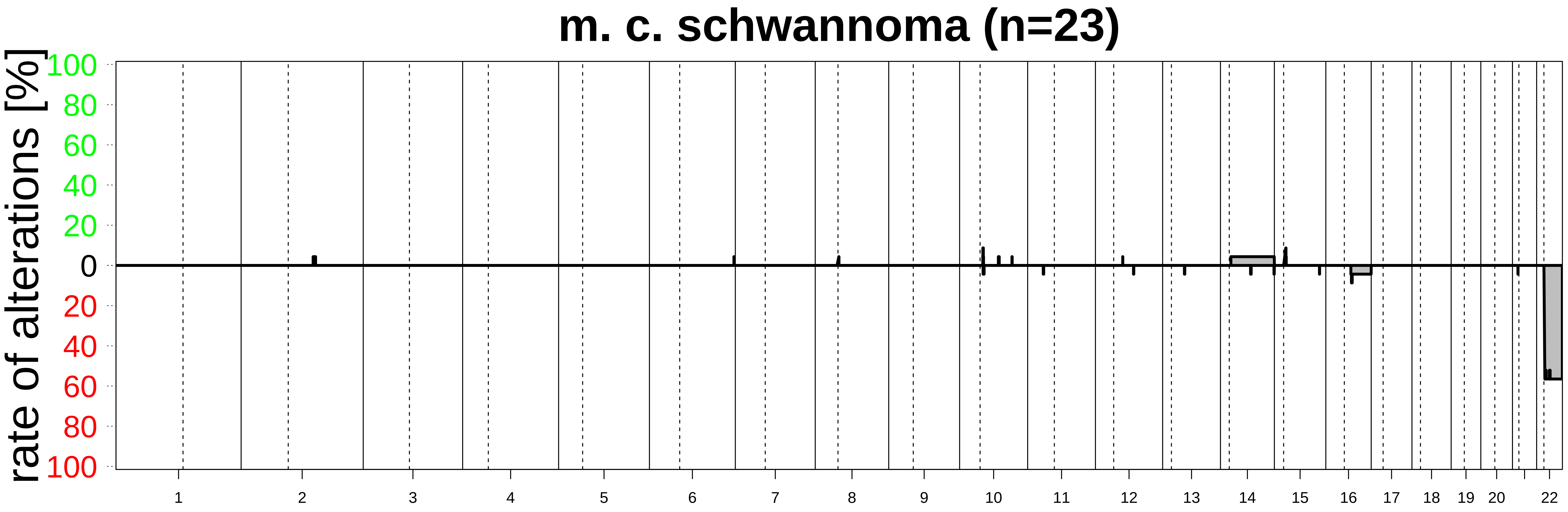

m. c. schwannoma (n=23)
rate of alterations [%]
100
80
60
40
20
0
20
40
60
80
100
1
2
3
4
5
6
7
8
9
10
11
12
13
14
15
16
17
18
19
20
22

m. c. solitary fibrous tumor − hemangiopericytoma (n=16)

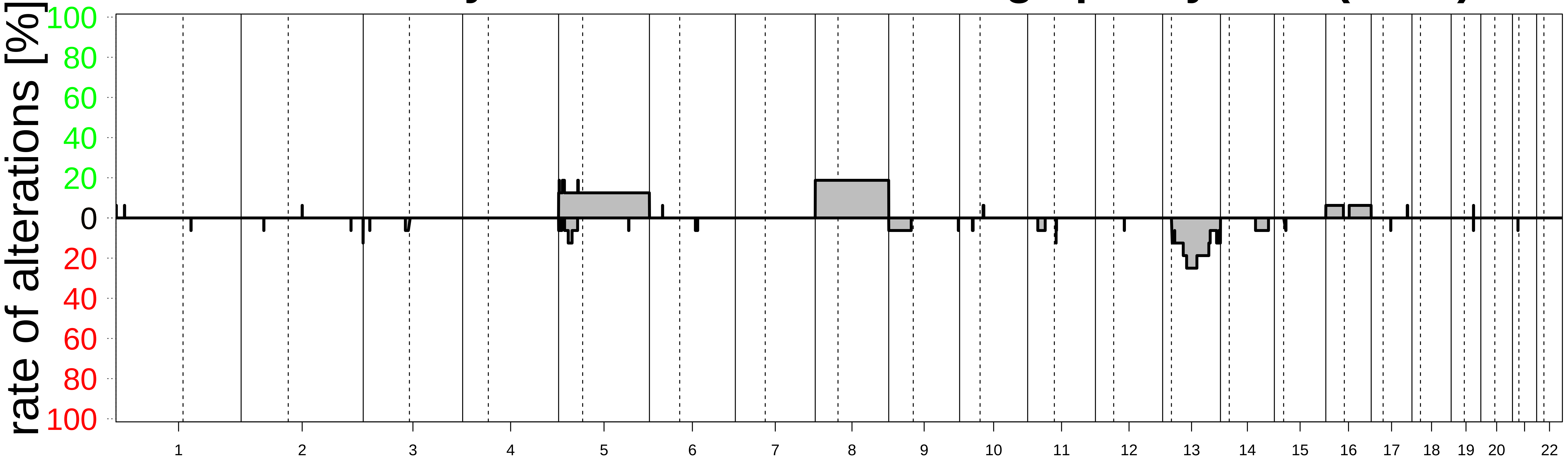

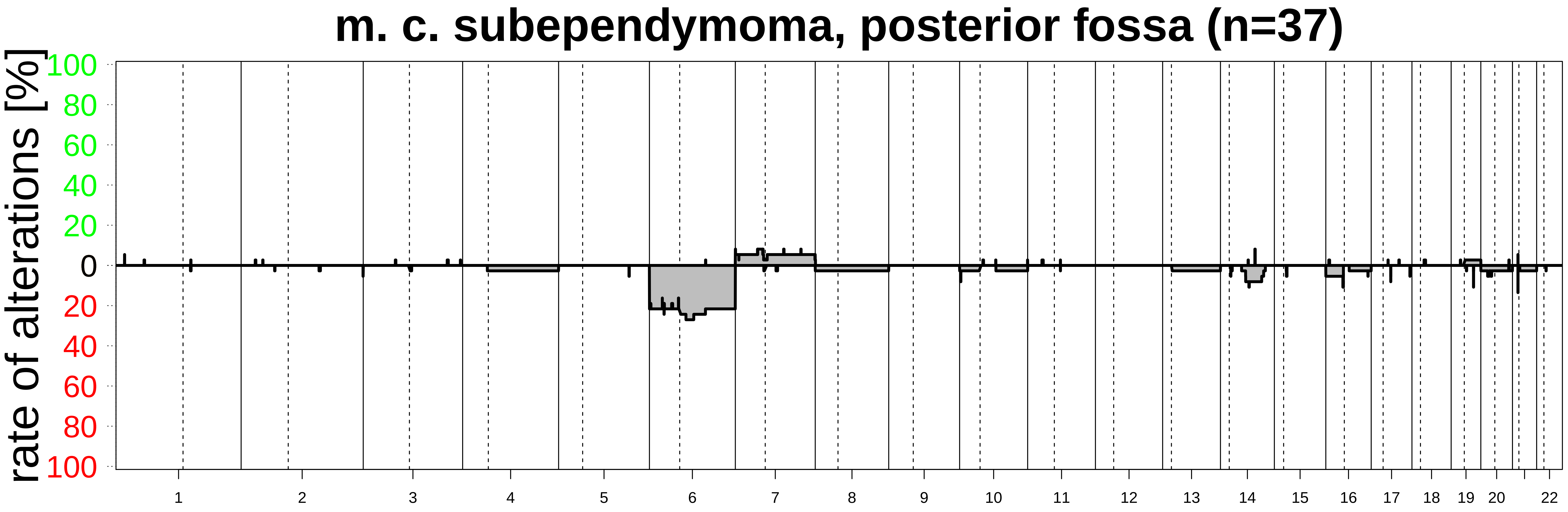
m. c. subependymoma, posterior fossa (n=37)
rate of alterations [%]
100
80
60
40
20
0
20
40
60
80
100
1
2
3
4
5
6
7
8
9
10
11
12
13
14
15
16
17
18
19
20
22

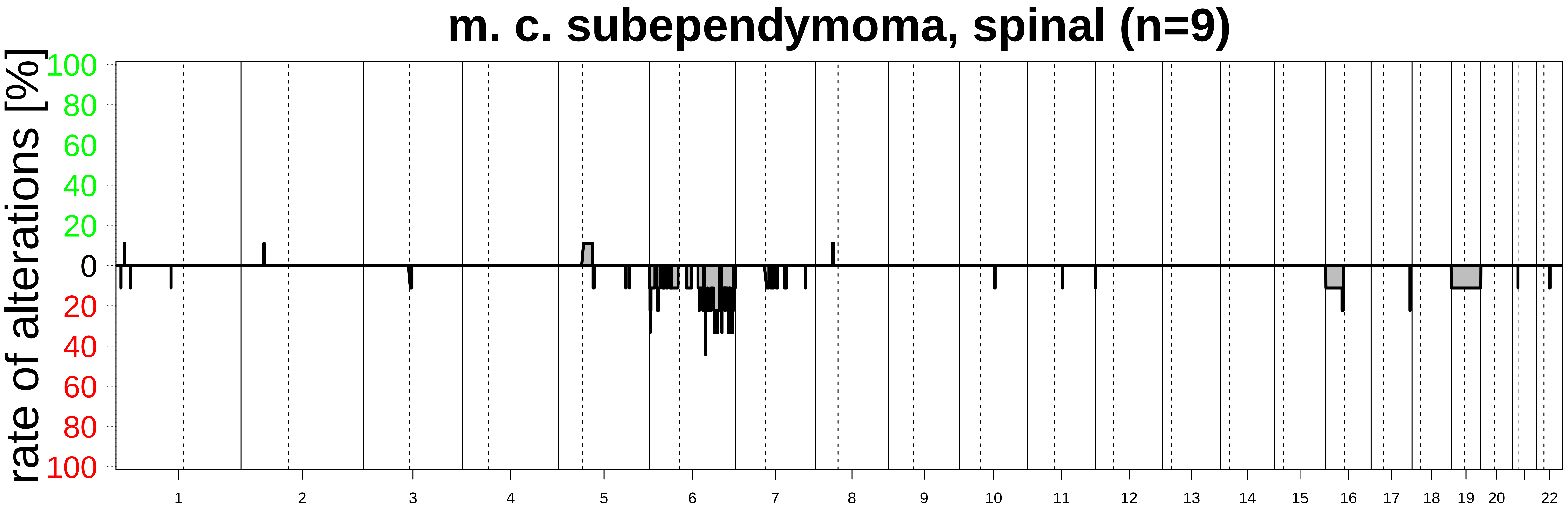
m. c. subependymoma, spinal (n=9)
rate of alterations [%]
100
80
60
40
20
0
20
40
60
80
100
1
2
3
4
5
6
7
8
9
10
11
12
13
14
15
16
17
18
19
20
22

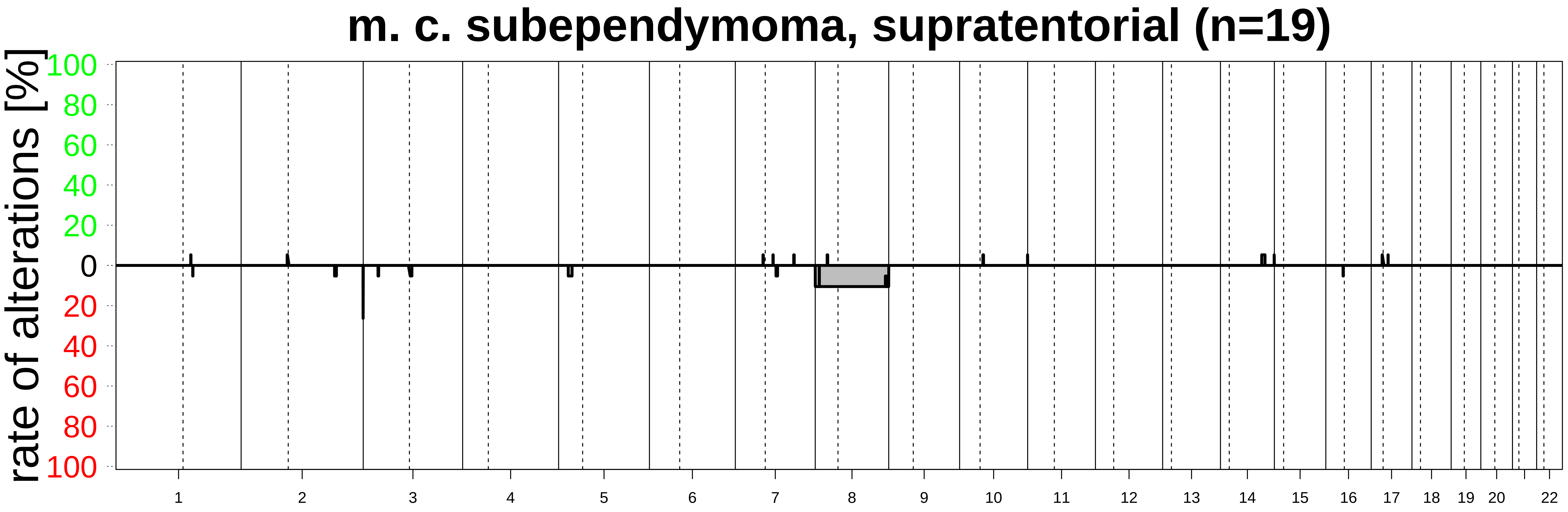
m. c. subependymoma, supratentorial (n=19)
rate of alterations [%]
100
80
60
40
20
0
20
40
60
80
100
1
2
3
4
5
6
7
8
9
10
11
12
13
14
15
16
17
18
19
20
22
